# The cost and cost-effectiveness of whole-exome and whole-genome sequencing: a systematic literature review

**DOI:** 10.1038/s41431-026-02146-2

**Published:** 2026-06-01

**Authors:** Frederick McElwee, Sally L. Sansom, James Buchanan, Jenny C. Taylor, Sarah Wordsworth

**Affiliations:** 1 Economics of Population Health Research Centre, Nuffield Department of Population Health, University of Oxford, Oxford, UK; 2 National Institute for Health Research, Oxford Biomedical Research Centre, University of Oxford, Oxford, UK; 3 Centre for Personalised Medicine, Nuffield Department of Medicine, University of Oxford, Oxford, UK; 4 Health Economics and Policy Research Unit, Centre for Evaluation and Methods, Wolfson Institute of Population Health, Queen Mary University of London, London, UK; 5 National Institute for Health Research, Barts Biomedical Research Centre, Queen Mary University of London, London, UK; 6 Centre for Human Genetics, Nuffield Department of Medicine, University of Oxford, Oxford, UK

**Keywords:** Economics, Health care economics, Genetic testing, Population screening, DNA sequencing

## Abstract

Existing systematic reviews of the health economic evidence for whole-exome and whole-genome sequencing (WES/WGS) are outdated or restricted to specific diseases or populations. This systematic review aims to provide an update on the health economic evidence for these sequencing options across a broad spectrum of clinical applications. We searched bibliographic databases (PubMed/EMBASE/MEDLINE/EconLit), the International Health Technology Assessment database, the Tufts cost-effectiveness analysis registry, and appraisals by the National Institute for Health and Care Excellence and the Scottish Medicines Consortium. We included studies reporting cost and/or cost-effectiveness results of WES and/or WGS, published between July 2016 and May 2024. Outcome-only studies were excluded. Summary information on study characteristics and findings was extracted, tabulated, and analysed. 130 studies were included. Availability of health economic evidence varied between geographic regions and clinical applications. 82 studies evaluated WES/WGS as diagnostic tools, but only five assessed prenatal, newborn, or healthy population screening. Many studies did not follow common methodological and reporting guidelines. Overall, WES/WGS can be cost-effective depending on the clinical and geographical context. The evidence base has grown significantly since 2016, but further evidence is needed, particularly for using these technologies for screening.

## Introduction

The costs of DNA sequencing have significantly declined since the advent of next-generation sequencing (NGS) in the mid-2000s [1, 2]. This decline in costs, along with improvements in throughput and speed, has transformed DNA sequencing from a predominantly research-focused tool into a more viable option for routine use in clinical practice [3]. Current applications primarily involve short-read sequencing approaches such as whole-genome sequencing (WGS) and whole-exome sequencing (WES). These vary in terms of the breadth of genomic information they provide and their cost. WGS determines the nucleotide sequence of an individual’s entire genome, covering protein-coding genes and non-coding regions. By comparison, WES targets only the protein-coding regions of the genome, the *exome*, where the majority of known disease-causing variants are located and is generally less expensive than WGS.

The main uses of WES and WGS range from diagnosing and informing the treatment of cancers and rare diseases, to screening newborns and ‘healthy’ populations. Many large-scale initiatives have been undertaken in these areas, such as the 100,000 Genomes Project, which sequenced the whole genomes of approximately 100,000 patients with cancers and suspected rare diseases [4]. This project led to the introduction of the Genomic Medicine Service (GMS) within the United Kingdom (UK) National Health Service (NHS) and the routine use of WGS to inform the diagnosis and treatment of some cancers and rare diseases [4]. Other prominent sequencing initiatives include the Australian Genomics Health Alliance Acute Care Genomics Study, where a national network used ultra-rapid DNA sequencing to inform the diagnosis of critically ill children and various newborn screening studies such as BabySeq in the United States (US) and the Generation Study in England [5–7].

While these and other, large-scale initiatives have contributed to the integration of DNA sequencing into mainstream clinical practice in some countries [3], uncertainty remains about the value for money provided by sequencing across the broader spectrum of disease prevention, diagnosis and treatment [8, 9]. Without evidence of the incremental costs and benefits of WES/WGS, it is difficult for health care systems to make decisions made about whether, when and how to recommend or reimburse these technologies.

We previously conducted a systematic literature review of economic evaluations of WES/WGS published between 2005 and 2016 [10]. The review concluded that estimates of cost and cost-effectiveness varied and that health economic evidence for both sequencing approaches was ‘very limited’. This review is almost a decade old and since then, sequencing methods have improved and large sequencing initiatives have generated additional data. Consequently, the findings in the previous review are unlikely to reflect the current health economic evidence base for WES/WGS. Although more recent systematic reviews have been conducted, these only provide an incomplete summary of the literature because they are confined to specific clinical contexts or patient subgroups, or only consider certain study types [11–14]. We aim to fill this evidence gap by undertaking and reporting a systematic literature review from 2016 onwards of economic evaluations assessing the use of WES/WGS across a broad spectrum of potential applications.

## Materials and methods

The review was pre-registered on the International Prospective Register of Systematic Reviews (PROSPERO) (CRD42024560439). No major amendments were made after registration.

### Search strategy

The review search strategy was modified from our prior review to encompass a broader range of databases and to focus on cost and cost-effectiveness studies. Searches were performed using four bibliographic databases (PubMed, EMBASE, MEDLINE, EconLit), the International Health Technology Assessment (HTA) database and the Tufts Cost-Effectiveness Analysis (CEA) registry. Searches were also conducted on health technology appraisals published by two UK health technology assessment bodies: the National Institute for Health and Care Excellence (NICE) and the Scottish Medicines Consortium (SMC). For NICE appraisals and SMC advice, publicly available guidance documents and committee papers were reviewed. Search details are provided in *S.A.1*.

To supplement the main search, we searched Google Scholar to identify whether corresponding full-text articles were available for relevant conference abstracts. Additionally, backward reference searches (‘snowballing’) were conducted on reviews and meta-analyses. These included reviews of health economic evidence or reviews with overlapping areas of focus that were published after the search cut-off date [15].

### Eligibility criteria

Studies published in English between 28 July 2016 and 7 May 2024 were included. Eligible studies were cost studies or economic evaluations of the use of WES and/or WGS to inform patient-level decisions. Eligible study types included cost studies, cost-effectiveness studies, cost-utility studies, cost-benefit analyses, cost-consequence studies and cost-minimisation studies. Eligibility was not restricted by testing purpose, disease area, or type of results returned. The Population-Intervention-Comparison-Outcomes (PICO) criteria are summarised in *S.A.2*.

We excluded: (1) studies only reporting outcomes (e.g. diagnostic yield, patient-reported outcomes) without reporting either costs (healthcare costs or cost per sample) or a measure of cost-effectiveness (e.g. cost per QALY gained, cost per additional diagnosis); (2) studies only reporting sequencing costs associated with a particular instrument set-up or workflow (e.g. library preparation) without clinically relevant applications; (3) studies only assessing WES/WGS of infectious disease pathogens in the context of population-level surveillance, (4) preference elicitation studies assessing the willingness to pay for WES/WGS without estimating cost or cost-effectiveness; and (5) studies only analysing RNA sequencing (e.g. transcriptome sequencing).

Reviews and meta-analyses were subject to the same eligibility criteria. We did not place restrictions on the type of review or meta-analysis performed.

### Study selection and data extraction

Search results were exported to EndNote 21. The eligibility criteria were piloted against a subset of de-duplicated articles before proceeding with the full review. The pilot stage uncovered some studies that reported costs in the full article or supplementary appendices but did not indicate this in the title or abstract. Given this, the full-text articles were scanned for sections containing cost information when completing the title and abstract review. This process was performed by FM. A random sample of 10% of all de-duplicated articles was also screened by SW to validate the selection process. Studies selected for full-text review were reviewed both by FM and SW. Results were presented using the Preferred Reporting Items for Systematic reviews and Meta-Analyses (PRISMA) flow diagram [16].

FM extracted information from the included full-text articles into Microsoft Excel based on a template adapted from Schwarze et al. [10] following a pilot involving 10 articles. The following data fields were extracted where relevant: costs; incremental cost-effectiveness ratio (ICER); study type; clinical context; sequencing approach; age restriction of study population; study setting (country); study perspective; comparators; discount rate; time horizon; sample size; publication year; costing approach; and information on study quality and reporting. Clinical context was classified into six categories, defined in *S.A.3*. SW verified data extracted by FM. SLS served as the tie-breaker.

### Data analysis and reporting

Costs were converted using purchasing power parities (PPPs) to 2024 Great British Pounds (GBP) and US Dollars (USD) (details in *S.A.4)*. We expected the cost and cost-effectiveness of WES/WGS to vary by country, between clinical applications and over time. We also anticipated variation in the scope of included costs (e.g. per-sample cost, total cost of care) and study perspective. Given this, we did not aggregate cost-effectiveness estimates across studies. While sequencing cost estimates from micro-costing analyses vary based on the platform used and time period considered, there are fewer confounders for these estimates than there are for estimates of total costs [17, 18]. We therefore indicated a range of estimated WES/WGS costs from these studies.

### Quality of reporting

We evaluated the quality of reporting of included studies using an abbreviated list of criteria corresponding to 5 of the 28 items on the 2022 Consolidated Health Economic Evaluation Reporting Standards (CHEERS) checklist (items 6,8-10,15) [19]. FM completed the assessment for each included study. For reviews, we recorded whether the inclusion and exclusion criteria were explicitly reported and whether a risk of bias assessment was performed.

## Results

**Fig. 1:**
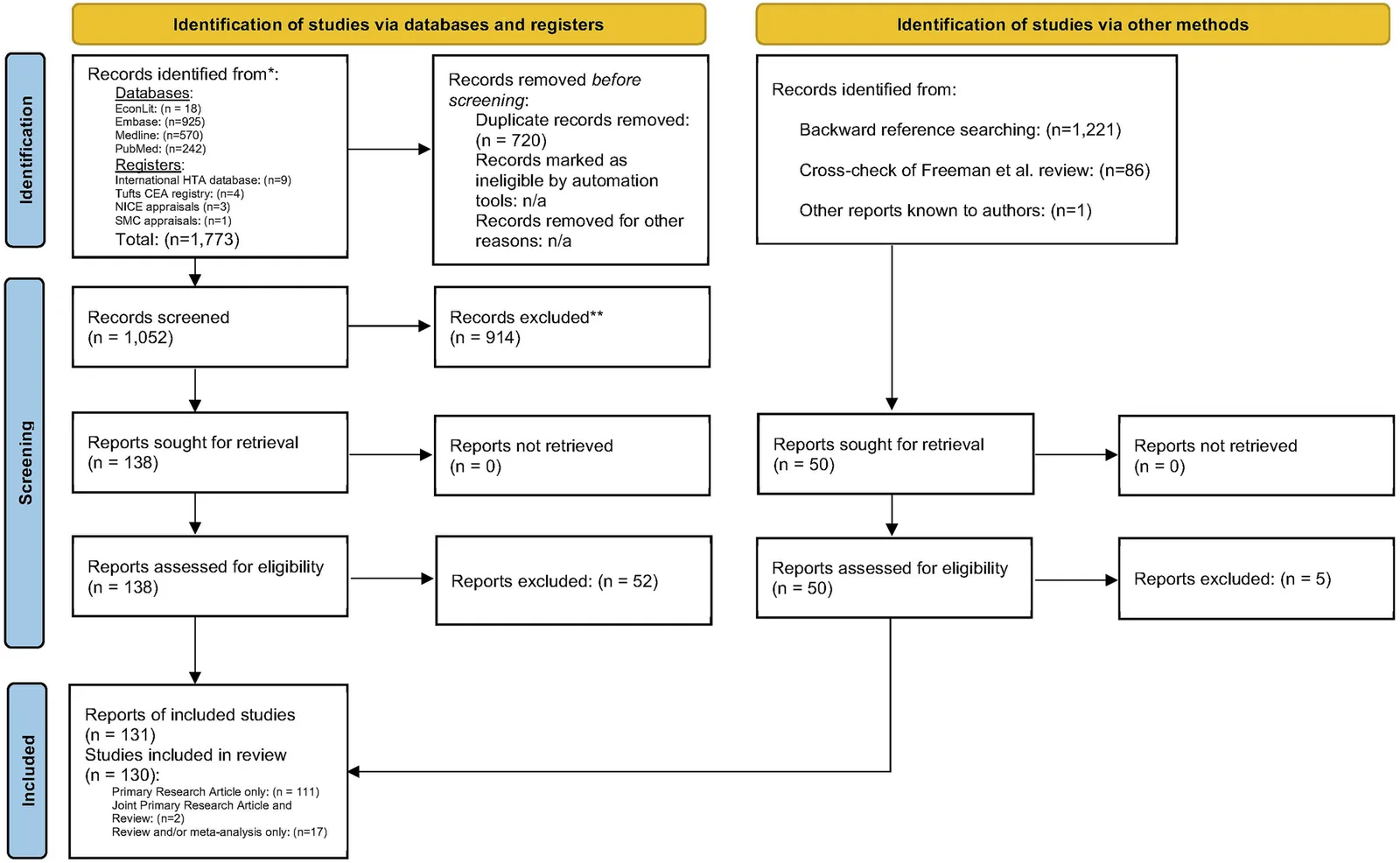
Flow diagram of literature search result (Source: Page MJ, et al. BMJ 2021; 372:n71. https://doi.org/10.1136/bmj.n71.).

1773 records were identified, of which 720 were duplicates (Fig. 1). After full-text review, 86 reports were included. A further 45 studies were identified using other means, such as backward-referencing. A total of 131 reports representing 130 studies were included.

### Primary research studies

A total of 113 primary research studies were included. For eight studies, only an abstract was published. Table 1 contains summary information about these studies. Study-level information is presented in *S.A.5*. A summary table excluding abstract-only studies is presented in S.A. 6.

**Table 1:** Study characteristics of primary research publications.

Category	Sub-Category	Number of Studies (%)
Year of Publication	2016^a^	6 (5.3%)^a^
	2017	11 (9.7%)
	2018	14 (12.4%)
	2019	7 (6.2%)
	2020	17 (15.0%)
	2021	22 (19.5%)
	2022	19 (16.8%)
	2023	13 (11.5%)
	2024^a^	4 (3.5%)^a^
Country	United States of America	23 (20.4%)
	United Kingdom	7 (6.2%)
	Australia	25 (22.1%)
	Europe (excl. UK)	25 (22.1%)
	Canada	17 (15.0%)
	China (incl. Hong Kong SAR)	7 (6.2%)
	Country not listed above	6 (5.3%)
	Multiple countries	2 (1.8%)
	Not reported	1 (0.9%)
Type of Economic Evaluation	Cost-Utility Analysis (CUA)	14 (12.4%)
	Cost-Effectiveness Analysis (CEA)	46 (40.7%)
	Cost-Consequence Analysis (CCA)	39 (34.5%)
	Cost Study	14 (12.4%)
Sequencing Type	Both human WGS and WES	17 (15.0%)
	Human WGS only	22 (19.5%)
	Human WES only	60 (53.1%)
	Pathogen WGS only	14 (12.4%)
Application^c^	Diagnostic tool only	82 (72.6%)
	Pathogenic screening only	14 (12.4%)
	Precision oncology only	11 (9.7%)
	Prenatal screening only	2 (1.8%)
	Newborn screening only	0 (0.0%)
	Healthy population screening only	0 (0.0%)
	Prenatal screening & diagnostic tool	1 (0.9%)
	Health population screening & diagnostic tool	1 (0.9%)
	Precision oncology & diagnostic tool	1 (0.9%)
	Newborn screening & diagnostic tool	1 (0.9%)
Discount Rate^c^	>0%, <1.5%	0 (0.0%)
	≥1.5%, <3%	5 (4.4%)
	≥3%	15 (13.3%)
	Different discount rate categories applied to costs and effects	5 (4.4%)
	Not applied / Not specified	88 (77.9%)
Study Perspective^c^	Societal	5 (4.4%)
	Health system, hospital, or payer^b^	70 (61.9%)
	Cost of testing only	12 (10.6%)
	Multiple/Other	26 (23.0%)
Time Horizon^c^	<1 year	4 (3.5%)
	≥1 year, <5 years	18 (15.9%)
	≥5 years, <10 years	5 (4.4%)
	≥10 years, <25 years	5 (4.4%)
	≥25 years, but not lifetime	1 (0.9%)
	Lifetime	3 (2.7%)
	Multiple/other	9 (8.0%)
	Unclear; not reported, applicable, or specified	68 (60.2%)
Currency^c^	Report currency	104 (92.0%)
	Report currency year	65 (57.5%)
	Currency year reported inconsistently or only for certain cost elements	2 (1.8%)

*SAR* special autonomous region, *WGS* whole genome sequencing, *WES* whole exome sequencing, *UK* United Kingdom.

^a^The search period was 28 July 2016 to 7 May 2024.

^b^Studies that report both health system and personal budget perspectives are included under this category.

^c^Items in the CHEERS checklist.

### Micro-costing analyses of WES/WGS

Seven studies (five cost-studies, two CEAs) presented micro-costing analyses of WES/WGS of human DNA. Some studies briefly reported costs by category and aggregated these into a total sequencing cost, but were not detailed micro-costing studies [20–23]. Other studies applied a micro-costing approach to estimate the costs of specific steps in the sequencing process, but did not apply this approach to cost the whole testing process [24, 25]. All studies included the cost of sequencing itself, bioinformatics and clinical interpretation; some also included reporting.

Cost estimates varied considerably by country, clinical context and throughput. The highest and lowest estimated costs for WES of human DNA were €3842 (2024 GBP£4104) per pair and €804 (£842) per sample by Bayle et al., respectively [17]. The highest and lowest estimated costs for trio-WGS of human DNA were GBP£7050 (2024 GBP£9118) per trio by Schwarze et al. [26] and CAD$6556 (£4629) per trio by Jegathisawaran et al. [27]. On a singleton-WGS or per-genome basis, the highest and lowest estimated costs were £3420 (£4423) by Schwarze et al. [26] and CAD$2851 (£2114) by Tsiplova et al. [28]. The studies reporting the highest and lowest WES/WGS cost estimates were consistent when PPP and pure exchange rates were used. *S.A.7* provides further summary information about micro-costing estimates.

Pasmans et al. estimated the cost of germline and somatic WGS for precision oncology in the Netherlands to be €2925 per patient (£3191) [29]. This study also performed a micro-costing of relevant comparators such as CMA, FISH, gene panel tests and Sanger sequencing. Schwarze et al. estimated the cost of WGS for precision oncology across a range of cancer types to be £6841 (£8848) per patient, inclusive of both germline and somatic sequencing, or £3420 (£4423) per genome [26]. They also estimated the cost of WGS for diagnosing rare disease as GBP£7050 (£9118) per trio, or GBP£2350 (£3039) per genome. Abbott et al. reported a similar cost of trio-WGS for rare disease diagnosis in Scotland to be £6625 in 2018 (£8274) [30]. In addition, Jegathisawaran et al. estimated the cost of trio-WGS to be CAD$6634 (£4612) when used to diagnose developmental delay and CAD$8035 (£5586) when used to diagnose paediatric cardiac conditions in the Canadian setting [31]. Another study by the same team forecast that the latter cost (trio-WGS for paediatric cardiac conditions) would decrease to CAD$7576 (£5267) within five years due to scale economies [32].

Studies that reported micro-costing estimates for both WES and WGS consistently found WGS to be more costly. Tsiplova et al. performed a micro-costing of WES and WGS for the diagnosis of autism spectrum disorder (ASD) [28]. They estimated costs per sample were as follows: CAD$1655 (£1227) for WES; CAD$5519 (£4092) for WGS on HiSeq 2500; and CAD$2851 (£2114) for WGS on HiSeq X. Jegathisawaran et al. estimated that the cost of WES for diagnosing ASD varied between CAD$1960-$1981 (£1384-£1399) for the proband alone and CAD$3143-$4072 (£2220-£2876) per trio-WES, depending on the platform used. They also estimated the cost of WGS to be CAD$3350 (£2366) for the proband alone and CAD$6556 (£4629) per trio [27]. Gordon et al. estimated the cost of sequencing for precision oncology in Australia to be AU$871–$2788 (£507-£1622) per person for WES and AU$2895–$4830 (£1685-£2811) per person for WGS [33]. When capital costs were included, estimated costs were AU$963-$2984 (£560-£1737) for WES and AU$3007-$4924 (£1750-£2865) for WGS.

One micro-costing study, Bayle et al., estimated the cost of WES but not WGS, demonstrating substantial cost reductions over time due to advances in sequencing platforms. The study found that the cost of WES for precision oncology in France decreased from €1921 (£2052) per sample in 2015 when a HiSeq 2000 sequencer was used to €804 (£842) per sample in 2018 when a NovaSeq 6000 sequencer was used [17]. The total sequencing costs per oncology patient (two samples) were estimated to be €3842 (£4104) in 2015 and €1608 (£1683) in 2018.

Two studies performed full micro-costing analyses for pathogen WGS. Mellmann et al. estimated the cost of WGS of multi-drug resistant bacteria to be €202.49 (£239) per isolate [34]. Kamboj et al. estimated the cost of WGS of *C. difficile* to be USD$164 (£135) per isolate [35]. These are consistent with values found in other economic evaluations that used prices or estimates from the literature, which ranged from US$70 to AU$355.

### Findings by clinical application

#### Diagnostics

The most common clinical application of WES/WGS was as a diagnostic tool. In all six CUAs evaluating this application of sequencing (five WES, one WGS), at least one strategy involving WES/WGS was cost-effective compared to standard of care in at least one population analysed in the base case. Two CUAs found WES/WGS or WGS re-analysis to be cost-saving [36, 37].

Forty-one CEAs evaluated sequencing as a diagnostic tool: four of WGS only, 23 of WES only and 15 involving both WGS and WES. Twenty-seven CCAs evaluated sequencing as a diagnostic tool: six of WGS only and 21 of WES only. With one exception [20], all CCAs had a comparator (e.g. standard of care, alternative sequencing type) or compared costs before and after diagnosis with WES/WGS. The CEAs and CCAs were conducted in different geographical regions and in patients of different ages. The remaining eight studies were cost studies: three of WGS only, four of WES only and one involving both WGS and WES.

#### Screening

One CCA assessed clinical applications of WGS in newborn screening [38]. Another study assessed using WGS for healthy population screening (alongside other clinical applications) [24]. It found that sequencing costs themselves affected total costs by more than downstream healthcare costs. Three studies analysed using WES for prenatal screening.

#### Precision oncology

Three CUAs, one CCA and one cost study were conducted on the use of WGS for precision oncology in the Netherlands. The CUAs found that WGS was not cost-effective for diagnosis in the base case, but could be under various plausible scenarios, such as lower WGS costs. The remaining studies were either cost studies or CCAs.

#### Pathogens

Seven of 14 studies that analysed pathogen WGS were set in Australia. One study was an application to the Medical Services Advisory Committee (MSAC) in Australia recommending that WGS be used for antimicrobial susceptibility testing of *M. tuberculosis* [39]. The other studies simulated and/or used data from facilities within Queensland’s public hospital system. All found that WGS was likely to be cost-saving and more effective in averting infections. Seven other studies analysed pathogen WGS in the US, UK and Europe. All three CUAs concluded that strategies containing pathogen WGS were cost-effective.

### Study statistics, quality of reporting and study errors

#### Sample size

Many studies had small sample sizes, which is common for rare disease studies. The study with the lowest sample size analysed trio-WES for 4 index cases (12 exomes) [40]. Other studies analysed results from more patients, but only a subset underwent DNA sequencing. For example, one study analysed sequencing data for 1433 cancer patients, although only 496 (34.6%) patients had WES/WGS [33]. Another study analysed 1443 individuals, of which 194 (13.4%) received genetic diagnostic testing [41]. Studies evaluating pathogen sequencing tended to be larger: Dymond et al. analysed 2282 MRSA isolates[42] and Gordon et al. (2021) analysed 1783 bacterial isolates [43].

#### Quality of reporting

Most studies did not report important information about study methods, such as the discount rate and time horizon. Ninety-two percent of studies reported the currency used, but 42% did not report the currency year. Reports only published as an abstract often did not contain methodological details that would be expected in a full-text article [44–46]. Some studies that reported costs associated with WES/WGS or implications for total healthcare costs provided little detail about how these were estimated or from where they were sourced (e.g. [22, 47–51]).

#### Issues with study analysis or conclusions

Several studies did not follow best practices for analysing cost-effectiveness. Eight compared the costs/outcomes of WES/WGS to comparators individually (e.g. to the standard of care) rather than evaluating options incrementally by comparing to the next-best non-dominated option or by ranking options by net benefit. One study miscalculated the ICER based on reported costs and QALYs, but made correct cost-effectiveness conclusions [52]. Three studies mislabelled cost-effectiveness outcomes: the first conflated the ICER with the average cost per diagnosis and did not correctly estimate cost-effectiveness outcomes [53]; the second presented an estimate of the cost saving per patient tested when what was calculated was a reduction in mean cost per diagnosis [45]; and the third reported the price at which WES becomes cost-effective when it actually estimated the price at which WES becomes cost-saving [54]. Four CEAs reported cost per diagnosis in the intervention and comparator arms, but not cost per additional diagnosis when the intervention was not dominant [55–58]. Two studies draw cost-effectiveness conclusions assuming that the willingness to pay (WTP) per QALY and WTP per additional diagnosis are equivalent, which is unlikely [59, 60].

### Reviews and meta-analyses

Nineteen review or meta-analytic articles were included: 15 literature reviews, one meta-analysis, two studies that combined primary research with a literature review and one joint review and meta-analysis. Table 2 contains summary information about these studies. Study-level information is in *S.A.8*.

**Table 2:** Study characteristics of reviews and meta-analyses.

Category	Sub-Category	Number of Studies (%)
Year of Publication	2016^a^	1 (5.3%)^a^
	2017	1 (5.3%)
	2018	4 (21.1%)
	2019	2 (10.5%)
	2020	1 (5.3%)
	2021	1 (5.3%)
	2022	6 (31.6%)
	2023	3 (15.8%)
	2024^a^	0 (0.0%)^a^
Sequencing Type	Both human WGS and WES	13 (68.4%)
	Human WGS only	1 (5.3%)
	Human WES only	2 (10.5%)
	Pathogen WGS only	2 (10.5%)
	Both Pathogen WGS and Human WES/WGS	1 (5.3%)
Disease Context	Diagnostic tool	12 (63.2%)
	Pathogenic screening	2 (10.5%)
	Precision oncology	2 (10.5%)
	Multiple/no restriction	3 (15.8%)
Age Restriction of Search	Paediatrics only	5 (26.3%)
	Mix/no age restriction applied	14 (73.7%)

*WGS* whole genome sequencing, *WES* whole exome sequencing.

^a^Only parts of 2016 and 2024 were within the timeframe of publication considered.

One review only considered micro-costing analyses [61], while the others considered both costs and outcomes. Two reviews imposed geographical restrictions: one article by Norris et al. only reviewed decisions by the Australian HTA body (MSAC) related to DNA sequencing, including WES/WGS [62]; a second article by Weymann et al. reviewed economic evaluations of WES/WGS in Canada [63]. Two reviews imposed age-based restrictions on the patient populations analysed by studies, both of which only included studies of paediatric populations [11, 64]. Reviews that impose geographic or age restrictions can synthesise evidence about a specific region or age group, but these findings may have limited generalisability beyond the context considered by the review.

Four studies placed no restrictions on the type of clinical application assessed by economic evaluations or considered multiple applications. Eleven publications reviewed studies of human WES/WGS as a diagnostic tool and one study reviewed evaluations of human WES/WGS for precision medicine.

Several studies highlighted a lack of economic evidence for the use of WES/WGS in specific clinical applications. Of the 19 reviews identified, only five included at least ten economic evaluations. The review with the highest number of economic evaluations included 32 studies [12]. In contrast, one review identified only two studies, neither of which evaluated cost or cost-effectiveness [65].

#### Pathogen WGS

Three reviews considered pathogen WGS: first, Price et al. report a range of per-sample WGS costs and net savings to healthcare systems [13]. All included studies favoured the use of WGS over comparators. Second, Tran et al. report minimum and maximum values for costs and outcomes and note the difficulty in generalising results to draw conclusions about the broad adoption of bacterial WGS, as results are pathogen-specific [66]. Last, Schwarze et al. included studies of both pathogen and human WGS. The review reported minimum and maximum costs for bacterial WGS separately from human WGS and found that the range of costs for bacterial WGS was lower than that for human WGS [10].

#### Incidental and/or secondary findings

One systematic review, authored by Fontes Marx and colleagues, reviewed studies that estimated the cost-effectiveness of reporting incidental and/or secondary findings from WES/WGS [67]. They included six economic evaluations and found limited and mixed evidence of the cost-effectiveness of reporting these findings. While patients often value incidental and secondary findings, the cost of generating these results varies by patient condition and setting, which limits generalisability.

#### Quality assessment of reviews

15 reviews defined a search strategy with explicit inclusion/exclusion criteria. Four reviews performed formal assessments of reporting quality or risk of bias. First, a review of economic evaluations of pathogen WGS by Tran et al. assessed risk of bias using the Consensus Health Economic Criteria (CHEC) and reported that >80% of included studies exhibited moderate/high risk of bias [66]. Another review by Fontes Marx et al. assessed study quality using the Q-Genie tool and risk of bias with an instrument adapted from two prior studies [67]; they concluded that all included studies were of moderate/high quality and exhibited a low/moderate risk of bias. Third, Alam et al. found that most studies did not meet most of the criteria for a well-designed economic evaluation according to the Drummond et al. checklist [11]. Last, Price et al. deemed studies to be ‘high quality’ overall using the Drummond-Jefferson checklist [13].

### Trends over time

Our previous review identified 36 full-text studies in total, of which 28 assessed costs or cost-effectiveness. (Eight studies were outcome-only studies, which were excluded in the updated review.) Six of these were published after the cut-off date (July, 2016), meaning that 22 were published from 2005 to July 2016. The current review (covering July 2016 to 2024) identified 131 studies, of which 123 were full-text cost or cost-effectiveness studies (112 primary research reports, 18 reviews or meta-analyses and one combined primary research study and review). Eight studies were abstracts with no corresponding full-text article, which would have been excluded from the prior review. This represents a fourfold increase in the number of full-text cost or cost-effectiveness studies in the period 2016–2024 compared to 2005–2016, or a fivefold increase on an annual basis. Figures of the time trends in the publication of economic evaluations of WES/WGS are included in *S.A.9 A/9B*.

**Fig. 2:**
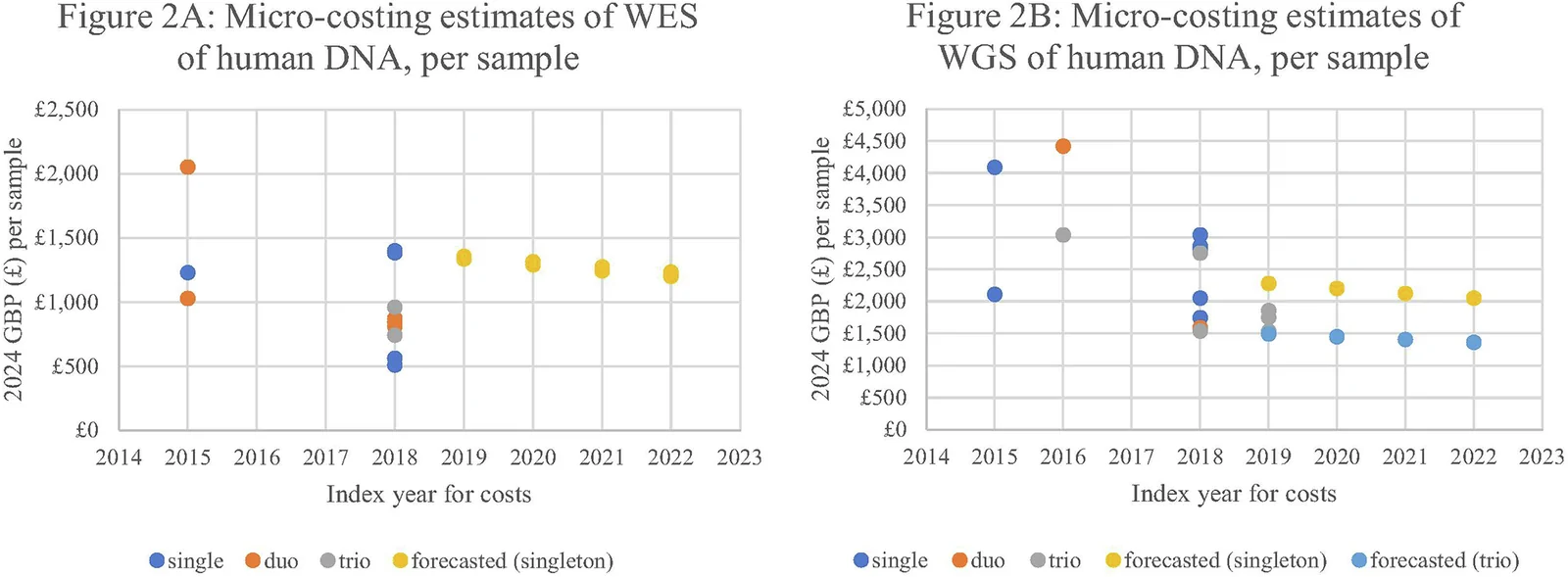
Time trends in the results of micro-costing studies, inflated using purchasing power parity (PPPs). Cost estimates for duos and trios were divided by two and three, respectively, to estimate the cost per sample. The 5-year forecast was made by Jegathisawaran, Tsiaplova et al. [31].

Compared with our prior review, the range of WES/WGS costs has narrowed and is entirely contained within the earlier range. We report scatter plots of micro-costing estimates for human WES/WGS against each study’s respective index cost year in Fig. 2. All micro-costing studies had index cost years that fell in the first half of the time period considered by this review: the index year of the most recent micro-cost estimates was 2019 for human WGS and 2020 for WES.

## Discussion

This systematic review aims to provide an update on the health economic evidence for WES/WGS across a broad spectrum of clinical applications. The availability and conclusions of economic evidence on the use of WES/WGS differ by the geographical and clinical context. Most studies focus on their application as diagnostic tools, with considerably less evidence available for their use in screening contexts. Reporting quality varies. Conclusions about the cost-effectiveness of different strategies using WES/WGS cannot always be taken at face value; not all studies follow best practices for evaluating cost-effectiveness, with some drawing incorrect or incomplete conclusions on the basis of the evidence presented.

### Strengths and limitations of the evidence base

The health economic evidence base for WES/WGS has expanded considerably since our previous review in 2016 [10]. Many studies found that using WES/WGS as a first- or second-tier diagnostic tool can be a cost-effective strategy. WES/WGS for pathogen screening is relatively inexpensive and generally cost-effective. While sequencing costs have declined over time, the rate of decrease has not been as fast as many expected and we did not identify updated micro-cost estimates published from 2020 onwards.

Additional high-quality research is still needed. To maximise the utility of this research for health economists and decision makers, studies should report a comprehensive set of health economic information, ideally adhering to standardised reporting guidance (e.g. the Consolidated Health Economic Evaluation Reporting Standards checklist [19]). Many included studies did not report information required by the abbreviated list of CHEERS criteria considered in our review. When costs are reported, for example, details should be provided about how they were estimated or from where they were sourced. Some of the studies that did not report these details were probably not intended to be formal economic evaluations, even though they met our inclusion criteria by reporting costs (e.g. [20, 22, 35, 38, 48]). Investigators who seek to perform similar studies in the future should consider expanding the scope of their work by formally conducting an economic evaluation. Early and sustained collaboration with health economists can help these investigators to collect relevant economic information and ensure findings are presented in a manner that supports their integration into the broader health economic literature and can inform implementation decisions.

Further research is also needed to improve the representation of certain regions and clinical applications in the evidence base. We identified minimal health economic evidence about the use of WES/WGS for screening or in regions such as South America, Africa or Asia; in the latter case, it is not clear whether this is because relevant studies do not exist or because they are not published in English. Many studies were limited by the fact that country- and context-specific willingness-to-pay thresholds were not provided. In particular, many CEAs could not draw cost-effectiveness conclusions because a willingness-to-pay per additional diagnosis was not available for a specific disease/country context or was not reported by the study; some claimed that more costly but more expensive interventions were ‘cost-effective’ without reference to a willingness-to-pay threshold.

In some cases, economic evidence for specific clinical applications of WES/WGS is geographically concentrated in particular regions. For instance, half of the included pathogen WGS studies were set in Australia and nearly half of the precision oncology studies were set in the Netherlands. This could enable evidence synthesis to refine estimates within a region and clinical application. However, making definitive inferences about cost-effectiveness in one context based on evidence collected in a different context or at a different point in time may be inappropriate and should be approached carefully. One reason for this is that the costs of sequencing and corresponding changes in care, including diagnostic investigations averted or treatment decisions informed, can vary between regions and over time. In addition, the cost-effectiveness of sequencing that leads to changes in care is highly sensitive to other decisions that impact the care options available. For example, the value of using WGS for precision oncology will be highly dependent on the availability, cost and value of anti-cancer therapies; if the test results suggest switching to a therapy that is not available in that healthcare system, the test’s utility is diminished. The cost-effectiveness of the recommended therapy will also affect the cost-effectiveness of sequencing—if test results lead to treatment switching from standard care to an alternative that is not incrementally cost-effective, it is unlikely that sequencing itself would be cost-effective. Sensitivity and scenario analyses can be useful tools for translating cost-effectiveness results to other contexts where the values of key cost and outcome input parameters are likely to differ. These analyses could improve the translation of results across diverse contexts and support adaptation to evolving model inputs over time (e.g. sequencing costs decrease or downstream care changes over time).

Certain considerations that could affect decision-making in theory were absent from economic evaluations of WES/WGS. For example, if the incremental cost of WES/WGS is expected to decline, there may be a rationale for delaying adoption—even if the intervention is currently cost-effective—because the net benefit of sequencing may be greater if performed in the future. However, this would only be the case if the decrease in incremental cost from waiting outweighs the clinical benefits of obtaining genomic findings sooner. The value of delaying sequencing implementation is likely to be highly context-specific and will depend on the clinical application and anticipated cost trajectory of WES/WGS and relevant comparators. Separately, there is potential value in reusing and/or reanalysing genomic information to inform future healthcare decisions for related or unrelated diseases, as well as for medical research. The former value is only realised if re-analysis reuses genomic data rather than repeating WES/WGS, which might not reflect existing practice. Furthermore, implementing WES/WGS and generating evidence of its effectiveness not only provides valuable information for decision-making, but it can also intrinsically improve the effectiveness of the intervention itself by contributing knowledge about genotype-phenotype associations, if the data are accessible to researchers. We did not identify studies that explicitly estimate these, or other, broader value elements of WES/WGS or incorporate them into economic evaluation, but it is likely that their value is positive [68].

### Limitations of this review

Our review has several limitations. First, due to resource constraints and the large number of studies considered, a comprehensive quality assessment was not performed on each study or review. This may limit the interpretation of the quality of cost and cost-effectiveness conclusions of included studies. Second, although we assessed studies published since 28 July 2016, many of these evaluated costs and cost-effectiveness using data from the early 2010s or earlier. Likewise, the timeframes searched by the reviews we assessed extended in many cases to articles published before these dates—in one study, as early as 1991. The lags between the implementation of WES/WGS and the availability of clinical and economic evidence make it challenging to draw timely conclusions about costs and cost-effectiveness across studies, particularly given the rapidly changing costs of sequencing over time. In addition, evolving clinical pathways, treatment options and downstream benefits of diagnosis mean that the clinical benefits—and, therefore, the cost-effectiveness—of WES/WGS can change from year to year. This highlights the value of undertaking additional, up-to-date research in this space and underscores the value of sensitivity and scenario analysis as a way for researchers to make sense of results in light of subsequent changes in practice.

Although we identified far more economic evaluations than existing reviews in this area, there were limits on the breadth of our search and analyses. For example, economic evaluations of other types of sequencing, such as transcriptome analysis, were not considered. We only considered English-language publications, which may give a distorted view of the geographic focus of cost and cost-effectiveness studies of WES/WGS worldwide. However, an English-language search does cover multiple high-profile genomic programmes located in the US, UK and Canada. We did not search databases of HTAs in English-speaking countries outside of the UK because either HTA reports were indexed in bibliographic journals in the country or WES/WGS did not fall within the remit of the country’s HTA agency.

Last, due to the heterogeneity of studies in terms of technology, time period and geographical context, we did not pool cost-effectiveness estimates. This review is intended to be an overview of health economic work done in this area and a resource to identify and interpret evidence relevant to a specific decision context, rather than to generate a universal estimate of the cost or cost-effectiveness of WES/WGS.

## Conclusions

WES and WGS are relatively recent additions to routine clinical practice and the evidence base on their costs and cost-effectiveness is evolving. We have identified critical gaps that exist in this evidence base, which should motivate new studies in this field. Development of economic evidence for WES/WGS has been—and likely will continue to be—complicated on account of rapidly changing costs, significant uncertainty around future benefits, scale economies and a wide range of distinct applications of the technologies [69]. However, if the costs of sequencing decline and additional insights can be learned by reanalysing sequencing data, the cost-effectiveness of WES/WGS may be improved. It is important that health economic evidence is collected from the time that WES/WGS initiatives are launched so that cost-effectiveness can be assessed and used to inform future decisions about implementing or expanding sequencing programmes.

## Supplementary information

Supplementary materials

## References

[1] van Nimwegen KJ, van Soest RA, Veltman JA, Nelen MR, van der Wilt GJ, Vissers LE, et al. Is the $1000 genome as near as we think? A cost analysis of next-generation sequencing. Clin Chem. 2016;62:1458–64. 10.1373/clinchem.2016.258632 27630156

[2] Wetterstrand KA. DNA Sequencing Costs: Data from the NHGRI Genome Sequencing Program (GSP) www.genome.gov/sequencingcostsdata.

[3] Alarcón Garavito GA, Moniz T, Déom N, Redin F, Pichini A, Vindrola-Padros C. The implementation of large-scale genomic screening or diagnostic programmes: a rapid evidence review. Eur J Hum Genet. 2023;31:282–95. 10.1038/s41431-022-01259-8 PMC9995480 36517584

[4] Frost A 100,000 Genomes Project: NHS England; 2024 https://www.genomicseducation.hee.nhs.uk/genotes/knowledge-hub/100000-genomes-project/.

[5] Lunke S, Eggers S, Wilson M, Patel C, Barnett CP, Pinner J, et al. Feasibility of Ultra-Rapid Exome Sequencing in Critically Ill Infants and Children With Suspected Monogenic Conditions in the Australian Public Health Care System. JAMA. 2020;323:2503–11. 10.1001/jama.2020.7671 PMC7312414 32573669

[6] Holm IA, Agrawal PB, Ceyhan-Birsoy O, Christensen KD, Fayer S, Frankel LA, et al. The BabySeq project: implementing genomic sequencing in newborns. BMC Pediatr. 2018;18:225. 10.1186/s12887-018-1200-1 PMC6038274 29986673

[7] Genomics England. The Generation Study 2025 https://www.genomicsengland.co.uk/initiatives/newborns.

[8] Stark Z, Scott RH. Genomic newborn screening for rare diseases. Nat Rev Genet. 2023;24:755–66. 10.1038/s41576-023-00621-w 37386126

[9] Turnbull C, Firth HV, Wilkie AOM, Newman W, Raymond FL, Tomlinson I, et al. Population screening requires robust evidence - genomics is no exception. Lancet. 2024;403:583–6. 10.1016/S0140-6736(23)02295-X 38070525

[10] Schwarze K, Buchanan J, Taylor JC, Wordsworth S. Are whole-exome and whole-genome sequencing approaches cost-effective? A systematic review of the literature. Genet Med. 2018;20:1122–30. 10.1038/gim.2017.247 29446766

[11] Alam K, Schofield D. Economic evaluation of genomic sequencing in the paediatric population: a critical review. Eur J Hum Genet. 2018;26:1241–7. 10.1038/s41431-018-0175-6 PMC6117303 29795475

[12] Douglas MP, Ladabaum U, Pletcher MJ, Marshall DA, Phillips KA. Economic evidence on identifying clinically actionable findings with whole-genome sequencing: a scoping review. Genet Med. 2016;18:111–6. 10.1038/gim.2015.69 PMC4654986 25996638

[13] Price V, Ngwira LG, Lewis JM, Baker KS, Peacock SJ, Jauneikaite E, et al. A systematic review of economic evaluations of whole-genome sequencing for the surveillance of bacterial pathogens. Micro Genom. 2023;9:02. 10.1099/mgen.0.000947 PMC9997737 36790430

[14] Rezapour A, Souresrafil A, Barzegar M, Sheikhy-Chaman M, Tatarpour P. Economic evaluation of next-generation sequencing techniques in diagnosis of genetic disorders: a systematic review. Clin Genet. 2023;103:513–28. 10.1111/cge.14313 36808726

[15] Freeman K, Dinnes J, Shinkins B, Clark C, Kander I, Scandrett K, et al. Evaluating whole genome sequencing for rare diseases in newborn screening: evidence synthesis from a series of systematic reviews. Preprint at 10.1101/2024.09.03.24312979 2024. PMC12703562 41358591

[16] Page MJ, McKenzie JE, Bossuyt PM, Boutron I, Hoffmann TC, Mulrow CD, et al. The PRISMA 2020 statement: an updated guideline for reporting systematic reviews. BMJ. 2021;372:n71. 10.1136/bmj.n71 PMC8005924 33782057

[17] Bayle A, Droin N, Besse B, Zou Z, Boursin Y, Rissel S. Whole exome sequencing in molecular diagnostics of cancer decreases over time: evidence from a cost analysis in the French setting. Eur J Health Econ. 2021;22:855–64. 10.1007/s10198-021-01293-1 33765190

[18] Tsiplova K, Zur RM, Ungar WJ. A microcosting and cost-consequence analysis of genomic testing strategies in autism spectrum disorder. Canada: Technology Assessment at SickKids (TASK); 2016. 10.1038/gim.2017.47 28471434

[19] Husereau D, Drummond M, Augustovski F, de Bekker-Grob E, Briggs AH, Carswell C, et al. Consolidated Health Economic Evaluation Reporting Standards 2022 (CHEERS 2022) statement: updated reporting guidance for health economic evaluations. MDM Policy Pract. 2022;7:23814683211061097. 10.1177/23814683211061097 PMC8755935 35036563

[20] Hu X, Guo R, Guo J, Qi Z, Li W, Hao C. Parallel tests of whole exome sequencing and copy number variant sequencing increase the diagnosis yields of rare pediatric disorders. Front Genet. 2020;11:473. 10.3389/fgene.2020.00473 PMC7300249 32595695

[21] Katragadda S, Hall TO, Bettadapura R, Dalton JC, Ganapathy A, Ghana P, et al. Determining cost-optimal next-generation sequencing panels for rare disease and pharmacogenomics testing. Clin Chem. 2021;67:1122–32. 10.1093/clinchem/hvab059 34120169

[22] Oberg JA, Glade Bender JL, Sulis ML, Pendrick D, Sireci AN, Hsiao SJ, et al. Implementation of next-generation sequencing into pediatric hematology-oncology practice: moving beyond actionable alterations. Genome Med. 2016;8:133. 10.1186/s13073-016-0389-6 PMC5180407 28007021

[23] Lee XJ, Elliott TM, Harris PNA, Douglas J, Henderson B, Watson C, et al. Clinical and economic outcomes of genome sequencing availability on containing a hospital outbreak of resistant Escherichia coli in Australia. Value Health. 2020;23:994–1002. 10.1016/j.jval.2020.03.006 32828227

[24] Christensen KD, Vassy JL, Phillips KA, Blout CL, Azzariti DR, Lu CY, et al. Short-term costs of integrating whole-genome sequencing into primary care and cardiology settings: a pilot randomized trial. Genet Med. 2018;20:1544–53. 10.1038/gim.2018.35 PMC6151171 29565423

[25] Stark Z, Schofield D, Alam K, Wilson W, Mupfeki N, Macciocca I, et al. Prospective comparison of the cost-effectiveness of clinical whole-exome sequencing with that of usual care overwhelmingly supports early use and reimbursement. Genet Med. 2017;19:867–74. 10.1038/gim.2016.221 28125081

[26] Schwarze K, Buchanan J, Fermont JM, Dreau H, Tilley MW, Taylor JM, et al. The complete costs of genome sequencing: a microcosting study in cancer and rare diseases from a single center in the United Kingdom. Genet Med. 2020;22:85–94. 10.1038/s41436-019-0618-7 PMC6944636 31358947

[27] Jegathisawaran J, Tsiplova K, Ungar WJ. A microcosting and cost-consequence analysis of genomic testing strategies (including trios) in autism spectrum disorder: an update. Canada: Technology Assessment at SickKids (TASK); 2019.

[28] Tsiplova K, Zur RM, Marshall CR, Stavropoulos DJ, Pereira SL, Merico D, et al. A microcosting and cost-consequence analysis of clinical genomic testing strategies in autism spectrum disorder. Genet Med. 2017;19:1268–75. 10.1038/gim.2017.47 28471434

[29] Pasmans CTB, Tops BBJ, Steeghs EMP, Coupé VMH, Grünberg K, de Jong EK, et al. Micro-costing diagnostics in oncology: from single-gene testing to whole- genome sequencing. Expert rev. 2021;21:413–4. 10.1080/14737167.2021.1917385 33852815

[30] Abbott M, McKenzie L, Moran BVG, Heidenreich S, Hernandez R, Hocking-Mennie L, et al. Continuing the sequence? Towards an economic evaluation of whole genome sequencing for the diagnosis of rare diseases in Scotland. J. 2022;13:487–501. 10.1007/s12687-021-00541-4 PMC9530076 34415556

[31] Jegathisawaran J, Tsiplova K, Hayeems RZ, Marshall CR, Stavropoulos DJ, Pereira SL, et al. Trio genome sequencing for developmental delay and pediatric heart conditions: a comparative microcost analysis. Genet Med. 2022;24:1027–36. 10.1016/j.gim.2022.01.020 35219592

[32] Jegathisawaran J, Tsiplova K, Hayeems R, Ungar W. Microcosting of whole genome sequencing (WGS) of trios in heterogeneous pediatric cardiac population. Canada: Technology Assessment at SickKids (TASK); 2020.

[33] Gordon LG, White NM, Elliott TM, Nones K, Beckhouse AG, Rodriguez-Acevedo AJ, et al. Estimating the costs of genomic sequencing in cancer control. BMC Health Serv Res. 2020;20:492. 10.1186/s12913-020-05318-y PMC7268398 32493298

[34] Mellmann A, Bletz S, Böking T, Kipp F, Becker K, Schultes A, et al. Real-time genome sequencing of resistant bacteria provides precision infection control in an institutional setting. J Clin Microbiol. 2016;54:2874–81. 10.1128/JCM.00790-16 PMC5121374 27558178

[35] Kamboj M, McMillen T, Syed M, Chow Hoi Y, Jani K, Aslam A, et al. Evaluation of a combined multilocus sequence typing and whole-genome sequencing two-step algorithm for routine typing of clostridioides difficile. J Clin Microbiol. 2021;59:10.1128/jcm.01955-20. PMC8111118 33177119

[36] Crawford SA, Gong CL, Yieh L, Randolph LM, Hay JW. Diagnosing newborns with suspected mitochondrial disorders: an economic evaluation comparing early exome sequencing to current typical care. Genet Med. 2021;23:1854–63. 10.1038/s41436-021-01210-0 34040192

[37] Stark Z, Schofield D, Martyn M, Rynehart L, Shrestha R, Alam K, et al. Does genomic sequencing early in the diagnostic trajectory make a difference? A follow-up study of clinical outcomes and cost-effectiveness. Genet Med. 2019;21:173–80. 10.1038/s41436-018-0006-8 29765138

[38] Kingsmore SF, Smith LD, Kunard CM, Bainbridge M, Batalov S, Benson W, et al. A genome sequencing system for universal newborn screening, diagnosis, and precision medicine for severe genetic diseases. Am J Hum Genet. 2022;109:1605–19. 10.1016/j.ajhg.2022.08.003 PMC9502059 36007526

[39] Newton S, Allani P, Salinger K, Parsons J, Vogan A. MSAC application no. 1646: Whole genome sequencing of antimicrobial-resistant pathogens. Australia: Adelaide Health Technology Assessment (AHTA); 2021.

[40] Joshi C, Kolbe DL, Mansilla MA, Mason SO, Smith RJ, Campbell CA. Reducing the cost of the diagnostic odyssey in early onset epileptic encephalopathies. Biomed Res Int. 2016;2016:6421039. 10.1155/2016/6421039 PMC4875968 27243033

[41] Olde Keizer R, Marouane A, Deden AC, van Zelst-Stams WAG, de Boode WP, Keusters WR, et al. Medical costs of children admitted to the neonatal intensive care unit: The role and possible economic impact of WES in early diagnosis. Eur J Med Genet. 2022;65:104467. 10.1016/j.ejmg.2022.104467 35240323

[42] Dymond A, Davies H, Mealing S, Pollit V, Coll F, Brown NM, et al. Genomic surveillance of methicillin-resistant Staphylococcus aureus: a mathematical early modeling study of cost-effectiveness. Clin Infect Dis. 2020;70:1613–9. 10.1093/cid/ciz480 PMC7145999 31219153

[43] Gordon LG, Elliott TM, Forde B, Mitchell B, Russo PL, Paterson DL, et al. Budget impact analysis of routinely using whole-genomic sequencing of six multidrug-resistant bacterial pathogens in Queensland, Australia. BMJ Open. 2021;11:e041968. 10.1136/bmjopen-2020-041968 PMC7852923 33526501

[44] Middelburg P, Monroe G, Van Gassen K, Hovels A, Knoers N, Vrijenhoek T, et al. Impact of whole exome sequencing (WES) on costs and medical decision-making. Value Health. 2016;19:A705–A6.

[45] Cirillo L, Becherucci F, Bellelli S, Mazzinghi B, Raglianti V, Lugli G, et al. Cost-analysis of a clinical workflow for diagnosis of inherited kidney diseases. Nephrol Dialysis Transplant. 2022;37:i774.

[46] Marshall DA, Degeling K, Tagimacruz T, Seeger TA, Boycott KM, Bernier F, et al. Costs and effectiveness of whole exome sequencing (WES) in patients with unsolved rare disease through the diagnostic pathway. Int J Technol Assess Health Care. 2023;39:S50.

[47] Córdoba M, Rodríguez-Quiroga SA, Vega PA, Salinas V, Pérez-Maturo J, Amartino H, et al. Whole exome sequencing in neurogenetic odysseys: an effective, cost- and time-saving diagnostic approach. PLoS ONE. 2018;13:e0191228. 10.1371/journal.pone.0191228 PMC5794057 29389947

[48] Masri A, Hamamy H. Cost effectiveness of whole exome sequencing for children with developmental delay in a developing country: a study from Jordan. J Pediatr Neurol. 2022;20:20–3.

[49] Monies D, Goljan E, Binmanee AM, Alashwal AAZ, Alsonbul AM, Alhussaini AA, et al. The clinical utility of rapid exome sequencing in a consanguineous population. Genome Med. 2023;15:44. 10.1186/s13073-023-01192-5 PMC10283251 37344829

[50] Montaut S, Tranchant C, Drouot N, Rudolf G, Guissart C, Tarabeux J, et al. Assessment of a targeted gene panel for identification of genes associated with movement disorders. JAMA Neurol. 2018;75:1234–45. 10.1001/jamaneurol.2018.1478 PMC6233854 29913018

[51] Zhai Y, Zhang Z, Shi P, Martin DM, Kong X. Incorporation of exome-based CNV analysis makes trio-WES a more powerful tool for clinical diagnosis in neurodevelopmental disorders: a retrospective study. Hum Mutat. 2021;42:990–1004. 10.1002/humu.24222 34015165

[52] Ding Z. Cost-effectiveness analysis of a whole genome sequencing test compared to the standard care among patients with hospital-acquired bacteremia. Unpublished: University of Pittsburgh; 2017.

[53] Tal-Ben Ishay R, Shil A, Solomon S, Sadigurschi N, Abu-Kaf H, Meiri G, et al. Diagnostic yield and economic implications of whole-exome sequencing for ASD diagnosis in Israel. Genes. 2021;13:23. 10.3390/genes13010036 PMC8774872 35052376

[54] Sainio MT, Aaltio J, Hyttinen V, Kortelainen M, Ojanen S, Paetau A, et al. Effectiveness of clinical exome sequencing in adult patients with difficult-to-diagnose neurological disorders. Acta Neurolog Scandin. 2022;145:63–72. 10.1111/ane.13522 34418069

[55] Smith HS, Swint JM, Lalani SR, de Oliveira Otto MC, Yamal J-M, Russell HV, et al. Exome sequencing compared with standard genetic tests for critically ill infants with suspected genetic conditions. Genet Med. 2020;22:1303–10. 10.1038/s41436-020-0798-1 32336750

[56] Dragojlovic N, Elliott AM, Adam S, van Karnebeek C, Lehman A, Mwenifumbo JC, et al. The cost and diagnostic yield of exome sequencing for children with suspected genetic disorders: a benchmarking study. Genet Med. 2018;20:1–9. 10.1038/gim.2017.226 29300375

[57] Strauss KA, Gonzaga-Jauregui C, Brigatti KW, Williams KB, King AK, Van Hout C, et al. Genomic diagnostics within a medically underserved population: efficacy and implications. Genet Med. 2018;20:31–41. 10.1038/gim.2017.76 28726809

[58] Cheng X, Chen J, Yang X, Chan H, Yang X, Jiao J, et al. Comparison of different genetic testing modalities applied in paediatric patients with steroid-resistant nephrotic syndrome. Ital. 2024;50:85. 10.1186/s13052-024-01655-4 PMC11040871 38654395

[59] Nurchis MC, Radio FC, Salmasi L, Heidar Alizadeh A, Raspolini GM, Altamura G, et al. Bayesian cost-effectiveness analysis of whole-genome sequencing versus whole-exome sequencing in a pediatric population with suspected genetic disorders. Eur J Health Econ. 2023;17:17. 10.1007/s10198-023-01644-0 PMC11283423 37975990

[60] Nurchis MC, Radio FC, Salmasi L, Heidar Alizadeh A, Raspolini GM, Altamura G, et al. Cost-effectiveness of whole-genome vs whole-exome sequencing among children with suspected genetic disorders. JAMA Netw. 2024;7:e2353514. 10.1001/jamanetworkopen.2023.53514 PMC10818217 38277144

[61] Santos Gonzalez F, Mordaunt D, Stark Z, Dalziel K, Christodoulou J, Goranitis I. Microcosting diagnostic genomic sequencing: a systematic review. Genet Med. 2023;25:100829. 10.1016/j.gim.2023.100829 36939042

[62] Norris S, Belcher A, Howard K, Ward RL. Evaluating genetic and genomic tests for heritable conditions in Australia: lessons learned from health technology assessments. J. 2022;13:503–22. 10.1007/s12687-021-00551-2 PMC9530105 34570356

[63] Weymann D, Dragojlovic N, Pollard S, Regier DA. Allocating healthcare resources to genomic testing in Canada: latest evidence and current challenges. J. 2022;13:467–76. 10.1007/s12687-019-00428-5 PMC9530078 31273679

[64] Douglas MP, Deverka PA, Gelb B, Ferket B, Lich KH, Smith HS, et al. Report–cost and clinical utility of WES and WGS in pediatric patients with suspected genetic disease. Preprint at 10.1101/2022.10.10.22280925 2022.

[65] Young C, Argáez C. Rapid Genome-wide Testing: A Review of Clinical Utility, Cost-Effectiveness, and Guidelines [CADTH Rapid Response Report: Summary with Critical Appraisal] [Internet]. Ottowa: CADTH; 2019. Available from: https://www.ncbi.nlm.nih.gov/books/NBK549546/pdf/Bookshelf_NBK549546.pdf. 31721549

[66] Tran M, Smurthwaite KS, Nghiem S, Cribb DM, Zahedi A, Ferdinand AD, et al. Economic evaluations of whole-genome sequencing for pathogen identification in public health surveillance and health-care-associated infections: a systematic review. Lancet Microbe. 2023;4:e953–e62. 10.1016/S2666-5247(23)00180-5 37683688

[67] Fontes Marx M, Ataguba JE, de Vries J, Wonkam A. Systematic review of the economic evaluation of returning incidental findings in genomic research. Front Public Health. 2021;9:697381. 10.3389/fpubh.2021.697381 PMC8281014 34277554

[68] Campbell D, Spencer S, Kang A, Pandey R, Katsandres S, Veenstra D. A value framework for evaluating population genomic programs: a mixed methods approach. J. 2025;15:307. 10.3390/jpm15070307 PMC12296045 40710424

[69] Phillips KA, Deverka PA, Marshall DA, Wordsworth S, Regier DA, Christensen KD, et al. Methodological issues in assessing the economic value of next-generation sequencing tests: many challenges and not enough solutions. Value Health. 2018;21:1033–42. 10.1016/j.jval.2018.06.017 PMC6159915 30224106

