# Supplementary material for: The cost and cost-effectiveness of whole-exome and whole-genome sequencing: a systematic literature review
Source: Eur J Hum Genet. 2026 Jun 1;34(11):1602–10. doi: 10.1038/s41431-026-02146-2 (PMC13743555; doi:10.1038/s41431-026-02146-2)
Supplement: Supplementary file 1 [file 41431_2026_2146_MOESM1_ESM.pdf]

Supplementary Appendix 1:

MEDLINE:

1.	whole genome sequenc*.ti,ab.
2.	wgs.ti,ab.
3.	whole exome sequenc*.ti,ab.
4.	wes.ti,ab.
5.	genomic sequenc*.ti,ab.
6.	1 or 2 or 3 or 4 or 5
7.	economic evaluation.ti,ab.
8.	cost benefit.ti,ab.
9.	cost utilit*.ti,ab.
10.	cost minimis*.ti,ab.
11.	cost consequence.ti,ab.
12.	cost effective*.ti,ab.
13.	resource allocation*.ti,ab.
14.	health technology assessment.ti,ab.
15.	7 or 8 or 9 or 10 or 11 or 12 or 13 or 14
16.	6 and 15
17.	(editorial or letter or note).pt. or (rat or rats or mouse or mice or hamster or hamsters or animal or animals or dog or dogs or cat or cats or bovine or sheep or primate or primates).ti,ab,mp.
18.	16 not 17
19.	limit 18 to english
20.	limit 19 to dt=20160728-20240507
21.	limit 19 to rd=20160728-20240507
22.	20 or 21

EMBASE:

1.	whole genome sequenc*.ti,ab.
2.	wgs.ti,ab.
3.	whole exome sequenc*.ti,ab.
4.	wes.ti,ab.
5.	genomic sequenc*.ti,ab.
6.	1 or 2 or 3 or 4 or 5
7.	economic evaluation.ti,ab.
8.	cost benefit.ti,ab.
9.	cost utilit*.ti,ab.
10.	cost minimis*.ti,ab.
11.	cost consequence.ti,ab.
12.	cost effective*.ti,ab.
13.	resource allocation*.ti,ab.
14.	health technology assessment.ti,ab.
15.	7 or 8 or 9 or 10 or 11 or 12 or 13 or 14
16.	6 and 15
17.	(editorial or letter or note).pt. or (rat or rats or mouse or mice or hamster or hamsters or animal or animals or dog or dogs or cat or cats or bovine or sheep or primate or primates).ti,ab,mp.
18.	16 not 17
19.	limit 18 to english
20.	limit 19 to dd=20160728-20240502
21.	limit 19 to rd=20160728-20240502
22.	20 or 21

PubMed

(("resource*allocation") OR ("economic evaluation") OR ("cost*utility") OR ("cost*benefit") OR ("health technology assessment") OR ("cost*consequence") OR ("cost*effectiveness") OR ("cost effectiveness"))
AND
(("whole*genome sequenc*") OR ("WGS") OR ("whole*exome sequenc*") OR ("WES") OR ("genomic sequenc*"))

Tufts CEA registry:

Results for the search: “whole genome” OR “whole exome”

International HTA Database:

(genome) OR (exome) FROM 2016 TO 2024

Restricted to English language entries.

National Institute for Health and Care Excellence (NICE):

Highly specialized technologies guidance, medical technologies guidance, or technology appraisal guidance last updated between 28/7/2016 and 2/5/2024 with the keywords ‘gene’ or ‘genomic’

‘Gene’ search: link

‘Genome’ search: link

‘Genomic’ search: link

‘Exome’ search: link

‘Exomic’ search: link

Scottish Medicines Consortium (SMC):

The ‘Detailed advise’ of all published medicines advice between 28/7/2016-2/5/2024

‘genome’ search: link

‘genomic’ search: link

‘exome’ search: link

‘exomic’ search: link

EconLit

((cost effective*) OR (cost-effective*) OR (economic evaluation) OR (health technology assessment) OR (HTA) OR (cost benefit analysis) OR (cost utility) OR (cost consequence) OR (resource allocation))

AND

((whole exome sequenc*) OR (whole genome sequenc*) OR (WGS) OR (WES) OR (next generation sequenc*) or (genomic sequenc*) OR (pharmacogenomic*) OR (pharmacogenetic*))

Supplementary Appendix 2: Population-Intervention-Comparison-Outcome-Study Design Inclusion Criteria

**PICOS element**	**Description of Inclusion Criterion**
Population	Humans undergoing WES or WGS to inform patient-level health care decisions<br><br>Infectious disease pathogen (e.g., bacteria) whole-genome sequencing to inform patient level health care decisions
Intervention	Whole-genome sequencing; whole-exome sequencing†
Comparison	Other sequencing methods<br><br>Other diagnostic methods or pathways<br><br>No comparator
Outcome	Required: Costs<br><br>If applicable: Clinically relevant outcome measures (diagnostic yield/successful diagnosis, time to diagnosis, change in treatment, survival rate, QALYs) or estimated cost-effectiveness (cost per QALY, cost per diagnosis, cost per death averted)
Study designs	Health technology assessments, systematic reviews, meta-analyses, economic evaluations, evidence-based guidelines, conference abstracts, clinical trials*, pre-prints

†Studies that conducted whole-genome or whole-exome sequencing, and then analysed only a region of the sequenced exome/genome, were still included because the costs incurred are comparable to a full WES/WGS work-up.

*Study protocols were not included if results had not yet been published at the time of review.

Table modified from Schwarze et al. (2018) [1].

Abbreviations: QALY: quality-adjusted life year; WES: whole exome sequencing; WGS: whole genome sequencing

Supplementary Appendix 3: Definitions of Clinical Applications

Clinical Application	Definition
Prenatal screening	Human WES/WGS was used to detect genetic disorders in a foetus in utero.
Newborn screening	Human WES/WGS was used to detect genetic disorders in newborns for conditions that may not be apparent at birth.
Healthy population screening	Human WES/WGS was used to identify genetic predispositions in healthy individuals (children or adults) not suspected of a specific disease
Diagnostic tool	Human WES/WGS was used to diagnose diseases with a suspected genetic cause.*
Precision oncology	Human WES/WGS was used to identify genetic mutations that influence how a patient responds to specific medications in germline and/or somatic cells, which can affect the selection of anti-cancer therapies in a particular patient*
Pathogenic screening	WGS of pathogens such as bacteria was performed to determine potential resistance to antibiotics and customise treatment to people infected with resistant infections.

Abbreviations: WES: whole-exome sequencing; WGS: whole-genome sequencing
*Any cases in which sequencing was used for familial diagnosis of a cancer predisposition syndrome were classified under “diagnostic tool”, not precision oncology.

Supplementary Appendix 4: Methods for currency indexing and conversion

Costs were reported by year and in their original currency, then converted to 2024 Great British Pounds (GBP) and US Dollars (USD) using a publicly available tool that applies purchasing power parity (PPP) estimates from the International Monetary Fund (IMF) [2].

As a robustness check, we also performed a pure exchange rate conversion of costs into 2024 GBP and USD using published exchange rates and inflation indices that do not adjust for purchasing power parity. This was performed by first converting the estimate into GBP and USD for the index year using yearly average exchange rates to GBP and USD published on ofx.com [3]. Those values were then inflated to 2024 price levels: for GBP, using the UK Office of National Statistics consumer price index for health [4]; and, for USD, using the Consumer Price Index for Medical Care published by the US Bureau of Labor Statistics [5].

Supplementary Appendix 5:

Summary table 5A: Included primary research studies that are full economic evaluations

Paper Name	Paper Authors	Publication Year	Type of article	Country Name	Disease context	Sequencing methods	Type of economic evaluation	Comparator †	Currency year	Currency	Sequencing Platform	Summary and cost-effectiveness results	Reference
Cost-Effectiveness of Exome Sequencing versus Targeted Gene Panels for Prenatal Diagnosis of Fetal Effusions and Non-Immune Hydrops Fetalis	Avram et al.	2022	Original Research	USA	Prenatal Screening	Human WES	CUA	Gene panels	2022	USD	not specified	The study demonstrates that WES is a cost-effective strategy for the prenatal diagnosis of nonimmune hydrops fetalis (NIHF) and fetal effusions compared to targeted gene panels and no additional testing. WES was the dominant strategy against all of the other nine strategies at <18 weeks and 18-22 weeks of gestation. WES was the dominant strategy against eight of nine strategies at >22 weeks. The non-dominated strategy was no additional testing. The ICER for WES vs no additional testing was $7,365****; thus, WES was cost-effective at the stated WTP threshold of $100,000.<br>At this WTP threshold, WES "*was cost-effective up to a cost per test of $50,451 at <18 weeks of gestation, $50,423 at 18 to 22 weeks of gestation, and $9,530 at >22 weeks of gestation (Figure 2). In a Monte Carlo analysis at <18, 18 to 22, and >22 weeks of gestation, [WES] alone was the cost-effective strategy in 98%..., 98%, and 100% of trials, respectively*".	[6]
Diagnosing newborns with suspected mitochondrial disorders: an economic evaluation comparing early exome sequencing to current typical care.	Crawford, Gong et al.	2021	Full-length research article	USA	Diagnostic tool	Human WES	CUA	Typical care	2019	US dollars	not specified	This study evaluates the economic impact of early WES (eWES) for diagnosing newborns with suspected severe mitochondrial disorders (MitD). Singleton and trio-eWES both dominate singleton typical care. Singleton-eWES had an estimated iNMB of $42,802 at a WTP of $50,000/QALY, and $45,387 at a WTP of $200,000/QALY, relative to singleton typical care. Trio-eWES had an estimated iNMB of $48,432 at a WTP of $50,000/QALY, and $49,826 at a WTP of $200,000/QALY, relative to singleton typical care. In the probabilistic analysis, trio eWES had the highest probability of being the most cost-effective strategy for WTP thresholds under $450,000.	[7]
Cost-effectiveness analysis of a whole genome sequencing test compared to the standard care among patients with hospital-acquired bacteremia	Ding et al.	2017	MSc Thesis	USA	Pathogenic screening	Pathogen WGS	CUA	Standard of care	Not specified	not specified by implied that it is US dollars	not specified	This thesis contains a CUA of WGS compared to the SOC for managing hospital acquired infections with *Klebsiella pneumoniae* carbapenemase-producing bacteria. In the deterministic analysis, WGS dominated the SOC. The ICER was reported as $4,173.36, although it was in fact -$4,173.36 based on the information provided about costs and QALYs. In the probabilistic analysis, the median ICER was -$4,985.91/QALY with WGS in the south-east quadrant (dominant) relative to the SOC. WGS was dominant in 89.6% of simulations. There was an ~0%	[8]

Paper Name	Paper Authors	Publication Year	Type of article	Country Name	Disease context	Sequencing methods	Type of economic evaluation	Comparator †	Currency year	Currency	Sequencing Platform	Summary and cost-effectiveness results	Reference
												probability that WGS was not cost-effective versus the SOC at a WTP of $50,000/QALY.	
Genomic Surveillance of Methicillin-resistant Staphylococcus aureus: A Mathematical Early Modeling Study of Cost-effectiveness	Dymond et al.	2020	Research article	UK (England)	Pathogenic screening	Pathogen WGS	CUA	Existing standard practice	2017/18	British Pounds	not specified	This is a simulation study that performs CUA of routine pathogen WGS for methicillin-resistant *Staphylococcus aureus* (MRSA) control in UK hospitals. WGS was cost-saving and more effective (dominant) compared to existing practice. Introducing pathogen WGS saved an estimated £728,297 per annual hospitalised cohort (a reduction of 31%).	[9]
Cost-effectiveness analysis of whole-genome sequencing during an outbreak of carbapenem-resistant *Acinetobacter baumannii*	Elliott et al.	2021	Research Article	Australia	Pathogenic screening	Pathogen WGS	CUA	No WGS, metagenomics and environmental swabbing	2020	Australian dollars	not specified	This is a simulation study that performs CUA of pathogen WGS for controlling a hospital outbreak of carbapenem-resistant *Acinetobacter baumannii* (CRAB). Both versions of implementing WGS were cost-saving and improved outcomes relative to the scenario without WGS. "*The model was calibrated to the actual pathogen spread within the intensive care unit and burn unit (scenario 1) and compared with early use of WGS (scenario 2) and early use of WGS and metagenomics (scenario 3) to determine their respective cost-effectiveness*." Scenario 2 (WGS alone) was dominant against Scenario 1 (no WGS). Scenario 3 (WGS + metagenomics) was dominant against both Scenario 1 and Scenario 2.	[10]
Cost-effectiveness of exome and genome sequencing for children with rare and undiagnosed conditions	Lavelle et al.	2022	Original research article	USA	Diagnostic tool	Human WGS & WES	CUA	Standard of care	2019	US dollars	not specified	This was a modelling study which estimated the cost-effectiveness of WES and WGS as diagnostic tools for children with suspected genetic conditions. Seven diagnostic strategies were modelled, including SOC. WGS alone was more effective and costly than the SOC, with an incremental cost per diagnosis of $15,048 in infants with severe disability and $27,349 in children with moderate disability. All other strategies were strictly or extendedly dominated. When all strategies which involve WGS are removed as options, WES alone was the next most cost-effective strategy compared with the SOC.	[11]
Application No. 1476. Genetic testing for childhood syndromes	Medical Services Advisory Committee	2019	HTA	Australia	Diagnostic tool	Human WES	CUA	Standard of care	Not specified	Australian dollars	not specified	The study assessed the cost-effectiveness of WES to diagnose children with symptoms indicating a syndromic disorder including at least two of the following indications: multiple congenital anomalies; dysmorphic facial features; and/or moderate to profound cognitive impairment. The base case ICER of $7,254.62/QALY for WES vs SOC.	[12]
Exploring the Cost Effectiveness of a Whole-Genome Sequencing-Based Biomarker for Treatment Selection in Patients with Advanced Lung Cancer Ineligible for Targeted Therapy	Mfumbilwa et al.	2024	Original research article	Europe (Netherlands)	Precision oncology	Human WGS	CUA	Current practice (PD-L1)	2022	Euros	none specified	This study uses a microsimulation model to assesses the cost-utility of several strategies for treatment selection in patients with advanced lung cancer, including (or not including) WGS to evaluate tumor mutational burden. In the base case, the SOC (PD-L1 without WGS) had the highest NMB (€27,300 per patient), estimated at a WTP of €80,000/QALY. This was followed by a WGS tumor mutational burden strategy (NMB: €26,700). The ICER for strategy A (PD-L1 alone) compared to strategy B (WGS-TMB	[13]

Paper Name	Paper Authors	Publication Year	Type of article	Country Name	Disease context	Sequencing methods	Type of economic evaluation	Comparator †	Currency year	Currency	Sequencing Platform	Summary and cost-effectiveness results	Reference
												alone) was €74,900 per QALY. Therefore, using WGS to assess tumor mutational burden was not the most cost-effective strategy at the stated WTP threshold of €80,000 in the base case. However, strategy C (using PD-L1 and WGS-based tumor mutational burden) was cost-effective when used to withhold immunotherapy in patients with low expected benefit.	
Using molecular testing and whole-genome sequencing for tuberculosis diagnosis in a low-burden setting: a cost-effectiveness analysis using transmission-dynamic modelling	Mugwagwa et al.	2021	Original research article	UK (England & Wales)	Pathogenic screening	Pathogen WGS	CUA	Culture-based DST	2014-2015	British Pounds	not specified	The study evaluated the cost-effectiveness of using molecular testing and WGS for tuberculosis diagnosis in England and Wales. The combination of Xpert-Ultra and WGS provided the most cost-effective approach, with an iNMB of £16.5 million over 10 years. iNMB for strategies involving WGS (i.e., WGS alone or WGS in combination with the molecular testing options Xpert or Xpert-Ultra) compared to the baseline clinical pathway ranged from £7.2 million to £16.6 million. iNMB for strategies which did not use WGS (molecular testing only) ranged from £8.7 million to £11.2 million compared to the baseline clinical pathway.	[14]
Cost Efficacy of Rapid Whole Genome Sequencing in the Pediatric Intensive Care Unit	Sanford Kobayashi et al.	2021	Original research article	USA	Diagnostic tool	Human WGS	CUA	Counterfactual trajectories that were confirmed or refuted by an independent Delphi panel.	Not specified	US dollars	not specified	This study evaluates the cost-effectiveness of rWGS in critically ill children in the paediatric intensive care unit. The ICER of rWGS compared to the counterfactual with no rWGS was estimated to be $4,509 per QALY gained.	[15]
Long-term economic impacts of exome sequencing for suspected monogenic disorders: diagnosis, management, and reproductive outcomes	Schofield et al.	2019	Research Article	Australia	Diagnostic tool	Human WES	CUA	Standard diagnostic pathway	Not specified, but the 2018 price list is cited	Australian dollars	not specified	This study was a CUA of WES versus usual diagnostic care for diagnosing suspected monogenic disorders in infants. WES became increasingly cost-effective when considering effects on first-degree relatives and parental reproductive outcomes. When considering probands only, the ICER of WES compared to usual diagnostic care was AU$31,144.35 per QALY gained; when considering probands and first-degree relatives the ICER of WES compared to usual diagnostic care was AU$20,839.57 per QALY gained; when considering probands, first degree relatives, and parental reproductive outcomes, the ICER of WES compared to usual diagnostic care was AU$14,235.28 per QALY gained.	[16]
Early Cost Effectiveness of Whole-Genome Sequencing as a Clinical Diagnostic Test for Patients with Inoperable Stage IIIB,C/IV Non-squamous Non-small-Cell Lung Cancer	Simons, Retel et al.	2021	Original research article	Europe (Netherlands)	Precision oncology	Human WGS	CUA	Standard of care	2020	Euros	NovaSeq 6000	This was a CUA that compared WGS to standard diagnostic testing for patients with inoperable stage IIIB/CIV non squamous non-small-cell lung cancer. WGS is not cost-effective at its current cost at conventionally acceptable WTP thresholds; the ICER for WGS as a diagnostic test vs SOC is €657,571/QALY.	[17]

Paper Name	Paper Authors	Publication Year	Type of article	Country Name	Disease context	Sequencing methods	Type of economic evaluation	Comparator †	Currency year	Currency	Sequencing Platform	Summary and cost-effectiveness results	Reference
Cost-Effectiveness and Budget Impact of Future Developments With Whole-Genome Sequencing for Patients With Lung Cancer	Simons, Uyl-de Groot et al.	2023	Original research article	Europe (Netherlands)	Precision oncology	Human WGS	CUA	Standard of care	2020	Euros	NovaSeq 6000	This was a CUA of WGS for use in precision oncology. The study found that WGS was not cost-effective at the time, but was forecast to be cost-effective in strategies likely to materialise within 5 years: using WGS results for treatment selection; using a WGS-based biomarker for immunotherapy; and, off-label drug approval for WGS results. Using WGS as a diagnostic tool at the time has a base case ICER vs SOC of €636,582/QALY, and an iNMB of –€1,336 per patient. However, the three scenarios likely to occur within five years, and combinations of these strategies, were all dominant versus SOC: the strategy with the highest iNMB – an unweighted combination of all three strategies – had an iNMB of €9,815 per patient.	[18]
Does genomic sequencing early in the diagnostic trajectory make a difference? A follow-up study of clinical outcomes and cost-effectiveness	Stark, Schofield et al.	2019	Original research article	Australia	Diagnostic tool	Human WES	CUA	Standard diagnostic testing	not specified	Australian dollars	not specified	This follow-up study investigated the long-term clinical and economic impacts of reanalysis of WES results. The first group of findings are the results of a CEA of reanalysis at different time points. For Model 1 (reanalysis at 18-months), the incremental cost per additional diagnosis was AU$1,058.74 (95% CI: -10,501.70 to 1,937.17) compared to the standard of care. For Model 2 (reanalysis every 6 months for 18 months), the incremental cost per additional diagnosis was AU$3,578.77 (95% CI: -$231.71 to $17,003.02). The authors concluded that Model 1 was more cost-effective than Model 2. As analysed and presented, Model 1 dominates Model 2 because it leads to the same number of diagnoses over 18 months at lower cost. We note that this conclusion is incomplete in the context of other results reported in the study, namely that Model 1 reduces time-to-diagnosis in some patients who were diagnosed due to WES re-analysis. Thus, the benefits of model 1 over model 2 (faster time-to-diagnosis) were not captured in the outcome used to make conclusions about incremental cost-effectiveness (diagnostic yield by 18 months). The second group of findings related to a CUA to evaluate the effect of using WES in cascade testing on family/reproductive outcomes. Using WES as a diagnostic tool caused changes in management that resulted to cost-savings and QALY gains to the patient who underwent WES versus standard diagnostic care: the cost saving per QALY gained was AU$1,577.88 (95% CI: -$205,449.67 to $19,780.25). When changes in management, cascade testing, and reproductive outcomes (patient and 1st-degree relatives) were considered, the ICER was AU$8,118.70 per QALY gained.	[19]
Cost-effectiveness of whole-exome sequencing in progressive	Aaltio et al.	2022	Original research article	Europe (Finland)	Diagnostic tool	Human WES	CEA	Traditional diagnostic examinations	Not standardised	Euros	MiSeq	This study was a CEA of WES for diagnosis of neurological disorders in children. WES was dominant versus routine early-diagnostic care.	[20]

Paper Name	Paper Authors	Publication Year	Type of article	Country Name	Disease context	Sequencing methods	Type of economic evaluation	Comparator †	Currency year	Currency	Sequencing Platform	Summary and cost-effectiveness results	Reference
neurological disorders of children													
A Clinical Workflow for Cost-Saving High-Rate Diagnosis of Genetic Kidney Diseases	Becherucci et al.	2023	Research Article	Europe (Italy)	Diagnostic tool	Human WES	CEA	Strategies with different timings of WES	2022	Euros	NextSeq 500/550	This study evaluated the cost-effectiveness of an early WES diagnostic workflow for genetic kidney diseases. The first analysis compared two diagnostic strategies. In the first model ("late WES"), tier 1, 2, and 3 investigations were conducted sequentially, with WES reserved for patients who remained undiagnosed at the end of this process. In the second model ("early WES"), WES was implemented immediately after tier 1 investigations. The mean cost per diagnosis was €7,530 in the late WES model and €6,033 in the early WES model, representing an ~20% cost-savings. The second analysis compared two groups. The first group ('real-life cost') reflected the actual costs incurred in a representative subgroup of 66 patients who were randomly selected based on the availability of complete clinical information and administrative data. The second group was a hypothetical scenario in which WES was introduced after tier 1 investigations in this same subgroup. The mean cost per diagnosis was €6,805 in the real-life cost group and €4,009 in the early WES scenario, representing an ~41% cost-savings.	[21]
Cost-effectiveness analysis of using the TBX6-associated congenital scoliosis risk score (TACScore) in genetic diagnosis of congenital scoliosis	Chen, Yan et al.	2020	Research Article	Canada	Diagnostic tool	Human WES	CEA	TACScore (genetic risk score)	2014 (based on fact that conversions to USD were based on 2014 exchange rate)	USD & Chinese Yuan	not specified	This study compares the cost-effectiveness of using the TBX6-associated congenital scoliosis risk score (TACScore) as a pre-screening tool versus WES as a first-line test for diagnosing congenital scoliosis. The average cost for first-line WES was USD$95.4 more than the TACScore strategy from healthcare payer perspective and USD$478.3 more from personal budget perspective. Under both the health care payer and personal budget perspective, using the TACScore strategy was dominant versus WES because it was less costly, had a lower diagnostic time, and led to the same number of diagnoses.	[22]
Comparison of different genetic testing modalities applied in paediatric patients with steroid-resistant nephrotic syndrome	Cheng, Chen et al.	2024	Research Article	China	Diagnostic tool	Human WGS & WES	CEA	Different diagnostic trajectories (early vs late WES/WGS)	Not specified	USD	DNBSeq	This study compared the cost and diagnostic efficiency of different strategies including gene panels, WES, and WGS, for children with steroid-resistant nephrotic syndrome (SRNS). Multiple strategies were tested that placed WES or WGS at different stages of the diagnostic pathway. This paper does not follow best practice guidelines for reporting the results of an economic evaluation and we were not able to replicate or interpret the economic results from the information provided.	[23]
The clinical utility and costs of whole-genome sequencing to detect cancer susceptibility variants – a multi-site	Davidson et al.	2023	Original Research	Australia	Diagnostic tool	Human WGS	CEA	Standard testing (incl. gene test or gene panel)	2020	Australian dollars	HiSeq X Ten	This study assessed WGS to test for cancer susceptibility variants. Three scenarios were modelled: standard practice; WGS after standard testing; and, upfront WGS to replace standard testing. The marginal cost per case with an actionable variant for the approach of standard testing followed by WGS was AU$24,894, compared to standard care alone.	[24]

Paper Name	Paper Authors	Publication Year	Type of article	Country Name	Disease context	Sequencing methods	Type of economic evaluation	Comparator †	Currency year	Currency	Sequencing Platform	Summary and cost-effectiveness results	Reference
prospective cohort study												For upfront WGS, this figure was AU$8,744. Upfront WGS was dominant versus WGS after standard testing because it was less costly and identified more cases with an actionable variant.	
Cost-Effectiveness of Alternative Diagnostic Testing Pathways with Whole Exome Sequencing (WES) in a Rare Disease Patient Population: The Canadian Care-for-Rare SOLVE (SOLVE) Multi-Centre Observational Cohort††††	Degeling et al.	2022	Abstract	Canada (not specified but found cohort from ad-hoc search)	Diagnostic tool	Human WES	CEA	Pathways with different timings of WES or no WES	Not specified	Canadian Dollar	not specified	This was an abstract for a CEA comparing alternative diagnostic pathways – no WES, and WES as a first, second, third, and fourth test – for diagnosis of rare diseases with suspected genetic etiology. The cost per additional diagnosis was estimated to be -$2,762, $491, $5,620, $9,321 respectively for Tiers 1-4 compared to molecular and specialised diagnostic tests (no WES).	[25]
EE171 Time to Diagnosis and Cost Effectiveness of Whole Exome Sequencing (WES) Position in the Diagnostic Pathways of Patients with Suspected Rare Genetic Disease††††	Degeling et al.	2023	Abstract	Not reported	Diagnostic tool	Human WES	CEA	Pathways with different timings of WES or no WES	Not specified	Canadian Dollar	not specified	This was an abstract for a CEA comparing five strategies – no WES, and WES as a first, second, third, and fourth test in the pathway – for diagnosis of rare diseases with a suspected genetic cause. The WES-first strategy dominated all other WES strategies, with a lower cost, lower time-to-diagnosis, and equivalent diagnostic yield. The ICER of WES-first relative to no WES was not reported but can be calculated as CAD$1,506 per additional diagnosis.‡	[26]
Real-World Economic Evaluation of Prospective Rapid Whole-Genome Sequencing Compared to a Matched Retrospective Cohort of Critically Ill Pediatric Patients in the United States	Diaby et al.	2022	Research Article	USA	Diagnostic tool	Human WGS	CEA	Standard diagnostic genetic testing	2020	US dollar	NovaSeq	This study evaluates rWGS for diagnosing critically ill paediatric patients with diseases of unknown cause. rWGS dominated standard testing in the full patient sample (aged 0-18) and a sample restricted to patients aged less than 2 years. In the full sample, rWGS increased the mean diagnostic yield by 26% (13.5% to 39.8%) and decreased mean costs by $100,440 (from $111,469 to $11,029), which resulted in an ICER of -$382,385 per 1% change in the diagnostic yield. In the sample restricted to patients under 2 years of age, rWGS increased the mean diagnostic yield by 17.9% (from 14.6% to 32.5%) and decreased mean costs by $213,472 (from $224,176 to $10,704), which resulted in an ICER of –$1,190,917 per 1% change in the diagnostic yield. In the sensitivity analysis, rWGS dominated standard testing over all positive WTP thresholdrs.	[27, 28]
Exome Sequencing for Isolated Congenital Hearing Loss: A Cost-Effectiveness Analysis	Downie et al.	2020	Original Research	Australia	Diagnostic tool	Human WES	CEA	Standard of care	2020	Australian dollars	not specified	This CEA found that WES was more costly and more effective for diagnosing isolated congenital hearing loss in children. The base case ICER was AU$3,333 per additional diagnosis for the WES pathway relative to the standard care pathway. The study estimated that the mean societal WTP for WES was $4,600 per child, or AU$15,333 per additional diagnosis based on a diagnostic yield of 30%. Thus, WES was cost-effective relative to the standard care pathway and yielded a net benefit of AU$3,600 per patient.	[29]

Paper Name	Paper Authors	Publication Year	Type of article	Country Name	Disease context	Sequencing methods	Type of economic evaluation	Comparator †	Currency year	Currency	Sequencing Platform	Summary and cost-effectiveness results	Reference
The cost and diagnostic yield of exome sequencing for children with suspected genetic disorders: a benchmarking study	Dragojlovic et al.	2018	Full-length research article	Canada	Diagnostic tool	Human WES	CEA	Base case	2016	Canadian dollars (also converted into USD and Euro)	not specified	This study was a CEA of WES for diagnosis of children with suspected genetic disorders. The average cost per patient was $6,138 per patient for Trio-WES with genomic consultation service (GCS), $5,263 per patient for Trio-WES with no GCS, and $5,125 per patient for Singleton WES. In the base case, the cost per positive diagnosis was CAD$14,405 for Trio-WES with GCS, $15,495 for Trio-WES without GCS, and $18,223 for Singleton-WES. The study did not report incremental cost per additional positive diagnosis. We calculate the incremental cost per additional positive diagnosis for each of the following comparisons: 1) Trio-WES without GCS vs Singleton WES: $2,339; 2) Trio-WES with GCS vs Trio-WES without GCS: $10,174; 3) Trio-WES with GCS vs Singleton-WES: $6,986.‡	[30]
Whole exome and genome sequencing in mendelian disorders: a diagnostic and health economic analysis	Ewans & Minoche et al.	2022	Research Article	Australia	Diagnostic tool	Human WGS & WES	CEA	Different strategies involving WES and WGS, including scenario with reanalysis	2020	Australian Dollars (costs also reported in US dollars and GBP)	Illumina HiSeq2500, HiSeq X, Ion Proton, NovaSeq 6000	This study evaluates the comparative effectiveness of WES and WGS for diagnosing individuals with suspected Mendelian disorders. It first analysed WES-reanalysis (using contemporary techniques) and WGS for unresolved cases from a prior WES analysis. For patients who remained undiagnosed after original WES, contemporary WES re-analysis led to seven additional diagnoses at a total cost of AU$15,727. The incremental cost per additional diagnosis of contemporary WES reanalysis followed by WGS compared to contemporary WES alone was AU$36,710. The incremental cost per additional diagnosis of WGS on WES-negative cases compared to WES-reanalysis alone was AU$41,916. The study also analysed using contemporary WES and WGS as initial genomic tests. The incremental cost per additional diagnosis for initial contemporary WES followed by WGS in unresolved cases, compared to initial contemporary WES alone, was AU$36,710. The incremental cost per additional diagnosis for initial WGS compared to initial contemporary WES alone was AU$29,708.	[31]
Whole-exome sequencing reanalysis at 12 months boosts diagnosis and is cost-effective when applied early in Mendelian disorders	Ewans et al.	2018	Research Article	Australia	Diagnostic tool	Human WGS & WES	CEA	Traditional diagnostic pathway; WES/WGS also compared to each other	2016-2017	US Dollar	HiSeq2500	This study evaluated the cost-effectiveness of 12-month reanalysis of WES for diagnosis of Mendelian disorders. Re-analysis of WES increased diagnostic yield from 30% (for initial WES analysis) to 41%. Not including WES re-analysis, the incremental cost per additional diagnosis was $618 for WES at the clinical genetics review and -$586 (cost-saving) at initial symptom, compared to the traditional diagnostic pathway. Including WES re-analysis at 12-months, the incremental cost per additional diagnosis was $726 at clinical genetics review and -$77 (cost-saving) at initial symptom, compared to the traditional pathway. The study did not report incremental outcomes for WES with re-analysis versus WES without re-analysis. WES at initial	[32]

Paper Name	Paper Authors	Publication Year	Type of article	Country Name	Disease context	Sequencing methods	Type of economic evaluation	Comparator †	Currency year	Currency	Sequencing Platform	Summary and cost-effectiveness results	Reference
												symptom without re-analysis and WES at initial symptom with re-analysis are the only two non-dominated strategies. The incremental cost per additional diagnosis of WES at initial symptom with re-analysis compared to WES at initial symptom without re-analysis can be calculated to be $940.‡‡ A hypothetical analysis was then performed between WES and WGS. Assuming a diagnostic yield for WGS of 50%, the incremental cost per additional diagnosis for WGS compared to WES was $11,553.	
A population-based cost-effectiveness study of early genetic testing in severe epilepsies of infancy	Howell et al.	2018	Full-length research article	Australia	Diagnostic tool	Human WES†††	CEA	Seven diagnostic pathways were analysed	2016-17	US Dollars (converted from Australian dollars)	HiSeq 4000	This study analysed the cost-effectiveness of early WES in diagnosing severe epilepsies of infancy. It found that early WES provided more diagnoses at lower costs than traditional diagnostic pathways. Seven diagnostic pathways were evaluated, one of which did not include WES and six of which placed WES as second, third, fourth, or fifth stage tests in the pathway. The average cost per diagnosis in the pathways including WES ranged from $15,378 to $9,904, c.f. $16,951 for the pathway not including WES. The two strategies that were on the cost-effectiveness frontier both placed WES as a second-stage test: "*Pathway 6:* Tier 1 testing → WES → Repeat MRI → Tier 2 testing"; and "*Pathway 7:* Tier 1 testing → WES → Repeat MRI". Pathway 6 is more costly and more effective than Pathway 7. The study only reported ICERs (incremental cost per additional diagnosis) relative to the pathway which did not include WES, but we calculated the ICER of Pathway 6 compared to Pathway 7 to be $48,917 per additional diagnosis.‡‡‡	[33]
Cost-effectiveness of genome sequencing for diagnosing patients with undiagnosed rare genetic diseases	Incerti et al.	2022	Original Research Article	USA	Diagnostic tool	Human WGS	CEA	Standard of care	2020	US dollars	not specified	This study evaluated the cost-effectiveness of WGS for diagnosing paediatric patients with undiagnosed rare genetic diseases. It modelled three strategies: standard diagnostic care alone (no WGS), WGS alone, and WGS following standard care. It estimated ICERs relative to standard care but not incrementally along the cost-effectiveness frontier, and therefore did not identify extendedly dominated options. In addition, the study estimated the ICER as the mean of ICERs across PSA replicates, rather than the standard approach of using the ratio of mean costs and mean benefits across PSA replicates [34]. We report the original ICERs and our calculations for 'standard' ICERs in the table footnote.‡‡‡‡ Results using 'standard' ICERs are as follows: The study first modelled a population of children considering only diagnostic costs. In this context, WGS alone dominated standard care, and the WGS following standard care strategy had an ICER of $24,605 per additional diagnosis. WGS following standard care was more costly and more effective than WGS alone. The ICER for WGS following standard care vs WGS	[35]

Paper Name	Paper Authors	Publication Year	Type of article	Country Name	Disease context	Sequencing methods	Type of economic evaluation	Comparator †	Currency year	Currency	Sequencing Platform	Summary and cost-effectiveness results	Reference
												alone was $474,600. The study then modelled a population of critically ill infants. When only diagnostic costs were considered, WGS alone and WGS following standard care had ICERs of $15,629 and $18,566 per additional diagnosis, respectively, compared to standard care alone. WGS following standard care was extendedly dominated. However, when costs were included from the length of stay in the neonatal intensive care unit, WGS alone strictly dominated both standard care alone and WGS following standard care.	
Cost-Effectiveness of Targeted Exome Analysis as a Diagnostic Test in Glomerular Diseases	Jayasinghe et al.	2021	Original Research Article	Australia	Diagnostic tool	Human WES	CEA	Six diagnostic strategies modeled including 'current practice'	2019	Australian dollars	not specified	This study modelled targeted WES (tWES) as a diagnostic tool for monogenic kidney disease in both adult and paediatric patients. In each age group, it evaluated the cost-effectiveness outcomes of six strategies that included different combinations of tests (tWES, targeted panel tests, and nongenomic investigations), in different orders. In adults, the only non-dominated options were nongenomic investigations only and early tWES. The ICER of early tWES compared to nongenomic investigations only was AU$5,460 per additional diagnosis. tWES was dominant against all other options in children. WES is >95% likely to be cost-effective compared to standard testing at a WTP per additional diagnosis above AU$8,650 in adults, and above AU$950 in children. At the base case WTP threshold of AU$1,400, tWES was likely to be cost-effective in children but not in adults.	[36]
A microcosting and cost-consequence analysis of genomic testing strategies (including trios) in autism spectrum disorder: an update [full report]; Determining accurate costs for genomic sequencing technologies – a necessary prerequisite [journal article]	Jegathisawaran, Tsiplova et al.	2019 [full report]; 2020 [journal article]	Full report [with corresponding short journal article published in 2020]	Canada	Diagnostic tool	Human WGS & WES	CEA‡‡	CMA; different combinations /pathways WES/WGS	2018	Canadian dollars	HiSeq 2500; NextSeq 550; HiSeq X	This study, reported in a full report and journal article, was a microcosting analysis of WES and WGS for autism spectrum disorder in children, compared to CMA. The full report also performed a CEA and was an update to Tsiplova et al., 2017 ([37]). The microcosting estimates were as follows: On a HiSeq 2500, WES cost CAD$1,960 (2024GBP: £1,384) per sample for a proband and CAD$3,143 (£2,220) for a trio. The cost of proband WES was forecast to decline to CAD$1,698 (£1,199) by year 5. On a NextSeq 550, WES cost CAD$1,981 (£1,399) for a proband and CA$4,072.30 (£2,876) for a trio. The cost of proband WES was forecast to decline to CAD$1,747 (£1,233) by year 5. WGS on a HiSeq X cost CAD$3,350 (£2,366) for a proband and CAD$6,556 (£4,629) for a trio. The cost of WGS was forecast to decline to CAD$2,911 (£2,055) per proband and CAD$5,803 (£4,098) per trio by year 5. The full report also presented cost-effectiveness results. Depending on the sequencing platform, incremental costs per additional diagnosis were as follows: CAD$30,154-$30,471 for CMA+WES vs CMA; $31,260-$56,860 for proband/trio-WGS vs CMA; $34,506-$35,810 for proband WGS vs	[38, 39]

Paper Name	Paper Authors	Publication Year	Type of article	Country Name	Disease context	Sequencing methods	Type of economic evaluation	Comparator †	Currency year	Currency	Sequencing Platform	Summary and cost-effectiveness results	Reference
												CMA+WES; and, $104,774-$105,349 for trio-WGS vs CMA+WES.	
Exome Sequencing for Prenatal Detection of Genetic Abnormalities in Fetal Ultrasound Anomalies: An Economic Evaluation	Kodabuckus et al.	2020	Original Research	UK	Prenatal Screening	Human WES	CEA	CMA	2017	GBP, EUR and USD	not specified	The study evaluated the cost-effectiveness of different strategies involving WES and CMA in prenatal diagnosis. In the base case (WES costs GBP£2,100), the incremental cost per additional diagnosis compared to CMA alone is £31,410 for WES alone, £24,657 for stepwise CMA then WES, and £25,581 for CMA+WES. WES alone was extendedly dominated by CMA alone and stepwise CMA then WES, and WES+CMA was dominated by stepwise CMA then WES. The incremental cost per additional diagnosis of stepwise CMA then WES compared to WES alone was £10,307.	[40]
Method for Economic Evaluation of Bacterial Whole Genome Sequencing Surveillance Compared to Standard of Care in Detecting Hospital Outbreaks	Kumar et al.	2021	Original research article	USA	Pathogenic screening	Pathogen WGS	CEA	Standard of care	2018	US dollars	not specified	The study examined the cost-effectiveness of a pathogen WGS surveillance-based infection prevention program compared to the SOC in a hospital setting. In the base case, 37.9 transmissions were averted and $9,073 was saved by WGS surveillance compared to the SOC.<br>In the base case and in 49% of PSA simulations, WGS surveillance was both less costly and averted more transmissions than the SOC. There was an 80% likelihood of WGS surveillance being cost-effective if the WTP exceeded $2,400 per averted transmission.	[41]
Cost-effectiveness of genome-wide sequencing for unexplained developmental disabilities and multiple congenital anomalies	Li et al.	2021	Original Research Article	Canada	Diagnostic tool	Human WGS & WES	CEA	Standard testing (i.e. first-tier CMA, fragile X, second-tier single-gene tests or gene panels)	2019	Canadian dollars	Cost of WES based on HiSeq X platform	The study evaluated the cost-effectiveness of genome-wide sequencing (WES and WGS) for children with unexplained developmental disabilities and multiple congenital anomalies in Ontario, Canada. It compared six testing strategies placing WES and WGS at different points in the pathway to the standard pathway. Four strategies were dominant (cost-saving, improved outcomes) across all three outcomes presented (number of molecular diagnoses, number of positive findings, and number of active treatment changes). The other two strategies – WES after standard testing and WGS after standard testing – were more costly and had improved outcomes compared to standard testing alone. The least costly strategy was first-tier CMA + second-tier WES (CAD$6,357). The most efficacious strategy across all three outcomes was first-tier WES+CMA. Compared with first-tier CMA + second-tier WES, first-tier WES+CMA had an incremental cost of CAD$11,831 per additional molecular diagnosis, CAD$10,848 per additional positive finding, and CAD$64,082 per active treatment change.	[42]
MSAC application no. 1680	Milverto et al.	2021	HTA	Australia	Diagnostic tool	Human WES	CEA	GJB2/GJB6 gene testing; Standard of care	Not specified	Australian dollars	not specified	This study evaluated the cost-effectiveness of using WES to diagnose genetic causes of hearing impairment in children in Australia. The base case incremental cost per additional proband and/or carrier identified was AU$1,629.	[43]

Paper Name	Paper Authors	Publication Year	Type of article	Country Name	Disease context	Sequencing methods	Type of economic evaluation	Comparator †	Currency year	Currency	Sequencing Platform	Summary and cost-effectiveness results	Reference
MSAC application no. 1675 -- Whole Genome Sequencing for the diagnosis of mitochondrial disease	Morona et al.	2023	HTA	Australia	Diagnostic tool	Human WGS & WES	CEA	Scenario with no genetic testing or conventional genetic testing (gene panels)	2022	Australian dollars	not specified	This report was an HTA of WGS of diagnosis of mitochondrial disease. In adults with suspected mitochondrial disease, the base case ICER was AU$1,832 per additional proband diagnosed with mitochondrial disease, and $1,743 per additional genetic diagnosis for any condition. In children with suspected mitochondrial disease, the base case ICER was $6,721 per additional proband diagnosed with mitochondrial disease, and $2,075 per additional genetic diagnosis for any condition.	[44]
MSAC application no. 1646: Whole genome sequencing of antimicrobial-resistant pathogens	Newton et al.	2021	HTA	Australia	Pathogenic screening	Pathogen WGS	CEA	Phenotypic antimicrobial drug susceptibility testing (pDST), which is usual care	Not specified	Australian dollar	not specified	The study evaluated the cost-effectiveness of whole genome sequencing (WGS) for tuberculosis drug susceptibility testing (DST) in Australia, compared to traditional phenotypic DST. WGS was found to be more effective in identifying drug resistance and could lead to faster and more appropriate treatments for patients. The incremental cost per additional case with pathogen resistance correctly diagnosed is AU$4,551.05.	[45]
Cost-Effectiveness of Whole-Genome vs Whole-Exome Sequencing Among Children With Suspected Genetic Disorders	Nurchis, Radio et al.	2024	Original Research Article	Europe (Italy)	Diagnostic tool	Human WGS & WES	CEA	Standard of care and different strategies in which WES/WGS was performed earlier/later in pathway	2022	euros and US dollars	not specified	This study assessed the cost-effectiveness WGS compared to other strategies involving SOC and WES for diagnosing suspected genetic disorders in a pediatric population in Italy. It found that SOC alone was most likely to be the most cost-effective strategy at WTP thresholds under €23,300 per diagnosis, first-tier WES was most likely to be the most cost-effective strategy at WTP thresholds between €23,300 and €29,800 per diagnosis, and first-tier WGS was most likely to be the most cost-effective strategy at WTP thresholds between €29,800 and €50,000 per diagnosis. We had several concerns with the study's methodology and conclusions drawn about the cost-effectiveness of different strategies. First, as noted in a comment on the article by Scott Grosse (April 26, 2024), the WTP per additional diagnosis is assumed to be the same as the WTP per QALY. Second, the diagnostic yield of SOC followed by WES is lower than SOC alone, which may lack face validity. Third, we recalculated ICERs incrementally to next-best non-dominated options and found that the ICER was €23,208 per additional diagnosis for WES alone compared to SOC, and €29,728 per additional diagnosis for WGS alone compared to WES alone. (SOC-then-WES was dominated, and SOC-then-WGS was extendedly dominated.) This aligns with the comment on the article by Dean Regier (April 26, 2024).	[46]
Bayesian cost-effectiveness analysis of Whole genome sequencing versus Whole exome sequencing in a	Nurchis, Radio et al.	2023	Original Research Article	Europe (Italy)	Diagnostic tool	Human WGS & WES	CEA	SOC testing only; first-line WES; SOC followed by WES; and	2022	euros	not specified	This study ran a simulation model to evaluate the cost-effectiveness of strategies involving WGS, WES and SOC testing in pediatric patients with suspected genetic disorders, comparing five strategies: SOC (no WES/WGS); first-line WES; first-line WGS; second-line WES (following SOC); and second-line WGS	[47]

Paper Name	Paper Authors	Publication Year	Type of article	Country Name	Disease context	Sequencing methods	Type of economic evaluation	Comparator †	Currency year	Currency	Sequencing Platform	Summary and cost-effectiveness results	Reference
pediatric population with suspected genetic disorders								SOC followed by WGS				(following SOC). The study concluded "*that implementing first-line WGS would be a cost-effective strategy, against the majority of the other tested alternatives at a threshold of €30,000–50,000, for diagnosing infants with suspected genetic disorders.*" We came to different cost-effectiveness conclusions after reviewing and attempting to replicate the results based on the evidence presented about each strategy's absolute costs and effects. We estimated that second-line WES was extendedly dominated by SOC alone and second-line WGS, and that first-line WES was extendedly dominated by second-line WGS and first-line WGS. We estimated that the ICER of second-line WGS versus SOC alone was €4,907.43 per additional diagnosis, and that the ICER of WGS alone versus second-line WGS was €63,888.63 per additional diagnosis. Other methodological challenges limited the interpretability of results. Units are not given in the results table. In addition, the threshold used to estimate net monetary benefit is not provided. The study reported the WTP per diagnosis to be €30,000–50,000 because it "*assumed that the threshold that the society is willing to pay for a single QALY is the same amount of money which is willing to pay for one more diagnosis*" which might not be the case.	
Genome-Wide Sequencing for Unexplained Developmental Disabilities or Multiple Congenital Anomalies: A Health Technology Assessment [sections titled 'Primary Economic Evaluation' and 'Budget Impact Analysis']	Ontario Health	2020	HTA	Canada	Diagnostic tool	Human WGS & WES	CEA	Standard genetic testing	2019	Canadian dollars	Several considered in scenario analysis: HiSeq2500 and NextSeq 550 for WES; HiSeq X for WGS	This was an HTA appraisal of WES/WGS for diagnosis of people with unexplained developmental disabilities or multiple congenital anomalies. Six strategies were compared against standard testing which place WES or WGS at different stages of the diagnostic pathway. The two non-dominated strategies were second-tier WES after CMA alone and first-tier WES+CMA. First-tier WES+CMA was more costly and more effective, with the following ICERs compared with second-tier WES after CMA alone: CAD$11,831 per additional molecular diagnosis; $10,848 per additional positive finding; and, $64,082 per additional active treatment change.	[48]
Integrating exome sequencing into a diagnostic pathway for epileptic encephalopathy: Evidence of clinical utility and cost effectiveness	Palmer et al.	2018	Original Research Article	Australia	Diagnostic tool	Human WES	CEA	Standard diagnostic approach	2016	Australian dollars	not specified	The study found that trio-WESfor children with epileptic encephalopathy in Australia is cost-effective (dominant) compared to standard diagnostic pathways. The trio-WES pathway led to a cost saving of AU$5,236 per additional diagnosis (95% CI: $2,482; $9,784) compared to the standard pathway. The standard pathway had a cost per diagnosis ~10 times higher than trio-WES.	[49]
Real-world diagnostic outcomes and cost-effectiveness of genome-wide sequencing for developmental and	Regier et al.	2024	Original Research	Canada	Diagnostic tool	Human WGS & WES	CEA	Standard of care	2021	Canadian dollar	not specified	This study found that both WES and WGS dominated the SOC for the diagnosis of developmental and/or seizure disorders in children. The strategy with the highest NMB was streamlined WES. Net monetary benefit was $2,956 (95%CI = -$608, $6,519) per 1000 patients tested for streamlined WES vs SOC, using a general public WTP threshold of	[50]

Paper Name	Paper Authors	Publication Year	Type of article	Country Name	Disease context	Sequencing methods	Type of economic evaluation	Comparator †	Currency year	Currency	Sequencing Platform	Summary and cost-effectiveness results	Reference
seizure disorders: Evidence from Canada												$9,992/additional diagnosis. 94% of simulations demonstrating cost savings for streamlined WES vs SOC, compared with 60% for first-tier WGS vs SOC.	
The cost-effectiveness of whole genome sequencing in neurodevelopmental disorders	Runheim et al.	2023	Original Research Article	Europe (Sweden)	Diagnostic tool	Human WGS	CEA	CMA	2020	US dollars	NovaSeq 6000	This study compares the cost-effectiveness of Whole Genome Sequencing (WGS) and chromosomal microarray analysis (CMA) as first-line diagnostic tests for neurodevelopmental disorders, finding that WGS yields higher diagnostic rates and lower healthcare costs over a two-year period. The study reported that WGS was dominant and that WGS led to an incremental total cost per patient of WGS: -$2,339 (cost saving). The study also stated that this is not statistically significant because costs and diagnostic yield are not individually statistically significant. We note that this is not the standard method to make statistical inferences about cost-effectiveness, and it is possible for a measure of cost-effectiveness (e.g., an ICER) to be statistically significant even if either costs or effects are not individually statistically significant.	[51]
Diagnostic outcomes and relative cost of clinical whole exome sequencing	Ruzhnikov et al.	2016	Abstract	USA	Diagnostic tool	Human WES	CEA	Prior negative workup	Not specified	US dollars	not specified	The study draws cost-effectiveness conclusions for diagnosing suspected genetic disorders in a population of undiagnosed children using WES, relative to prior testing, with a threshold analysis approach. It did this based on the diagnostic yield of WES in patients who were undiagnosed after prior testing and the mean cumulative cost of prior testing per proband. The study states that WES is cost-effective up to a cost of $8,171 per family trio, which equals the mean prior cumulative testing cost per proband ($16,848) multiplied by the diagnostic yield of WES (48.5%), which indicates that the study's criterion for 'cost-effectiveness' is that the test would be cost-saving if it replaced all prior testing costs in probands diagnosed by WES.	[52]
The budget impact and cost-effectiveness of introducing whole-exome sequencing-based virtual gene panel tests into routine clinical genetics	Sagoo et al.	2017	Report (health economic evaluation)	UK	Diagnostic tool	Human WES	CEA	Usual testing strategy	2015	Great British Pounds	MiSeq and NextSeq	This HTA report evaluated the use of WES for diagnosing a group of patients with a range of clinical scenarios suspected of having a high likelihood of having an inherited genetic disorder. Two scenarios were compared to usual testing: in Scenario 1, WES was added to conventional diagnostic tests already conducted in usual testing; this strategy had an incremental cost per additional positive genetic diagnosis (ICER) of £3,171, compared to usual testing alone. In Scenario 2, WES as near first-line test replaced usual testing; this strategy had an incremental cost per additional genetic diagnosis (ICER) of £2,201, compared to usual testing alone. Since the same diagnostic yield was applied to Scenario 1 and Scenario 2, Scenario 2 was cost-effective relative to Scenario 1. The laboratory price for WES was set at £1,300.	[53]

Paper Name	Paper Authors	Publication Year	Type of article	Country Name	Disease context	Sequencing methods	Type of economic evaluation	Comparator †	Currency year	Currency	Sequencing Platform	Summary and cost-effectiveness results	Reference
Diagnostic yield of genetic tests in epilepsy: A meta-analysis and cost-effectiveness study	Sanchez-Fernandez et al.	2019	CEA	USA	Diagnostic tool	Human WES	CEA	No genetic testing	Not specified	US dollars	not specified	This study estimates the diagnostic yield and cost-effectiveness of genetic tests for epilepsy of unknown etiology and parameterises a cost effectiveness model with the results of a meta-analysis. In the first analysis, three individual tests – CMA, gene panel, and WES – were compared to no genetic testing. In the base case, no genetic testing and WES extendedly dominated CMA and the gene panel. WES was more costly and more effective than no genetic testing, with an ICER of $15,000 per diagnosis. The second analysis compared six tiered strategies of these tests with no genetic testing. In the base case, the following tiered strategy dominated all other tiered strategies: first-tier WES + second-tier gene panel test + third-tier CMA. This strategy was more costly and more effective than no genetic testing, with an ICER of $15,336/diagnosis.	[54]
Cost-effectiveness of massively parallel sequencing for diagnosis of paediatric muscle diseases	Schofield et al.	2017	Perspective	Australia	Diagnostic tool	Human WES	CEA	Traditional diagnostic pathway	2016	Australian dollars	HiSeq 2000 and/or HiSeq 2500	In an analysis of a cohort of patients with childhood-onset muscle disorders (the combined cohort of Congenital MD and Nemaline myopathy patients), the mean cost per diagnosis decreased from AUD$22,596 for the traditional invasive biopsy approach to AUD$5,077 for the gene panel test and AUD$7,734 for WES. The reported incremental saving per additional diagnosis was AUD$23,390 for the panel and $AUD13,732 for WES. However, the reported incremental saving per additional diagnosis for WES was estimated relative to the traditional approach, not the next best non-dominated comparator, which was the gene panel. We have independently calculated the incremental cost per additional diagnosis for WES relative to the panel to be AUD$63,535.††	[55]
Exome sequencing compared with standard genetic tests for critically ill infants with suspected genetic conditions	Smith et al.	2020	Original Research	USA	Diagnostic tool	Human WES	CEA	Cohort with no WES	2017	US dollars	not specified	This study was a CEA of newborns and infants who were admitted to the intensive care unit with suspected genetic etiology within one year of birth. For the WES cohort, the mean cost of the diagnostic pathway per diagnosis was $60,869 (95%CI: $49,749–$71,953); For the no-WES cohort, the mean cost of the diagnostic pathway per diagnosis was $24,763 (95% CI: $19,956–$29,569).	[56]
Meeting the challenges of implementing rapid genomic testing in acute pediatric care	Stark, Lunke et al.	2018	Original research article	Australia	Diagnostic tool	Human WES	CEA	Usual care (single-gene and multi-gene panel testing)	Not specified	Australian dollars	HiSeq 4000 or NextSeq 500	This study implemented rapid rWES in acute pediatric care. The cost per diagnosis and diagnostic yield for the three strategies tested were: AU$27,050 for usual care + conventional sequencing tests (SNP microarray & panel tests), with a diagnostic yield of 17.5%; AU$10,843.60 for usual care + conventional WES (WES with standard turnaround time), with a diagnostic yield of 62.5%; and, AU$13,388 for rWES, with a diagnostic yield of 52.5%. The cost of providing rWES was AU$3,959/patient. Standard WES dominated rWES. The cost per additional diagnosis was AU$$4,541.02 for standard WES	[57]

Paper Name	Paper Authors	Publication Year	Type of article	Country Name	Disease context	Sequencing methods	Type of economic evaluation	Comparator †	Currency year	Currency	Sequencing Platform	Summary and cost-effectiveness results	Reference
												compared to usual care + conventional sequencing tests.***	
Prospective comparison of the cost-effectiveness of clinical whole-exome sequencing with that of usual care overwhelmingly supports early use and reimbursement	Stark, Schofield et al.	2017	Original research article	Australia	Diagnostic tool	Human WES	CEA	Standard diagnostic testing	2015	Australian dollars	not specified	This study is a CEA of singleton WES for diagnosis of infants aged with suspected monogenic disorders. Four strategies were evaluated: standard care (no WES); WES as a last resort for undiagnosed patients once standard care was exhausted; WES as a second-stage test; and, WES as a first-stage text. First-line WES dominated all other options and was dominant compared to standard of care in 97% of simulations in a probabilistic analysis.	[58]
Genomic diagnostics within a medically underserved population: efficacy and implications	Strauss et al.	2018	Original research article	USA	Diagnostic tool	Human WES	CEA	Standard diagnostic approach	Not specified	US dollar	HiSeq 2500	This study assessed the clinical utility of WES for diagnosing underserved populations and provided cost-effectiveness estimates based on a theoretical example. The hypothesized cost per molecular diagnosis was $8,000 in the CMA + WES group, compared to $60,000 using the standard approach.	[59]
Cost-Effectiveness of Earlier Whole-Genome Sequencing in Rare Genetic Childhood Epilepsy	Suokas et al.	2022	Abstract	UK	Diagnostic tool	Human WGS & WES	CEA	CMA+WES	Not specified	British Pounds	not specified	CEA of WGS to diagnose rare genetic childhood epilepsy. In the first year, the ICER of WGS was £26,938 per additional diagnosis compared to CMA+WES; with a five-year time horizon, WGS was dominant, with £5,740 in cost savings.	[60]
Diagnostic Yield and Economic Implications of Whole-Exome Sequencing for ASD Diagnosis in Israel	Tal-Ben Ishay et al.	2021	Original research article	Israel	Diagnostic tool	Human WES	CEA	CMA	Not specified	US dollars	not specified	This study conducts a CEA of three strategies to diagnose Autism Spectrum Disorder in Israel: CMA alone, WES alone, and CMA+WES. The study reports three cost-effectiveness estimates labeled as ICERs, but what was actually estimated was the total cost per diagnosis, divided by 100. The unit was also misreported as USD per child rather than USD per diagnosis. Correcting for this, the total cost per diagnosis was $11,700 for CMA, $14,740 for WES alone, and $12,480 for CMA+WES. The corrected incremental cost per additional diagnosis was $20,370 for WES alone and $9,000 for CMA+WES.**	[61]
Diagnostic Impact and Cost-effectiveness of Whole-Exome Sequencing for Ambulant Children With Suspected Monogenic Conditions	Tan et al.	2017	Original research article	Australia	Diagnostic tool	Human WES	CEA	Standard diagnostic pathway without WES	Not specified	Australian dollars & US dollars	not specified	The study assessed WES for diagnosing children suspected of having a monogenic disorder. It compared strategies in which WES was performed at different stages of the pathway to the standard of care in which no WES is performed. Depending on when WES was performed in the pathway, the cost effectiveness of performing WES ranged from an incremental cost of AU$5,760 per additional diagnosis to an incremental cost savings of $9,020 per additional diagnosis, relative to the standard of care.	[62]
A microcosting and cost-consequence analysis of clinical genomic testing strategies in autism spectrum disorder*	Tsiplova et al.	2017	Original research article	Canada	Diagnostic tool	Human WGS & WES	CEA‡‡	CMA	2015	Canadian dollars	HiSeq 2500 and HiSeq X	This study was a microcosting and CEA of CMA, WES, and WGS for Autism Spectrum Disorder in children. The estimated costs per sample were as follows: CAD$1,655 (£1,227) for WES; $5,519 (£4,092) for WGS on HiSeq2500; and, $2,851 (£2,114) for WGS on HiSeq X. A CEA was then performed on three hypothetical scenarios – WES, WGS on HiSeq2500, and WGS on HiSeq X –	[63]

Paper Name	Paper Authors	Publication Year	Type of article	Country Name	Disease context	Sequencing methods	Type of economic evaluation	Comparator †	Currency year	Currency	Sequencing Platform	Summary and cost-effectiveness results	Reference
												compared to CMA. The ICER of CMA+WES vs CMA was CAD$25,458 per additional patient with a positive finding. WGS with HiSeq X dominated WGS with HiSeq 2500. The ICER of WGS vs CMA+WES was $28,300.	
Health Technology Assessment of next-generation sequencing: Chapter 7: Cost-effectiveness of diagnostic whole exome sequencing in complex paediatric neurology	van Nimwegen et al.	2017	Thesis chapter submitted as an article and corresponding conference abstract	Europe (the Netherlands)	Diagnostic tool	Human WES	CEA	Conventional diagnostic trajectory	2016	Euros	not specified	This study evaluated WES for diagnosing complex paediatric neurologic disorders of suspected genetic origin. The study was published in the form of a thesis chapter and a conference abstract with almost identical titles. The results in the thesis chapter were as follows: "*average costs of the conventional diagnostic trajectory were €1,885; when retrospectively collected costs were included, these amounted to €9,353. The costs of the WES trajectory were €3,809. This resulted in an incremental cost-effectiveness ratio (ICER) of €8,950 per additional diagnosis. Our decision model indicated that WES as a first-tier test could result in cost savings if at least 30% of a paediatric neurology patient population consists of patients with complex paediatric neurological disorders.*" The results in the abstract were similar but not identical; e.g., the ICER was €10,673 per additional diagnosis.	[64, 65]
Diagnostic Yield and Cost-Effectiveness of "Dynamic" Exome Analysis in Epilepsy with Neurodevelopmental Disorders: A Tertiary-Center Experience in Northern Italy	Varesio et al.	2021	Original research article	Europe (Italy)	Diagnostic tool	Human WES	CEA	Standard diagnostic protocol	Not specified	Euros	NovaSeq6000 and NextSeq500	The study found that WES as a first test is cost saving and has a higher diagnostic yield compared to a gene panel for diagnosing epilepsy with neurodevelopmental disorders. The cost per diagnosis is €10,197.88 for the two-step panel test strategy, €13,050.59 for the panel-then-WES strategy, and €5,527.31 for the WES-first strategy. The results indicate that the WES-first strategy is dominant.	[66]
Diagnostic and cost utility of whole exome sequencing in peripheral neuropathy	Walsh et al.	2017	Original Research Article	Australia	Diagnostic tool	Human WES	CEA	none	Not specified	Australian dollars	not specified	This study evaluates the diagnostic utility and cost-effectiveness of WES for individuals with peripheral neuropathy. The cost of performing WES was AU$2,000 per patient. Average cost per diagnosis with WES was AU$16,027 in the study, but would have been AU$12,413 if WES had been implemented earlier in the pathway – a potential cost savings of 22.5%.	[67]
Early-stage economic analysis of research-based comprehensive genomic sequencing for advanced cancer care	Weymann, Laskin et al.	2022	Original Research Article	Canada	Precision oncology	Human WGS	CEA	Usual care	2015	Canadian dollars	not specified	This study estimated the cost-effectiveness of whole genome and transcriptome analysis (WGTA) for cancer patients. In the observed (historical) cohort, incremental survival was -0.03 months and incremental healthcare costs were $5,203 for WGTA versus usual care. This led to a ~0% probability of being cost-effective at a WTP of $100,000 or $150,000 per LYG. In a separate scenario reflecting the current state of the precision oncology program at the time the article was written, net monetary benefit of WGTA was -$12,440 and -$10,244 for WTP thresholds of $100,000 and $150,000 per LYG, respectively. In the most optimistic scenario, WGTA	[68]

Paper Name	Paper Authors	Publication Year	Type of article	Country Name	Disease context	Sequencing methods	Type of economic evaluation	Comparator †	Currency year	Currency	Sequencing Platform	Summary and cost-effectiveness results	Reference
												offered a net monetary benefit of $6,622 and a probability of being cost-effective of 87.6% at a $150,000 WTP threshold.	
Cost-effectiveness of Genome and Exome Sequencing in Children Diagnosed with Autism Spectrum Disorder	Yuen et al.	2018	Original Research Article	Canada	Diagnostic tool	Human WGS & WES	CEA	CMA	2016	Canadian dollars	HiSeq2500 for WES; HiSeq X for WGS	This study employed a microsimulation model to estimate the costs and cost-effectiveness of strategies involving CMA, WES, and/or WGS for autism spectrum disorder diagnosis. The ICER per additional child identified with a rare pathogenic variant within 18 months+ was estimated. CMA, CMA+WES, and WGS were on the cost-effectiveness frontier, while WES alone was extendedly dominated. The ICER of CMA+ES vs CMA alone was CAD$5,808.90 from the public payer perspective and $5,997.80 from the societal perspective; the ICER of WGS vs CMA+WES was $11,886.70 from the public payer perspective and $11,974.91 from the societal perspective.	[69]

Abbreviations: CMA: Chromosomal Microarray; WGS: Whole Genome Sequencing; WGTA: Whole genome and transcriptome analysis; WES: Whole Exome Sequencing; eWES: early WES; SNP: single-nucleotide polymorphism; SOC: Standard of Care; USD: U.S. Dollar; CAD: Canadian Dollar; AU: Australian Dollar; GBP: Great British Pound; UK: United Kingdom; USA: United States of America; CUA: Cost-utility analysis; CEA: Cost-effectiveness analysis; MSAC: Medical Services Advisory Committee; MRSA: methicillin-resistant *Staphylococcus aureus*; carbapenem-resistant *Acinetobacter baumannii* (CRAB); ICER: incremental cost effectiveness ratio; iNMB: incremental net monetary benefit; QALY: quality-adjusted life year; LYG: life-year gained; WTP: Willingness to Pay

Note: Blocks of text that are italicised and enclosed in quotations are direct quotations from the article. Only micro-costing estimates were converted into a common currency year; prices and estimated ICERs or total care costs have not been converted into a common currency year. Micro-cost estimates were converted to 2024 GBP using purchasing price parities (PPPs) and rounded to the nearest currency unit. Exact conversions to 2024 USD and conversions using pure exchange rates are reported in *S.A.6B.*

*This study was also included in the Schwarze et al. (2018) review.

**Calculations for the corrected incremental cost per additional diagnosis figures are as follows: ($2270-$1170)/(0.154-0.1) = $20,370 for WES alone; ($3170-$2270)/(0.254-0.154) = $9,000 for CMA+WES.

***Calculations for the incremental cost per additional diagnosis were not provided in the paper but were estimated as follows: ($271,090.94-$189.352.53)/(25-7)=$4,541.02

****The study did not report the ICER of WES vs no additional testing and was estimated as follows: ($299,939,620-$299,711,290)/(8,492-8,461))= $7,365/QALY

†Comparator: Cost comparisons of the same technology over time were not considered sufficient for the study to have a comparator. Studies which compared two or more diagnostic strategies utilising WES/WGS at different stages of the pathway were considered to have a comparator, even if all strategies involved WES/WGS at some stage.

††The calculation for the incremental cost per additional diagnosis of WES relative to the panel was not performed in the paper but was estimated as follows: ($340,317.35-$213,247.35)/(44-42)=$63,535

†††WES was performed but only 341 genes were analysed. Since the sequencing costs corresponded with WES rather than a gene panel test, it was considered to be WES.

††††These two reports are similar, but came to different cost-effectiveness estimates. It was not clear whether the study populations differed or if the methods were changed. As a result, these reports were included as separate studies.

‡The incremental cost per additional diagnosis was calculated as follows: ($2,683-$2,412)/(0.43-0.25)=$1,506 per additional diagnosis.

‡‡These studies contain a micro-costing analysis of WES and/or WGS.

⁑The incremental cost per additional positive diagnosis was calculated for each comparison as follows: Trio without GCS vs Singleton WES: ($5,263-$5,125)/(0.34-0.281)=$2,339 per additional positive diagnosis; Trio-WES with GCS vs Trio-WES without GCS: (6,138-5,263)/(0.426-0.34)=$10,174 per additional positive diagnosis; Trio vs Singleton WES: ($6,138-$5,125)/(0.426-0.281)=$6,986 per additional positive diagnosis.

⁑⁑The incremental cost per additional diagnosis was calculated as follows: ($93,920-$92,040)/(6-4)=$940.

⁑⁑⁑The incremental cost per additional diagnosis was calculated as follows: ($553,431-$455,597)/(48-46)=$48,917 per additional diagnosis.

⁑⁑⁑⁑The incremental cost per additional diagnosis was calculated as follows: ($12,030-$7,284)/(0.38-0.37)=$474,600 per additional diagnosis in children; ($9,189-$7,540)/(0.57-0.41)=$10,306 per additional diagnosis in critically ill infants. The original ICERs and conclusions were as follows: In the original study, WGS alone and WGS following standard care had ICERs of $15,904 and $19,071 per additional diagnosis, respectively, compared to standard care alone. However, when costs were included from the length of stay in the neonatal intensive care unit, both the WGS alone strategy and WGS following standard care strategy dominated standard care alone.

Summary table 5B: Included primary research studies that are partial economic evaluations

Paper Name	Paper Authors	Year	Type of article	Country	Disease context	Sequencing methods	Type of economic evaluation	Comparator †	Currency year	Currency	Sequencing Platform	Main findings	Reference
Continuing the sequence? Towards an economic evaluation of whole genome sequencing for the diagnosis of rare diseases in Scotland	Abbott et al.	2022	Original research article	UK (Scotland)	Diagnostic tool	Human WGS	Cost study†††	Standard diagnostic testing	2018	GBP	HiSeq	This was a micro-costing study that estimated the cost of trio-WGS in humans to be £6,625 (2024 GBP: £8,274).	[70]
Whole exome sequencing in molecular diagnostics of cancer decreases over time: evidence from a cost analysis in the French setting	Bayle et al.	2021	Research Article	Europe (France)	Precision oncology	Human WES	Cost study†††	none	2015, 2018	Euro	NovaSeq 6000 and HiSeq 2000	The cost per WES sample decreased from €1,921 (£2,052) per sample in 2015 when a HiSeq 2000 sequencer was used to €804 (£842) per sample in 2018 when a NovaSeq 6000 sequencer was used. Since each patient required two samples (one germline, one somatic), the estimated total sequencing costs per patient were €3,842 (£4,104) in 2015 and €1,608 (£1,683) in 2018.	[71]
Short-term costs of integrating whole-genome sequencing into primary care and cardiology settings: a pilot randomized trial	Christensen, Vassy et al.	2018	Research Article	USA	Both healthy population screening and diagnostic tool	Human WES	Cost study	Evaluation based on family history alone.	2015	USD	HiSeq	Incremental total costs for WGS cohort were USD$5,073 for primary care and USD$5,098 for cardiology. Sequencing cost was USD$4,000 based on market rates.	[72]
Cost-Analysis of a Clinical Workflow for Diagnosis of Inherited Kidney Diseases	Cirillo & Becherucci et al.	2022	Abstract	Europe (Italy)	Diagnostic tool	Human WES	Cost study	Strategies with different timings of WES (late vs early)	Not specified	Euros	not specified	Estimated cost per diagnosis decreased from €7,700 for late-WES to €6,340 for early-WES.	[73]
The cost trajectory of the diagnostic care pathway for children with suspected genetic disorders	Dragojlovic et al.	2020	Original Research Article	Canada	Diagnostic tool	Human WGS & WES	Cost study	Comparison to non-WGS patients	2016	Canadian dollars	not specified	Average diagnostic testing costs were CAD$12,006 for patients who received WGS or WES, and CAD$4,972 for patients who did not receive WGS or WES.	[74]
Estimating the costs of genomic sequencing in cancer control	Gordon et al.	2020	Full-length research article	Australia	Precision oncology	Human WGS & WES	Cost study†††	Targeted gene panels	2018	Australian dollar and US dollar	Several platforms: Illumina Xten, NextSeq, MiSeq, BGISEQ-500	Micro-cost estimates were AU$870.63–$2,787.53 (£507-£1,622) per person for WES and AU$2,895–$4,830 (£2,811-£4,050) per person for WGS. When capital costs were included, cost estimates increased to AU$963-$2,984 (£560-£808) for WES and AU$3,007-$4,924 (£1,750-£2,865) for WGS.	[75]
Microcosting of whole genome sequencing (WGS) of trios in heterogeneous pediatric cardiac population	Jegathisawaran, Tsiplova, et al.	2020	Full report	Canada	Diagnostic tool	Human WGS	Cost study†††	none	2019	Canadian dollars	HiSeq X	This micro-cost study estimated that trio-WGS cost CAD$8,035 (£5,586). This was expected to decrease to CAD$7,575.60 (£5,267) in year five due to scale economies.	[76]
Trio genome sequencing for developmental delay	Jegathisawaran,	2022	Original Research Article	Canada	Diagnostic tool	Human WGS	Cost study	none	2019	Canadian dollar	HiSeq X	This micro-cost study estimated that trio-WGS cost CAD$6,634 (£4,612) when used to diagnose	[77]

Paper Name	Paper Authors	Year	Type of article	Country	Disease context	Sequencing methods	Type of economic evaluation	Comparator †	Currency year	Currency	Sequencing Platform	Main findings	Reference
and pediatric heart conditions: A comparative microcost analysis	Tsiplova et al.											developmental delay, and CAD$8,053 (£5,586) when used for cardiac conditions.	
Determining Cost-Optimal Next-Generation Sequencing Panels for Rare Disease and Pharmacogenomics Testing	Katragadda et al.	2021	Original research article	USA	Diagnostic tool	Human WES	Cost study	Panel tests	Not specified	US dollars	NovaSeq	WES alone was estimated to cost $362 if 125,000 samples were sequenced per year, and $388 if 12,500 samples were sequenced per year.	[78]
Reduced resource utilization with early use of next-generation sequencing in rare genetic diseases in an Asian cohort	Nazeha et al.	2022	Original Research	Singapore	Diagnostic tool	Human WES	Cost study	Existing diagnostic pathway	2021	Singapore dollars	not specified	Estimated cost savings relative to existing practice range between SGD$547.06 and SGD$6,002.46 depending on the model and the type of disease. The study assumed that trio-WGS cost SGD$6,000.	[79]
Micro-costing diagnostics in oncology: from single-gene testing to whole- genome sequencing	Pasmans et al.	2021	Original Research Article	Europe (Netherlands)	Precision oncology	Human WGS	Cost study†††	Sanger sequencing	2018	Euros	NextSeq 500	Paired WGS (germline and somatic) for precision oncology in the Netherlands was estimated to cost €2,925 per patient (£3,191) -- significantly higher than other sequencing methods including gene panel testing, sanger sequencing, and FISH.	[80]
The effect of rapid exome sequencing on downstream health care utilization for infants with suspected genetic disorders in an intensive care unit	Rodriguez Llorian et al.	2022	Original research article	Canada	Diagnostic tool	Human WES	Cost study	Standard of care	Not specified	Canadian dollar	Not reported for cohort as a whole; 49% of patients receiving WES participated in program using Ion Proton System [81]	Estimated the cost implications of using trio-rWES as a diagnostic tool for critically ill infants admitted to the intensive care unit. In the weighted model, total costs (including outpatient visits and ICU visits) were higher in the WES group by CAD$12,409.62 (95% CI: -$6,926.84 to $31,746.09) at 26-months of follow-up and CAD$12,048.84 (-$4,742.12 to $28,839.80) at 12-months of follow-up. However, neither difference is statistically significant at the 5% significance level. We note that the study's conclusion – that there is no evidence of increased resource use in the WES group – does not correspond with standard approaches used in health economics; conclusions of economic evaluation are typically made on the basis of expected values rather than inference testing [82], and resource utilisation was >$12,000 higher in patients who received WES in the adjusted model.	[83]
The complete costs of genome sequencing: a microcosting study in cancer and rare diseases from a single center in the United Kingdom	Schwarze et al.	2020	Original Research Article	UK (England)	Precision oncology & diagnostic tool	Human WGS	Cost study†††	none	2016	British Pound Sterling	HiSeq 4000	WGS for diagnosing rare diseases was estimated to cost £7,050 (£9,118) per trio or £2,350 (£3,039) per genome. WGS for precision medicine was estimated to cost £6,841 (£8,848) per patient inclusive of both germline and somatic sequencing, or £3,420 (£4,423) per genome.	[84]
The cost and cost trajectory of whole-genome analysis	Weymann et al.	2017	Full-length research article	Canada	Precision oncology	Human WGS	Cost study	none	2015	Canadian dollars	HiSeq 2500	The study evaluates the costs of applying whole-genome analysis (WGS plus transcriptome sequencing) for precision oncology. The mean total	[85]

Paper Name	Paper Authors	Year	Type of article	Country	Disease context	Sequencing methods	Type of economic evaluation	Comparator †	Currency year	Currency	Sequencing Platform	Main findings	Reference
guiding treatment of patients with advanced cancers												cost per patient was CAD$34,886, of which CAD$19,400 was for WGS and RNA-seq. Costs decreased over the period 2012-2015.	
An additional whole-exome sequencing study in 102 panel-undiagnosed patients: A retrospective study in a Chinese craniosynostosis cohort	Chen, Zhang et al.	2022	Original Research	China	Diagnostic tool	Human WES	Cost-consequence	Gene panels	Not specified	Not specified (dollar sign only)	Only detail provided is that it was an Illumina platform	The study assesses the use of WES for genetic diagnosis of craniosynostosis in children. A strategy of "panel-first", followed by WES for unresolved cases, reduced costs by 24.3% compared to a "WES-only" strategy.	[86]
Rapid whole-exome sequencing facilitates precision medicine in paediatric rare disease patients and reduces healthcare costs	Chung et al.	2020	Original Research Article	Hong Kong SAR, China	Diagnostic tool	Human WES	Cost-consequence	Conventional diagnostic tests	2018/19	Hong Kong Dollars	NextSeq500	The study evaluates rWES to diagnose pediatric patients with suspected monogenic disorders. Rapid WES improves diagnostic yield and is cost-saving, reducing expenditure by $5,325,187 Hong Kong Dollars across 102 patients.	[87]
Use of WGS in Mycobacterium tuberculosis routine diagnosis	Cirillo et al.	2016	Abstract	Europe (Italy)	Pathogenic screening	Pathogen WGS	Cost-consequence	Standard testing (direct susceptibility testing using MGLT)	2016	Euros	Miseq/Miniseq	Using pathogen WGS to diagnose drug-resistant tuberculosis reduced turnaround time relative to standard testing and cost €150/year.	[88]
Whole exome sequencing in neurogenetic odysseys: An effective, cost- and time-saving diagnostic approach	Cordoba et al.	2018	Research Article	Argentina	Diagnostic tool	Human WES	Cost-consequence	Prior diagnostic workup (other tests)	Not specified	US dollars	HiSeq 2500	The study assesses the use of WES for diagnosis of neurogenetic disorders. WES is cost-saving, costing USD$1,000 compared to USD$1,646 for prior diagnostic tests.	[89]
Diagnostic Yield and Treatment Impact of Targeted Exome Sequencing in Early-Onset Epilepsy	Demos et al.	2019	Research article	Canada	Diagnostic tool	Human WES	Cost-consequence	Standard diagnostic testing	2014-2015 fiscal year	Canadian Dollar	Ion Proton	The CCA evaluates the diagnostic yield and costs of WES in patients with early-onset epilepsy. The analysis suggests savings of CAD$5,110 per patient using WES compared to standard diagnostic testing. The study reports outcomes such as diagnostic yield and time to diagnosis.	[90]
Project Baby Bear: Rapid precision care incorporating rWGS in 5 California children's hospitals demonstrates improved clinical outcomes and reduced costs of care	Dimmock et al.	2021	Research Article	USA	Diagnostic tool	Human WGS	Cost-consequence	Counterfactual care pathways	Not specified	US dollars	Only detail provided is that "instruments developed by Illumina"	In the base case with a 3-day rWGS turnaround, cost savings from averted care ($12,041 to $15,786) exceeded the cost of rWGS ($9,492), suggesting that rWGS is cost-saving.	[91]
A hybrid simulation model approach to examine bacterial genome sequencing during a hospital outbreak	Elliott, Lee et al.	2020	Original Research Article	Australia	Pathogenic screening	Pathogen WGS	Cost-consequence	No WGS scenario	2018	Australian dollars	not specified	Pathogen WGS reduced the number of patients who were colonised and total health care costs in a simulated model of a hospital ward with an E. coli outbreak. Sequencing was estimated to cost AUD$354.70.	[92]

Paper Name	Paper Authors	Year	Type of article	Country	Disease context	Sequencing methods	Type of economic evaluation	Comparator †	Currency year	Currency	Sequencing Platform	Main findings	Reference
Evaluating the economic effects of genomic sequencing of pathogens to prioritise hospital patients competing for isolation beds	Elliott, Hare et al.	2020	Original Research Article	Australia	Pathogenic screening	Pathogen WGS	Cost-consequence	Current practice	2018	Australian dollars	not specified	Compared with existing practice, pathogen WGS reduced the number of infections and total health care costs in a simulated model of a hospital ward with a non-multiresistent methicillin-resistent Staphylococcus aureus (nmMRSA) outbreak. Sequencing was estimated to cost AUD$354.70.	[93]
Project Baby Bear: The first state-funded quality improvement project of rapid Whole Genome Sequencing in neonatal and pediatric intensive care units in the USA	Ellsworth	2020	Research Abstract	USA	Diagnostic tool	Human WGS	Cost-consequence	Standard of care	Not specified	US dollars	not specified	For 116 critically ill paediatric patients enrolled in California Medicaid, rWGS generated an estimated total cost savings of up to USD$2.4 million across all episodes of care, and reduced hospital days, procedures and diagnostic tests.	[94]
Rapid whole-genome sequencing decreases infant morbidity and cost of hospitalization	Farnaes et al.	2018	Original Research Article	USA	Diagnostic tool	Human WGS	Cost-consequence	Standard genetic testing	Not specified	US dollar	not specified	The total cost of rWGS (including indirect costs and treatment guidance) ranged from USD$8,482-$22,128 per family (across singleton, duo, trio, and quad rWGS). On average, rWGS cost USD$16,063 per family‡. The study reported outcomes such as diagnostic sensitivity, rate of clinical utility, avoided morbidity, and the length of hospital stay.	[95]
Cost of whole genome sequencing for non-typhoidal Salmonella enterica	Ford et al.	2021	Original Research Article	Australia	Pathogenic screening	Pathogen WGS	Cost-consequence‡‡	Current methods (serotyping and MLVA)	2018	US dollars	not specified	The mean cost of pathogen WGS was USD$83.15 (range: USD$72.92-$95.95) as reported by Australian reference laboratories. For WGS to be cost-equal to current typing methods, 275 (1.9%) of serotyped *Salmonella* cases would need averted; likewise, 1,550 (9.6%) of all notified *Salmonella* cases would need to be averted for WGS to be cost-equal to PCR.	[96]
Cost of exome sequencing in epileptic encephalopathy: is it ‘worth it’?	Forman et al.	2018	Full-length research letter	Europe (Ireland)	Diagnostic tool	Human WES	Cost-consequence	A pathway without WES	Not specified	Euro	not specified	The mean cost of trio-WES (from a commercial laboratory) was €2,500. Estimated cost-savings were €64,837 for 50 patients, or €1,297 per patient.‡‡‡ The diagnostic yield was 42%.	[97]
Budget impact analysis of routinely using whole-genomic sequencing of six multidrug-resistant bacterial pathogens in Queensland, Australia	Gordon et al.	2021	Original Research Article	Australia	Pathogenic screening	Pathogen WGS	Cost-consequence	Current practice	2019	Australian dollar	not specified	Used WGS cost estimate of USD$437 per sample from Elliott, Lee et al. (2020) [92]. WGS of bacterial pathogens was estimated to be cost saving relative to the standard of care, producing an estimated overall cost savings of AUD$30.9 million in 2021.	[98]
The effect of cancer whole exome sequencing and transcriptome analysis (cWES) on the utilization of traditional molecular diagnostic testing	Graf et al.	2018	Research abstract	USA	Precision oncology	Human WES (including transcriptome analysis)	Cost-consequence	Matched historical control patients	Not specified	Not specified	not specified	The overall cost of molecular testing was higher in patients who had received WES compared to matched control patients in all three treatment periods studied: initial diagnosis, relapse, and bone marrow transplant. The WES group were more likely to received targeted therapy, but the difference in median survival between the two groups was not statistically significant.	[99]

Paper Name	Paper Authors	Year	Type of article	Country	Disease context	Sequencing methods	Type of economic evaluation	Comparator †	Currency year	Currency	Sequencing Platform	Main findings	Reference
and overall survival in pediatric blood cancer care													
Care and cost consequences of pediatric whole genome sequencing compared to chromosome microarray	Hayeems et al.	2017	Original Research Article	Canada	Diagnostic tool	Human WGS	Cost-consequence	CMA	Stated as 2016, but elsewhere reported as 2015	Canadian dollars	not specified	The study reported changes in care and cost implications that resulted from paediatric WGS. The average costs per patient for care prompted by WGS which produced a diagnostic result was CAD$179.68 and ranged from CAD$0-$1,212. Costs averted in these patients as a result of the diagnosis averaged CAD$42.95 and ranged from CAD$0-$583. For patients receiving WGS which did not produce a diagnostic result, the average cost per patient of induced care was CAD$76.55 (range: CAD$0-$4,826), and CAD$4.17 of care was averted on average (range: CAD$0-$74).	[100]
Parallel Tests of Whole Exome Sequencing and Copy Number Variant Sequencing Increase the Diagnosis Yields of Rare Pediatric Disorders	Hu et al.	2020	Full-length research article	China	Diagnostic tool	Human WES**	Cost-consequence	none	Not specified	US dollars	NovaSeq 6000	In total, the "direct cost" of parallel sequencing of trio-WES and CNV-seq was approximately USD$600, or USD$200 per person. On a per-patient basis, this was broken down as follows: ~$100 was for library preparation; ~$80 was for WES; ~$10 was for low-pass WGS for CNV-seq; and ~$10 was for Sanger validation. 36.7% of patients were diagnosed using WES.	[101]
Reducing the Cost of the Diagnostic Odyssey in Early Onset Epileptic Encephalopathies	Joshi et al.	2016	Original Research Article	USA	Diagnostic tool	Human WES	Cost-consequence	Traditional diagnostic methods	Not specified	US dollars	HiSeq 2500	The price of WES was USD$6,100 per trio. The total cost of traditional diagnostic tests during the diagnostic odyssey prior to WES ranged from $9,015 to $35,483. All four patients on which WES was performed received a genetic diagnosis as a result.	[102]
Evaluation of a Combined Multilocus Sequence Typing and Whole-Genome Sequencing Two-Step Algorithm for Routine Typing of Clostridioides difficile	Kamboj et al.	2021	Original Research Article	USA	Pathogenic screening	Pathogen WGS	Cost-consequence†††	MLST alone	Not specified	Dollars (country not specified)	MiSeq	Pathogen WGS for *C. difficile* typing cost $164 per isolate, while MLST alone cost $50. A two-tiered approach in which MLST is performed first and WGS is performed only in MLST concordant strains is cost-saving relative to a WGS-first strategy. Turnaround time for WGS is significantly longer than for MLST. The currency country and currency year were not specified in this study. Because costs are reported in dollars and the study took place in the United States, it is assumed that costs are reported in US dollars. For the purposes of the exchange rate calculations and inflation indexing in the main text (USD$164 = £135 2024 PPP GBP), we assume that costs were collected in the year that the article was received by the publication journal (2020).	[103]
Using an experiment among clinical experts to determine the cost and clinical impact of rapid whole exome sequencing in acute pediatric settings	Kapol et al.	2023	Original research article	Thailand	Diagnostic tool	Human WES	Cost-consequence	Control group	2022	Most costs are reported in Thai Baht (THB); certain costs also	NextSeq 500††	Trio-rWES cost ฿80,000 THB (2022USD: ~$2,250, using the study's currency conversion). rWES had a higher diagnostic and prognostic accuracy, but total per-patient health care costs were ฿60,000- ฿70,000 THB higher on average for patients receiving rWES.	[104]

Paper Name	Paper Authors	Year	Type of article	Country	Disease context	Sequencing methods	Type of economic evaluation	Comparator †	Currency year	Currency	Sequencing Platform	Main findings	Reference
										reported in 2022 USD			
A genome sequencing system for universal newborn screening, diagnosis, and precision medicine for severe genetic diseases	Kingsmore et al.	2022	Original research article	USA	Diagnostic tool & newborn screening	Human WGS	Cost-consequence	Newborn screening with mass spectometry of dried blood spots	Not specified	US dollars	not specified	The study estimates the clinical utility of rWGS for universal and diagnostic newborn screening. It reports that costs are approximately $8,500 per newborn for the hospital at which the study was conducted. This was significantly higher newborn screening using mass spectrometry of dried blood spots, for which the highest fee is $220.	[105]
Exome first approach to reduce diagnostic costs and time – retrospective analysis of 111 individuals with rare neurodevelopmental disorders	Klau et al.	2022	Original research article	Europe (Germany)	Diagnostic tool	Human WES	Cost-consequence	Stepwise approach	2021	Euros	not specified	The average cost of WES was €3,461.45 per individual. The average potential cost savings from averting other types of health care resource utilisation was €3,025.56 per individual. WES reduced the length of the diagnostic odyssey.	[106]
Clinical and Economic Outcomes of Genome Sequencing Availability on Containing a Hospital Outbreak of Resistant Escherichia coli in Australia	Lee et al.	2020	Original Research Article	Australia	Pathogenic screening	Pathogen WGS	Cost-consequence	No sequencing	2018	Australian dollars	not specified	Bacterial WGS reduced total hospital costs during a simulated outbreak of drug resistant *E. coli* in a hospital. Total hospital costs were AU$766,921 in the scenario with no sequencing, AU$473,116 in the actual outbreak with ‘late’ sequencing; and AU$65,374 with early routine sequencing. WGS was also associated with fewer colonised patients. WGS sequencing costs per patient were estimated to be AU$354.70.	[107]
Costs And Effectiveness Of Whole Exome Sequencing (WES) In Patients With Unsolved Rare Disease Through The Diagnostic Pathway	Marshall et al.	2023	Abstract	Canada	Diagnostic tool	Human WES	Cost-consequence	Molecular and specialised diagnostic tests without WES	2020	Canadian Dollar and US dollar	not specified	The diagnostic yield for WES was 40%, c.f. 5% for molecular and diagnostic tests alone. The WES-first scenario had lower test costs (CAD$2,800) than the no WES scenario (CAD$2,700) and WES as a second-line (CAD$2,609), third-line (CAD$4,500), and fourth-line (CAD$5,300) test. The study concluded that placing WES early in the pathway dominates no WES and WES later in the pathway.	[108]
Cost Effectiveness of Whole Exome Sequencing for Children with Developmental Delay in a Developing Country: A Study from Jordan	Masri & Hamamy	2022	Original research article	Jordan	Diagnostic tool	Human WES	Cost-consequence	Traditional diagnostic testing	Not specified	Jordanian Dinar (JOD) and US dollar	not specified	The study analysed a sample of 26 Jordanian families and reports that 14 received WES and six received a final diagnosis, although it was not clear whether all six diagnosed families received WES. The price of WES offered by several laboratories was reported to be USD$1,200. WES results could be used for future family planning and potentially limit the cost of traditional investigations (estimated JOD600/USD847 per child) and indirect costs of rehabilitation and treatment (estimated JOD4,676/USD6,585 per child) for the family’s future children.	[109]
Real-Time Genome Sequencing of Resistant Bacteria Provides Precision Infection Control in	Mellmann et al.	2016	Original research article	Europe (Germany)	Pathogenic screening	Pathogen WGS	Cost-consequence†††	Pulsed-field gen electrophoresis (PFGE); Scenario	Not specified	Euro	MiSeq	The study analyses WGS of multidrug-resistant (MDR) bacteria in a hospital ward. Mean turnaround time for all isolates was 5.3 days. A micro-cost analysis estimated the cost of WGS to be €202 per isolate, compared to approximately	[110]

Paper Name	Paper Authors	Year	Type of article	Country	Disease context	Sequencing methods	Type of economic evaluation	Comparator †	Currency year	Currency	Sequencing Platform	Main findings	Reference
an Institutional Setting								without pathogen WGS				€100 per isolate for pulsed-field gel electrophoresis (PFGE). No currency year was specified in this study. Data collection took place in two phases: the first from October 2013 to April 2014 and the second from October 2014 to April 2015. The currency year was assumed to be 2014 for the purposes of exchange rate calculations and inflation indexing in the main text (€202.49 = £239 2024 PPP GBP). In the second six-month period of time considered in the study, total expenditure on WGS amounted to €111,372 while averted costs due to WGS (primarily the result of fewer beds being blocked as part of isolation measures for patients with MDR infections) were €317,180. Thus, the net cost savings were greater than €200,000.	
Impact of whole exome sequencing (WES) on costs and medical decision-making	Middelburg et al.	2016	Abstract	Europe (Netherlands)	Diagnostic tool	Human WES	Cost-consequence	No WES-first scenario (not specified in detail)	not specified	Euros	not specified	Using WES as a first-stage tool for diagnosis of children suspected for intellectual disability was expected to save €5,122 per patient by replacing other genetic diagnostic approaches and averting 50% of non-genetic diagnostic approaches and consults. 35% of patients who underwent WES received a genetic diagnosis.	[111]
The clinical utility of rapid exome sequencing in a consanguineous population	Monies et al.	2023	Original Research	Saudi Arabia	Prenatal screening & diagnostic tool	Human WES	Cost-consequence	Standard of care (few details given)	Inconsistently reported (some cost components specified as 2022)	USD	Novaseq 6000	The study assesses the clinical utility of rapid whole exome sequencing (rWES). 42% of the cohort receiving rWES had a positive molecular finding. rWES reportedly cost USD$2,000 but no details were given of methods or source of this estimate. In the cohort of 189 cases, rWES cost USD$378,000 but generated healthcare cost savings worth over USD$4,333,390. As a result, net savings were at least USD$3,955,390, representing a return on investment of over 10x.	[112]
Assessment of a Targeted Gene Panel for Identification of Genes Associated With Movement Disorders	Montaut et al.	2018	Original research article	Multiple (France, Luxembourg, Algeria)	Diagnostic tool	Human WES	Cost-consequence	Gene panel	Not specified	Not specified (confirmed to be USD by corresponding author)	NextSeq550, HiSeq2500*	The cost of WES (sequencing alone) was reported as $850-$1113/patient, versus $156/patient for gene panel. No details were provided of methods or source of the WES cost estimate. Time to results were higher for WES than the gene panel, and the diagnostic yield of the two approaches were similar.	[113]
Implementation of next generation sequencing into pediatric hematology-oncology practice: moving beyond actionable alterations	Oberg et al.	2016	Original Research Article	USA	Precision oncology	Human WES	Cost-consequence	none	Not specified	US dollars	not specified	The study assesses the clinical utility of WES and RNA-seq. The cost of performing WES (germline and somatic) was estimated to be $4,459, although reimbursement by payers was less than this figure. No details were provided of methods or source of the WES cost estimate.	[114]
Medical costs of children admitted to the neonatal intensive care unit: The role and possible economic	Olde Keizer et al.	2022	Original Research Article	Europe (Netherlands)	Diagnostic tool	Human WES	Cost-consequence	Current diagnostic pathway (no WES)	2020	euros	not specified	The study reports the number of diagnoses made and cost implications for scenarios representing different strategies of WES testing for neonates admitted to the neonatal intensive care unit. The reported cost per-sample of WES (€1,803) was sourced from available prices. Total costs of WES	[115]

Paper Name	Paper Authors	Year	Type of article	Country	Disease context	Sequencing methods	Type of economic evaluation	Comparator †	Currency year	Currency	Sequencing Platform	Main findings	Reference
impact of WES in early diagnosis												including genetic counseling was €2,892 per sample, or €6,498 per trio. Depending on the scenario, implementing trio-WES increases overall health care costs by up to 22.2%., but would lead to more conclusive genetic diagnoses.	
Rapid exome sequencing as a first-tier test in neonates with suspected genetic disorder: results of a prospective multicenter clinical utility study in the Netherlands	Olde Keizer et al.	2023	Original Research	Europe (Netherlands)	Diagnostic tool	Human WES	Cost-consequence	Conventional genetic diagnostic workup	2020	Euros	not specified	The study reports the unit price of singleton WES (€1,803) and of trio-WES (€5,409). Average costs of genetic diagnostic tests were €85 (1.5%) higher for routine genetic testing (€5,494) relative to rWES alone (€5,409), although this difference was not statistically significant (p=0.23). The rate of conclusive genetic diagnosis was higher with rWES (20%) than routine genetic testing (10%), with a shorter time-to-diagnosis (15 days vs. 59 days, p<0.001).	[116]
Efficacy and economics of targeted panel versus whole-exome sequencing in 878 patients with suspected primary immunodeficiency	Platt et al.	2020	Brief report	Multiple (28 countries)	Diagnostic tool	Human WES	Cost-consequence	Other pathways involving WES (panel test then WES)	Not specified	not specified (dollar sign only)	Only detail provided is that it was an Illumina platform (in supplement).	The price of WES was reported to be $2,500 per patient, and the price of the NGS panel was reported to be $1,700 per patient. The tiered approach (NGS then WES) estimated to have a testing cost of between $2,800 and $3,450 per patient, depending on the diagnostic yield. Thus, savings of a WES-only approach was estimated to range from $300 to $950 per patient.	[117]
Cost-effectiveness of exome sequencing: an Italian pilot study on undiagnosed patients	Radio et al.	2019	Original Research Article	Europe (Italy)	Diagnostic tool	Human WES	Cost-consequence	none	Not specified	Euro	HiSeq 3000 and NextSeq 550⁑	The price of trio-based clinical exome or whole exome sequencing. €3,000. This was lower than the estimated cost to reach a diagnosis for undiagnosed patients (€11,572).	[118]
Effectiveness of clinical exome sequencing in adult patients with difficult-to-diagnose neurological disorders	Sainio, Aaltio et al.	2021	Original Research	Europe (Finland)	Diagnostic tool	Human WES***	Cost-consequence	Standard methods	2018	Euros	Illumina HiSeq 2500***	Total diagnostic costs (including genetic testing, imaging, laboratory assays, examinations, procedures, and inpatient and outpatient clinical care) prior to WES were €5,200 per patient (range: €90 to €13,400). The diagnostic yield was 27% in the entire cohort and 28% in the cohort used for the cost analysis. WES would be cost-saving if priced below €2,500. (We note that the study reported this as the price at which WES becomes "cost-effective", but reflects the threshold at which it becomes cost saving without considering the additional value of diagnosis.)	[119]
Rapid whole genome sequencing impacts care and resource utilization in infants with congenital heart disease	Sweeney et al.	2021	Original Research Article	USA	Diagnostic tool	Human WGS	Cost-consequence	CMA, gene panel test	Not specified	Not specified	HiSeq X, HiSeq 2500, HiSeq 4000	The authors performed a unit cost analysis of the direct and indirect sequencing costs for rWGS; estimated costs were $8,482, $13,031, $17,579, and $22,128 for singleton, duo, trio, and quad sequencing, respectively. Total sequencing costs were $371,864 across 24 families, which is $15,494 per family.⁑⁑ Genetic diagnosis was made in 43% of patients using rWGS versus 10% using microarray and/or gene panels (p=0.02), and average daily total hospital spending decreased after rWGS results.	[120]

Paper Name	Paper Authors	Year	Type of article	Country	Disease context	Sequencing methods	Type of economic evaluation	Comparator †	Currency year	Currency	Sequencing Platform	Main findings	Reference
Dynamic Simulation Modeling to Analyze the Impact of Whole Genome Sequencing National Implementation Scenarios in Lung cancer on Time-to-Treatment, Costs and Patient Demand	van de Ven et al.	2023	Pre-print	Europe (Netherlands)	Precision oncology	Human WGS	Cost-consequence	Standard of care	2023	Euros	not specified	This study reported the findings of a simulation model of nationwide implementation of WGS for precision oncology in the Netherlands. Relative to the standard of care, using WGS as an upfront test in all lung cancer patients reduced time-to-treatment by 33%, increased the cost per patient by 210%, and increased total annual diagnostic costs by nearly 600% (€24.5 million vs €4.2 million). A cost estimate for WGS of €2,925 per patient was used in the base case, sourced from Pasmans et al., 2021 [80].	[121]
A clinical utility study of exome sequencing versus conventional genetic testing in pediatric neurology	Vissers et al.	2017	Original research article	Europe (Netherlands)	Diagnostic tool	Human WES	Cost-consequence	Standard of care pathway	2016	Euros	not specified	WES provided a higher diagnostic yield compared to the standard diagnostic pathway, while also being cost-saving by reducing the number of additional tests needed. The study used the following prices for WES: €1,800 for singleton-WES and €3,500 for trio-WES. The average cost per patient was €3,420. The results indicate that replacing conventional genetic testing with WES would result in an average cost saving of €744 per patient.	[122]
Whole-exome sequencing in intellectual disability; cost before and after a diagnosis	Vrijenhoek et al.	2018	Original research article	Europe (Netherlands)	Diagnostic tool	Human WES	Cost-consequence	none	Not specified	Euros	not specified	The study found that per patient health care costs declined by ~80% after trio-WES regardless of the diagnostic outcome. This held both when WES was performed at the end of the diagnostic trajectory and when WES was performed as a first-tier diagnostic test. The diagnostic yield of trio-WES was 35%.	[123]
Whole genome and transcriptome sequencing in neuromuscular disorders: a diagnostic and health economic analysis	Yang, Chen et al.	2023	Preprint	China	Diagnostic tool	Human WGS & WES	Cost-consequence	Several alternative pathways involving WES/WGS	Not specified	USD	DNBSEQ-T7	The study presents costs and diagnostic yield for eight diagnostic strategies with different combinations of WGS, WES, and RNA-seq. Total pathway costs ranged from $36,930 for WES→RNA-seq to $92,994 for WGS+WES+RNA-seq. The study used the lowest sequencing prices and shortest turnaround times available across different laboratories. Costs and diagnostic yield are compared individually to the standard of care. The study reports that WGS→RNA-seq is a cost-effective strategy, but does not compare this strategy against the next-best non-dominated comparator and does not specify a cost-effectiveness threshold.	[124]
Incorporation of exome-based CNV analysis makes trio-WES a more powerful tool for clinical diagnosis in neurodevelopmental disorders: A retrospective study	Zhai, Zhang et al.	2021	Original Research Article	China	Diagnostic tool	Human WES	Cost-consequence	Different trio-WES and CNV analysis strategies	Not specified	Not specified	NovaSeq 6000	The strategy analysed in the study, trio-WES and WES-based CNV analysis, cost $1,300 with turnaround time of approximately 3 weeks. The overall diagnostic rate was 54%, of which 35% was due to trio-WES. The cost of other strategies involving WES ranged from $1,660 to $2,380 per patient, and turnaround times ranged from three to seven weeks.	[125]

Abbreviations: CNV: copy number variant; CMA: Chromosomal Microarray; MLST: Multi-Locus Sequence Typing; MLVA: Multiple-Locus Variable-Number Tandem Repeat Analysis; PFGE: Pulsed-field gel electrophoresis; FISH: Fluorescent in situ hybridisation; WGS: Whole Genome Sequencing; rWGS: rapid Whole Genome Sequencing; WGTA: Whole Genome and Transcriptome Analysis; WES: Whole Exome Sequencing; SOC: Standard of Care; USD: U.S. Dollar; CAD: Canadian Dollar; AU: Australian Dollar; GBP: Great British Pound; SGD: Singapore Dollars; SAR: Special Administrative Region; THB: Thai baht.

Paper Name	Paper Authors	Year	Type of article	Country	Disease context	Sequencing methods	Type of economic evaluation	Comparator †	Currency year	Currency	Sequencing Platform	Main findings	Reference

†Comparator: Cost comparisons of the same technology over time were not considered sufficient for the study to have a comparator. Studies which compared two or more diagnostic strategies utilising WES/WGS at different stages of the pathway were considered to have a comparator, even if all strategies involved WES/WGS at some stage.
††This information was not included in the original text. However, the original text references another publication with more information about the patient cohort (reference 9: Kamovisit et al., 2021) which noted that the NextSeq500 sequencing platform was used [126].
†††This study contained a microcosting analysis of WES and/or WGS.
‡The article states that rWGS was performed in 42 families at a total cost of USD$674,645. The average cost of rWGS per family (USD$16,063) was not reported in the article, but was calculated independently by dividing the total cost (USD$674,645) by the number of families (42) and rounding to the nearest dollar.
‡‡The article reports the number of cases that would need to be averted for WGS to be cost-equal to other testing methods. This was categorised as a cost-consequence analysis, rather than a cost-effectiveness analysis, because costs and outcomes are not brought together as a ratio; rather, they are presented as a sensitivity analysis.
‡‡‡The article reports that cost savings totaled €64,837 across 50 patients. The average cost of rWES per €1,297 per patient was not reported in the letter, but was calculated independently by dividing the total cost savings (€64,837) by the number of patients (50) and rounding to the nearest euro.
*There is conflicting information about the sequencing platform used in the methods of the main article and the supplemental methods. The main article reports a HiSeq 4000 was used for the gene panel and WES. The supplemental methods reports that a HiSeq 2500 was used for WES and a NextSeq 550 was used for the gene panel. The corresponding author stated in a private communication that the HiSeq 4000 was not used.
**This study conducted WES (100x coverage), as well as low-coverage WGS (0.3x coverage) for copy number variant (CNV) detection that cost USD$10 per sample. This study was not considered to assess the cost of human WGS because the sequencing used was "low-pass WGS (0.3x)-based CNV-seq" and therefore is not representative of typical human WGS, which has higher coverage and is orders of magnitude more costly. The cost estimate of USD$10 for low-coverage WGS was not considered when estimating the range of WGS cost estimates in included reports or in the time-trend analysis.
***Sainio, Aaltio et al. (2021) states that clinical exome sequencing was performed using methods detailed in a different study, Sainio, Välipakka et al. (2019) [127]. The referenced study stipulates that WES was performed; therefore, Sainio, Aaltio et al. (2021) was considered to have analysed WES. Information about the sequencing platforms used was also taken from Sainio, Välipakka et al. (2019).
⁑This study assessed both clinical exome sequencing and WES. Clinical exome analysis was performed on an Illumina MiSeq or NextSeq 550 platform.
⁑⁑The supplementary appendix of this article reports that the total cost of sequencing 24 enrolled families was $371,864. The cost per family was not reported but was calculated independently by dividing total cost ($371,864) by the number of families (24).

Supplementary Appendix 6:

Supplementary Table 6: Study characteristics of primary research publications excluding abstract-only studies

Category	Sub-Category	Number of Studies (%)
**Year of Publication**	2016*	4 (3.8%)
	2017	11 (10.5%)
	2018	13 (12.4%)
	2019	7 (6.7%)
	2020	17 (16.2%)
	2021	22 (21.0%)
	2022	16 (15.2%)
	2023	11 (10.5%)
	2024*	4 (3.8%)
**Country**	United States of America	21 (20.0%)
	United Kingdom	6 (5.7%)
	Australia	25 (23.8%)
	Europe (excl. UK)	23 (21.9%)
	Canada	15 (14.3%)
	China (incl. Hong Kong SAR)	7 (6.7%)
	Country not listed above	6 (5.71%)
	Multiple countries	2 (1.90%)
	Not reported	0 (0.0%)
**Type of Economic Evaluation**	Cost-Utility Analysis (CUA)	14 (13.3%)
	Cost-Effectiveness Analysis (CEA)	42 (40.0%)
	Cost-Consequence Analysis (CCA)	36 (34.3%)
	Cost Study	13 (12.4%)
**Sequencing Type**	Both human WGS and WES	16 (15.2%)
	Human WGS only	22 (21.0%)
	Human WES only	54 (51.4%)
	Pathogen WGS only	13 (12.4%)
**Application†**	Diagnostic tool only	76 (72.4%)
	Pathogenic screening only	13 (12.4%)
	Precision oncology only	10 (9.5%)
	Prenatal screening only	2 (1.9%)
	Newborn screening only	0 (0.0%)
	Healthy population screening only	0 (0.0%)
	Prenatal screening & diagnostic tool	1 (1.0%)

	Health population screening & diagnostic tool	1 (1.0%)
	Precision oncology & diagnostic tool	1 (1.0%)
	Newborn screening & diagnostic tool	1 (1.0%)
**Discount Rate†**	>0%, <1.5%	0 (0.0%)
	≥1.5%, <3%	4 (3.8%)
	≥3%	15 (14.3%)
	Different discount rate categories applied to costs and effects	5 (4.8%)
	Not applied / Not specified	81 (77.1%)
**Study Perspective†**	Societal	5 (4.8%)
	Health system, hospital, or payer***	69 (65.7%)
	Cost of testing only	9 (8.6%)
	Multiple/Other	22 (21.0%)
**Time Horizon†**	<1 year	4 (3.8%)
	≥1 year, <5 years	18 (17.1%)
	≥5 years, <10 years	4 (3.8%)
	≥10 years, <25 years	5 (4.8%)
	≥25 years, but not lifetime	1 (1.0%)
	Lifetime	3 (2.9%)
	Multiple/other	9 (8.6%)
	Unclear; not reported, applicable, or specified	61 (58.1%)
**Currency†**	Report currency	97 (92.4%)
	Report currency year	63 (60.0%)
	Currency year reported inconsistently or only for certain cost elements	2 (1.9%)

* The search period was 28 July 2016 to 7 May 2024.
***Studies that report both health system and personal budget perspectives are included under this category.
†: Items in the CHEERS checklist.
Abbreviations: SAR: Special Autonomous Region; WGS: whole genome sequencing; WES: whole exome sequencing; UK: United Kingdom

Supplementary Appendix 7:

Summary table 7A: breakdown of micro-costing estimates by sequencing type and clinical context.

	**Number of studies**	**Number of estimates**	**References**
**Type of sequencing**			
Singleton-WGS of humans	4	9	[38, 63, 75, 84]
Paired-WGS of humans	2	2	[80, 84]
Trio-WGS of humans	4	5	[38, 70, 76, 84]
WGS of pathogens	2	2	[103, 110]
Singleton-WES	4	6	[38, 63, 71, 75]
Paired-WES	2	5	[71, 75]
Trio-WES	0	0	
**Clinical context**			
Prenatal screening	0	0	
Newborn screening	0	0	
Healthy population screening	0	0	
Diagnostic tool	5	12	[38, 63, 70, 76, 84]
Pathogenic screening	2	2	[103, 110]
Precision oncology	4	15	[71, 75, 80, 84]

Summary table 7B: study-level currency conversions of micro-costing estimates

Authorship & publication year	Micro-cost estimate	Currency	Index year (forecast year if applicable)	Purchasing Power Parity (PPP)		Pure exchange rate	
				2024 GBP	2024 USD	2024 GBP	2024 USD
Abbott et al., 2022	6,625	GBP	2018	£8,274.36	£11,922.71	£8,695.17	$10,287.18
Bayle et al., 2021	804	euro	2018	£841.56	£1,212.62	£887.75	$1,104.56
Bayle et al., 2021	1,921	euro	2015	£1,683.12	£2,425.24	£1,869.11	$2,690.45
Bayle et al., 2021	1,608	euro	2018	£2,051.82	£2,956.51	£1,775.50	$2,209.13
Bayle et al., 2021	3,842	euro	2015	£4,103.63	£5,913.02	£3,738.23	$5,380.91
Gordon et al., 2020	871	AUD	2018	£506.65	£730.04	£608.35	$757.02
Gordon et al., 2020	963	AUD	2018	£560.40	£807.50	£672.90	$837.33
Gordon et al., 2020	2,788	AUD	2018	£1,622.16	£2,337.40	£1,947.79	$2,423.77
Gordon et al., 2020	2,984	AUD	2018	£1,684.70	£2,427.52	£2,085.12	$2,594.66
Gordon et al., 2020	2,895	AUD	2018	£1,736.53	£2,502.20	£2,022.89	$2,517.22
Gordon et al., 2020	3,007	AUD	2018	£1,749.67	£2,521.14	£2,100.90	$2,614.30
Gordon et al., 2020	4,830	AUD	2018	£2,810.74	£4,050.05	£3,374.97	$4,199.71
Gordon et al., 2020	4,924	AUD	2018	£2,865.17	£4,128.48	£3,440.32	$4,281.03
Jegathisawaran, Tsiplova et al., 2019/2020	1,960	CAD	2018	£1,384.02	£1,994.26	£1,414.55	$1,759.23
Jegathisawaran, Tsiplova et al., 2019/2020	1,981	CAD	2018	£1,398.85	£2,015.63	£1,429.70	$1,778.08
Jegathisawaran, Tsiplova et al., 2019/2020	3,143	CAD	2018	£2,219.65	£3,198.35	£2,268.62	$2,821.41
Jegathisawaran, Tsiplova et al., 2019/2020	4,072	CAD	2018	£2,875.58	£4,143.48	£2,939.01	$3,655.16
Jegathisawaran, Tsiplova et al., 2019/2020	3,350	CAD	2018	£2,365.54	£3,408.56	£2,417.72	$3,006.84
Jegathisawaran, Tsiplova et al., 2019/2020	6,556	CAD	2018	£4,629.40	£6,670.60	£4,731.52	$5,884.44
Jegathisawaran, Tsiplova et al., 2019/2020	1,698	CAD	2018*	£1,198.80	£1,727.38	£1,225.24	$1,523.80
Jegathisawaran, Tsiplova et al., 2019/2020	1,747	CAD	2018*	£1,233.40	£1,777.23	£1,260.61	$1,567.78
Jegathisawaran, Tsiplova et al., 2019/2020	2,911	CAD	2018*	£2,055.20	£2,961.38	£2,100.53	$2,612.36
Jegathisawaran, Tsiplova et al., 2019/2020	5,803	CAD	2018*	£4,097.54	£5,904.23	£4,187.93	$5,208.39
Jegathisawaran, Tsiplova et al., 2020	7,576	CAD	2019	£5,266.87	£7,589.15	£5,444.82	$6,458.54
Jegathisawaran, Tsiplova et al., 2020	8,035	CAD	2019	£5,586.33	£8,049.47	£5,775.08	$6,850.29
Jegathisawaran, Tsiplova et al., 2022	6,634	CAD	2018	£4,612.31	£6,645.98	£4,670.40	$5,790.80
Kamboj et al., 2021	164	USD**	2020**	£134.93	£194.42	£152.13	$178.22
Mellmann et al., 2016	202	euro	2014***	£238.69	£343.93	£223.07	$348.61
Pasmans et al., 2021	2,925	euro	2018	£3,190.65	£4,597.48	£3,229.69	$4,018.47

Authorship & publication year	Micro-cost estimate	Currency	Index year (forecast year if applicable)	Purchasing Power Parity (PPP)		Pure exchange rate	
				2024 GBP	2024 USD	2024 GBP	2024 USD
Schwarze et al., 2020	6,841	GBP	2016	£8,848.00	£12,749.28	£8,978.39	$11,277.56
Schwarze et al., 2020	2,350	GBP	2016	£3,039.44	£4,379.59	£3,084.23	$3,874.03
Schwarze et al., 2020	3,420	GBP	2016	£4,423.35	£6,373.71	£4,488.54	$5,637.96
Schwarze et al., 2020	7,050	GBP	2016	£9,118.31	£13,138.78	£9,252.69	$11,622.10
Tsiplova et al., 2017	1,655	CAD	2015	£1,227.19	£1,768.28	£1,136.29	$1,635.45
Tsiplova et al., 2017	2,851	CAD	2015	£2,114.03	£3,046.15	£1,957.45	$2,817.32
Tsiplova et al., 2017	5,519	CAD	2015	£4,092.36	£5,896.77	£3,789.25	$5,453.80

Acronyms: AUD: Australian Dollar; CAD: Canadian Dollar; GBP: Great British Pound; USD: U.S. Dollar.
*Forecasts for future years.
**The currency country and currency year were not specified in this study. Because costs are reported in dollars and the study took place in the United States, it is assumed that costs are reported in US dollars. For the purposes of the exchange rate calculations and inflation indexing, we assume that costs were collected in the year that the article was received by the publication journal (2020).
***The currency year was not reported but was assumed to be 2014 for the purposes of exchange rate calculations and inflation indexing because data were collected during 2013-2015.

Supplementary Appendix 8:

Summary table 8: Included reviews and meta-analyses

Paper Name	Paper Authors	Publication Year	Type of review	Structured search strategy	Study Types	Disease context	Age restriction on patient population	Range of publication years in search strategy	Number of studies included	Sequencing methods	Summary of findings	Reference
Economic evaluation of genomic sequencing in the paediatric population: a critical review	Alam et al.	2018	Systematic and critical review	yes	Costs & effects	Multiple	Newborns and children only	2010-2017	11	WES + WGS (humans only)	Review of economic evaluations for genomic sequencing in paediatrics. Of the seven studies which report a measure of cost-effectiveness, six find genomic sequencing to be cost-effective. Most studies did not meet most of the criteria for a well-designed economic evaluation.	[128]
Economic evidence on identifying clinically actionable findings with whole-genome sequencing: a scoping review	Douglas et al.	2016	Scoping review	yes	Costs & effects	Diagnostic tool	No age restriction	1994-Nov. 2014	32	WGS (humans only)	Scoping review of economic evaluations of WGS for identifying clinically actionable findings for a set of genetic mutations. Based on studies identified by the search, economic evidence was only available about seven of 24 conditions for which the American College of Medical Genetics (ACMG) recommends sharing secondary findings with patients after WGS.	[129]
Report - Cost and Clinical Utility of WES and WGS in pediatric patients with suspected genetic disease	Douglas et al.	2022	Systematic review pre-print	yes	Costs & effects	Diagnostic tool	Paediatric patients only (aged 0-18 years)	Not specified	5 cost studies; 62 comparative effectiveness studies	WES + WGS (humans only)	This study is a review of evidence reporting cost and cost utility estimates for genome sequencing and exome sequencing relative to the standard of care in paediatric patients with diseases with a suspected genetic cause. It found limited evidence that genome sequencing has a larger impact than exome sequencing in causing a change in management. It also concluded, based on available evidence, that rapid genome sequencing provides greater benefits over exome sequencing in the intensive care unit setting than in the outpatient setting.	[130]
The cost and diagnostic yield of exome sequencing for children with	Dragojlovic et al.	2017	Literature review**	yes	Costs & effects	Diagnostic tool	Paediatric	2013-2017	28 total studies: 18 reporting outcomes, 10 reporting costs	WES	Literature review of the costs, diagnostic yield, and cost per positive diagnosis of WES in the context of children with suspected genetic disorders. The mean diagnostic yield across included studies was 26.6% for singleton	[30]

Paper Name	Paper Authors	Publication Year	Type of review	Structured search strategy	Study Types	Disease context	Age restriction on patient population	Range of publication years in search strategy	Number of studies included	Sequencing methods	Summary of findings	Reference
suspected genetic disorders: a benchmarking study											WES and 34.4% for trio-WES. The mean cost of delivering WES service across included studies was CAD$2,726 for singleton-WES and CAD$6,720 for trio-WES, in 2016 Canadian Dollars.	
Next Generation Sequencing Methods for Diagnosis of Epilepsy Syndromes	Dunn et al.	2018	Narrative review	no	Costs & effects	Diagnostic tool	No age restriction	Not applicable	n/a	WES + WGS (humans only)	Narrative review of NGS (including gene panels, WGS and WES) for epilepsy diagnosis, including a section on cost-effectiveness.	[131]
Systematic Review of the Economic Evaluation of Returning Incidental Findings in Genomic Research	Fontes Marx et al.	2021	Systematic review	yes	Costs & effects	Diagnostic tool -- returning incidental findings	No age restriction	up until 08 April 2020	6	WES + WGS (humans only)	This systematic review considered economic evaluations of returning incidental findings of genomic sequencing. Although patients valued the return of incidental findings, evidence about cost-effectiveness was lacking. The cost and cost-effectiveness of returning incidental findings varied between populations with different primary health conditions.	[132]
Value-based genomics	Gong et al.	2018	Review	no	Costs & effects	Precision oncology	No age restriction	not specified	n/a	WES + WGS (humans only)	This review summarises the findings of fifteen studies assessing the costs and cost-effectiveness of NGS for cancer. Seven of the studies were of WES/WGS.	[133]
The Role of Genome Sequencing in Neonatal Intensive Care Units	Kingsmore & Cole	2022	Review	no	Costs & effects	Diagnostic tool	Newborns only	Not specified	n/a	WES + WGS (humans only)	This is a review of genome sequencing as a diagnostic tool in neonatal ICUs. There is not a defined search strategy or study methods. A section of the review summarises economic studies.	[134]
Evaluating genetic and genomic tests for heritable conditions in Australia: lessons learnt from health technology assessments	Norris et al.	2022	"review and narrative synthesis"	yes	Costs & effects	Multiple	No age restriction	2016-2021	10 (2 of which classified as WES*)	WES	This paper reviews MSAC decisions for genomic sequencing. The two MCAS applications identified as WES were application numbers 1476, which was included as a CUA [12], and 1449, which was not included as explained in the table footnote.*	[135]
Health technology assessment of whole genome sequencing in the diagnosis	Nurchis, Riccardi, & Damiani	2022	Scoping review	yes	Costs & effects	Diagnostic tool	No age restriction	Not specified/applied	7	WES + WGS (humans only)	The study identified seven HTAs of WGS for diagnosing diseases of likely genetic origin: one full HTA report; four rapid reviews; two other types of study. Four of seven HTA reports were from Canada. The	[136]

Paper Name	Paper Authors	Publication Year	Type of review	Structured search strategy	Study Types	Disease context	Age restriction on patient population	Range of publication years in search strategy	Number of studies included	Sequencing methods	Summary of findings	Reference
of genetic disorders: a scoping review of the literature											main finding was that there is a limited number of HTA reports for WGS.	
Incremental net benefit of whole genome sequencing for newborns and children with suspected genetic disorders: Systematic review and meta-analysis of cost-effectiveness evidence	Nurchis, Riccardi et al.	2022	Systematic review and meta-analysis	yes	Costs & effects	Diagnostic tool	No age restriction	2015-2021	4	WES + WGS (humans only)	Meta-analysis of incremental net benefit of WGS vs WES/CMA. The pooled INB of WGS was $4,073 vs WES and $6,003 vs CMA. However, only four studies were included in the meta-analysis, all from Canada.	[137]
Genome-Wide Sequencing for Unexplained Developmental Disabilities or Multiple Congenital Anomalies: A Health Technology Assessment [Section titled 'Economic Evidence']	Ontario Health	2020	HTA [literature search section]	yes	Costs & effects	Diagnostic tool	No age restriction	2008-2019	8	WES + WGS (humans only)	This literature search was a section of an HTA report evaluating WES/WGS in people with unexplained developmental disabilities or multiple congenital anomalies. It found eight studies which assessed the cost-effectiveness of WES or WGS compared with standard testing, and characterised the evidence base as "still very limited". "*Cost estimates ranged from $1,568 to $3,808 per sample for whole exome sequencing of a proband; $3,143 to $6,899 per sample for whole exome sequencing in trio testing; $3,350 to $4,274 per sample for whole genome sequencing of a proband, and $6,556 to $8,096 per sample for whole genome sequencing in trio testing*." Cost estimates were consistent across regions but cost-effectiveness estimates varied widely.	[48]
A systematic review of economic evaluations of whole-genome sequencing for the	Price et al.	2023	Systematic review	yes	Costs & effects	Pathogen screening	No age restriction	1991-2021	9 total; 5 for hospital surveillance	Only pathogen WGS	All of the included studies assessing the use of pathogen WGS for hospital surveillance found that it was cost-effective over comparators. The review also found a high degree of heterogeneity between results of different studies. The estimated cost of pathogen	[138]

Paper Name	Paper Authors	Publication Year	Type of review	Structured search strategy	Study Types	Disease context	Age restriction on patient population	Range of publication years in search strategy	Number of studies included	Sequencing methods	Summary of findings	Reference
surveillance of bacterial pathogens											WGS from included studies ranged from $72.13 to $470.37, with a mean of cost of $194.46.	
Microcosting diagnostic genomic sequencing: A systematic review	Santos Gonzalez et al.	2023	Systematic review	yes	Microcost analyses only	Diagnostic tool	No age restriction	Up until March 2022	7	WES + WGS (humans only)	Estimated costs ranged from USD$716 to USD$4,817 per patient for exome sequencing and USD$2,094 per patient to USD$9,706 per trio for genome sequencing. Studied differed in the level of detail provided when disaggregating costs by stage.	[139]
Are whole-exome and whole-genome sequencing approaches cost-effective? A systematic review of the literature	Schwarze et al.	2018	Systematic review	yes	Costs & effects	No restriction applied	No age restriction	2005-2016	36 [28 cost or cost-effectiveness studies]	WES + WGS (human and pathogen)	In the included studies, WES costs ranged from $555/£382 to $5,169/£3,592, human WGS costs ranged from $1,906/£1,312 to $24,810/£17,243, and pathogen WGS costs ranged from £40 to £487. There was limited economic evidence for or against the broad expansion of WES/WGS, as cost-effectiveness was dependent on context-specific factors.	[1]
Clinical Application of Genome and Exome Sequencing as a Diagnostic Tool for Pediatric Patients: a Scoping Review of the Literature	Smith, Swint et al.	2019	Scoping review	yes	Costs & effects	Diagnostic tool	Paediatric patients (probands <19 years old)	2009-2017	171 [4 economic evaluations]	WES + WGS (humans only)	Most studies identified were not economic evaluations. All of the four economic evaluations that were included only considered WES. The findings suggested that exome sequencing can be cost-effective and even cost-saving.	[140]
Economic evaluations of whole-genome sequencing for pathogen identification in public health surveillance and health-care-associated infections: a systematic review	Tran et al.	2023	Systematic review	yes	Costs & effects	Pathogen screening	No age restriction	Not well reported. For one database, the search went up to Nov 2021; others are not specified. The included studies range from 2014 to Dec. 2021.	19	Only pathogen WGS	The review characterized the existing economic evidence to support pathogen WGS as a diagnostic and surveillance tool as "insufficient and of low quality". It found that the existing economic evidence base is insufficient to support broad expansion of pathogen WGS.	[141]

Paper Name	Paper Authors	Publication Year	Type of review	Structured search strategy	Study Types	Disease context	Age restriction on patient population	Range of publication years in search strategy	Number of studies included	Sequencing methods	Summary of findings	Reference
Allocating healthcare resources to genomic testing in Canada: latest evidence and current challenges	Weymann et al.	2022	Structured literature review	yes	Costs & effects	Precision oncology	No age restriction	2005-2018	25 (8 of which WGS and/or WES)	WES + WGS (humans only)	This study reviews economic evaluations of genomic testing (NGS) for precision oncology in Canada. It notes the evidentiary challenges for these evaluations, such as coving all relevant outcomes, addressing uncertainty, and improving consistency. In the included studies, the cost-effectiveness of WGS and/or WES was sensitive to which cost-effectiveness threshold was used.	[142]
Rapid Genome-wide Testing: A Review of Clinical Utility, Cost-Effectiveness, and Guidelines	Young & Argáez	2019	Review (CADTH report)	yes	Costs & effects	Diagnostic tool	No age restriction	2014-2019	2 studies of clinical utility; 0 cost-effectiveness studies	WES + WGS (humans only)	This is a CADTH review of the cost-effectiveness and clinical utility of 'genome-wide testing', which included WES/WGS as well as panel tests. It identified no relevant cost-effectiveness studies.	[143]

Acronyms: CADTH: Canadian Agency for Drugs and Technologies in Health; MSAC: Medical Services Advisory Committee; NGS: next-generation sequencing; WGS: whole genome sequencing; WES: whole exome sequencing; ACMG: American College of Medical Genetics; HTA: health technology assessment; INB: incremental net benefit.
Note: Costs and ICERs were not converted into a common currency or currency year.
*MCAS 1449 ([144]) was listed as targeted WES in the review and was considered for inclusion via snowballing. However, the report was not included because, as described in the Public Summary Document, other methods (such as targeted multi-gene panels) could be used instead of WES, and supporting trials used NGS or Sanger sequencing, not WES.
**Dragojlovic et al., 2017, was included both as a primary research article and a review article because the report contained both a literature review and a full economic evaluation.

Supplementary Appendix 9:

Figure 9A: Time trends in the publication years of included studies: Number of publications by year, 2011-2024

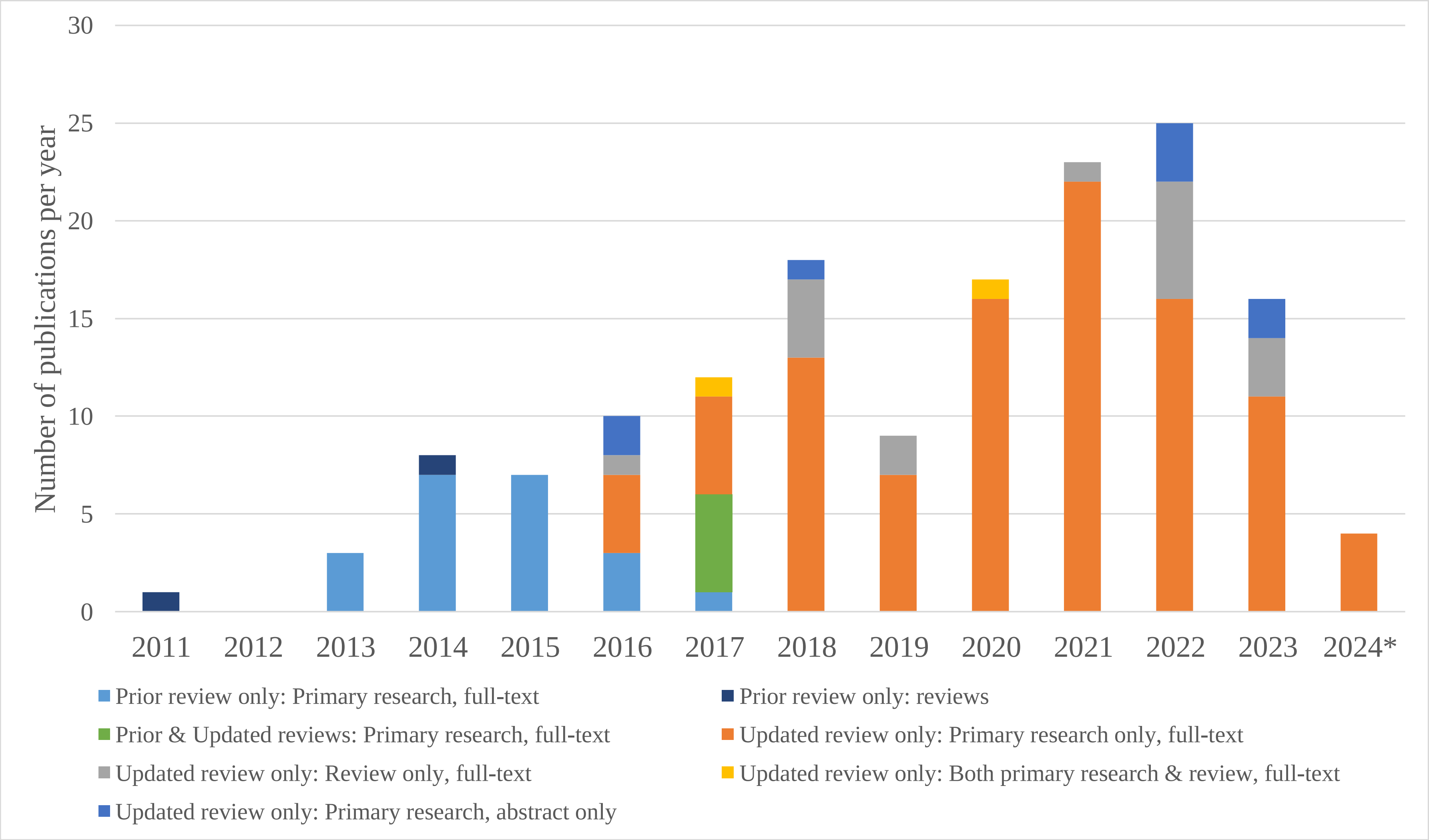

Note: No studies were included in the prior review from 2005-2010. *: The cut-off date for the search was 7 May, 2024.

Figure 9B: Time trends in the publication years of included studies: Cumulative publications by year, 2011-2024

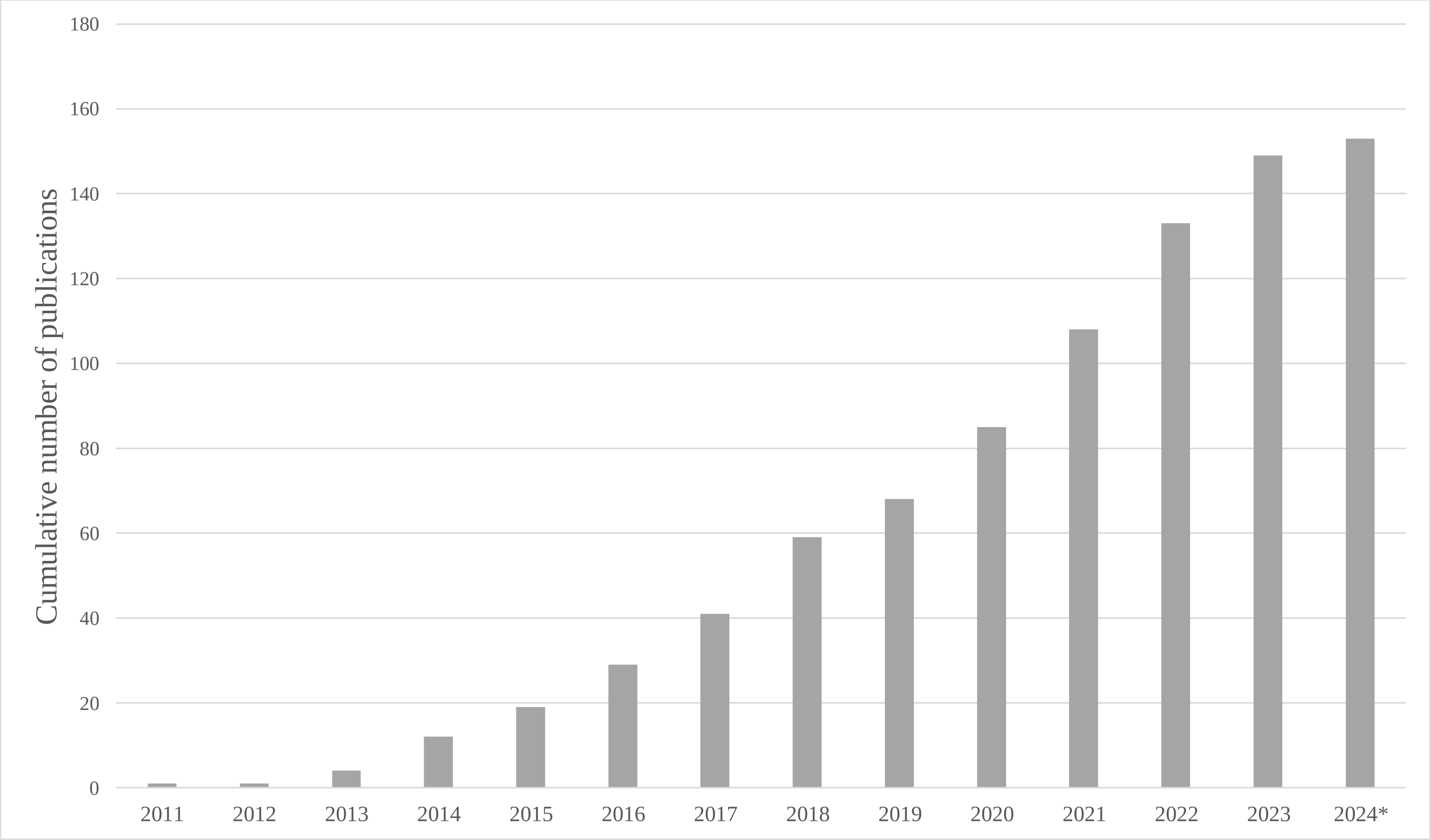

Note: No studies were included in the prior review from 2005-2010. *: The cut-off date for the search was 7 May, 2024.

Supplementary Appendix 10:

PRISMA 2020 REPORTING CHECKLIST:

Section and Topic	Item #	Checklist item	Location where item is reported
**TITLE**			
Title	1	Identify the report as a systematic review.	Highlighted in red: *The cost and cost-effectiveness of whole-exome and whole-genome sequencing: A systematic literature review*
**ABSTRACT**			
Abstract	2	See the PRISMA 2020 for Abstracts checklist.	Supplementary Appendix 1.
**INTRODUCTION**			
Rationale	3	Describe the rationale for the review in the context of existing knowledge.	Introduction
Objectives	4	Provide an explicit statement of the objective(s) or question(s) the review addresses.	Introduction
**METHODS**			
Eligibility criteria	5	Specify the inclusion and exclusion criteria for the review and how studies were grouped for the syntheses.	Methods sub-section titled: “Paper Inclusion and Exclusion Criteria”
Information sources	6	Specify all databases, registers, websites, organisations, reference lists and other sources searched or consulted to identify studies. Specify the date when each source was last searched or consulted.	Methods sub-section titled “Search strategy”
Search strategy	7	Present the full search strategies for all databases, registers and websites, including any filters and limits used.	Supplementary Appendix
Selection process	8	Specify the methods used to decide whether a study met the inclusion criteria of the review, including how many reviewers screened each record and each report retrieved, whether they worked independently, and if applicable, details of automation tools used in the process.	Methods sub-section titled: “Study Selection and Data Extraction”
Data collection process	9	Specify the methods used to collect data from reports, including how many reviewers collected data from each report, whether they worked independently, any processes for obtaining or confirming data from study investigators, and if applicable, details of automation tools used in the process.	Methods sub-section titled: “Study Selection and Data Extraction”
Data items	10a	List and define all outcomes for which data were sought. Specify whether all results that were compatible with each outcome domain in each study were sought (e.g. for all measures, time points, analyses), and if not, the methods used to decide which results to collect.	Methods sub-section titled: “Study Selection and Data Extraction”
	10b	List and define all other variables for which data were sought (e.g. participant and intervention characteristics, funding sources). Describe any assumptions made about any missing or unclear information.	Methods sub-section titled: “Study Selection and Data Extraction”

Section and Topic	Item #	Checklist item	Location where item is reported
Study risk of bias assessment	11	Specify the methods used to assess risk of bias in the included studies, including details of the tool(s) used, how many reviewers assessed each study and whether they worked independently, and if applicable, details of automation tools used in the process.	Not applicable.
Effect measures	12	Specify for each outcome the effect measure(s) (e.g. risk ratio, mean difference) used in the synthesis or presentation of results.	Methods sub-section titled: "Data analysis and reporting"
Synthesis methods	13a	Describe the processes used to decide which studies were eligible for each synthesis (e.g. tabulating the study intervention characteristics and comparing against the planned groups for each synthesis (item #5)).	Not applicable.
	13b	Describe any methods required to prepare the data for presentation or synthesis, such as handling of missing summary statistics, or data conversions.	Methods for currency indexing and conversions are reported in the Methods sub-section titled: "Data analysis and reporting"
	13c	Describe any methods used to tabulate or visually display results of individual studies and syntheses.	Methods sub-section titled: "Data analysis and reporting"
	13d	Describe any methods used to synthesize results and provide a rationale for the choice(s). If meta-analysis was performed, describe the model(s), method(s) to identify the presence and extent of statistical heterogeneity, and software package(s) used.	Methods sub-section titled: "Data analysis and reporting"
	13e	Describe any methods used to explore possible causes of heterogeneity among study results (e.g. subgroup analysis, meta-regression).	Not applicable.
	13f	Describe any sensitivity analyses conducted to assess robustness of the synthesized results.	Not applicable.
Reporting bias assessment	14	Describe any methods used to assess risk of bias due to missing results in a synthesis (arising from reporting biases).	Not applicable.
Certainty assessment	15	Describe any methods used to assess certainty (or confidence) in the body of evidence for an outcome.	Not applicable.
**RESULTS**			
Study selection	16a	Describe the results of the search and selection process, from the number of records identified in the search to the number of studies included in the review, ideally using a flow diagram.	Figure 1.
	16b	Cite studies that might appear to meet the inclusion criteria, but which were excluded, and explain why they were excluded.	These were included, where relevant, in the table footnotes of the summary tables found in Supplementary Appendices … and 4,5, and 7.
Study characteristics	17	Cite each included study and present its characteristics.	All studies are cited in the Results section. Summary statistics are presented in Tables 1 and 2. Detailed study-level characteristics are presented in Supplementary Appendices 4, 5,

Section and Topic	Item #	Checklist item	Location where item is reported
			and 7.
Risk of bias in studies	18	Present assessments of risk of bias for each included study.	This was not included for each study as described in the methods.
Results of individual studies	19	For all outcomes, present, for each study: (a) summary statistics for each group (where appropriate) and (b) an effect estimate and its precision (e.g. confidence/credible interval), ideally using structured tables or plots.	A summary of each study's findings are presented in the summary tables in Supplementary Appendices 4,5, and 7. Where relevant, confidence intervals are presented.
Results of syntheses	20a	For each synthesis, briefly summarise the characteristics and risk of bias among contributing studies.	Not applicable, but certain biases are discussed in the Discussion sub-section titled "Limitations of this review"
	20b	Present results of all statistical syntheses conducted. If meta-analysis was done, present for each the summary estimate and its precision (e.g. confidence/credible interval) and measures of statistical heterogeneity. If comparing groups, describe the direction of the effect.	Not applicable.
	20c	Present results of all investigations of possible causes of heterogeneity among study results.	Not applicable.
	20d	Present results of all sensitivity analyses conducted to assess the robustness of the synthesized results.	Not applicable.
Reporting biases	21	Present assessments of risk of bias due to missing results (arising from reporting biases) for each synthesis assessed.	Not applicable.
Certainty of evidence	22	Present assessments of certainty (or confidence) in the body of evidence for each outcome assessed.	Not applicable.
**DISCUSSION**			
Discussion	23a	Provide a general interpretation of the results in the context of other evidence.	Discussion sub-section titled "Strengths and limitations of the evidence base"
	23b	Discuss any limitations of the evidence included in the review.	Discussion sub-section titled "Strengths and limitations of the evidence base"
	23c	Discuss any limitations of the review processes used.	Discussion sub-section titled "Limitations of this review"
	23d	Discuss implications of the results for practice, policy, and future research.	Discussion sub-section titled "Conclusion"
**OTHER INFORMATION**			
Registration and	24a	Provide registration information for the review, including register name and registration number, or state that the	Section: "Materials and

Section and Topic	Item #	Checklist item	Location where item is reported
protocol		review was not registered.	Methods:", subsection: "Search Strategy and Study Selection:", paragraph 1.
	24b	Indicate where the review protocol can be accessed, or state that a protocol was not prepared.	Section: "Materials and Methods:", subsection: "Search Strategy and Study Selection:", paragraph 1.
	24c	Describe and explain any amendments to information provided at registration or in the protocol.	No substantive changes were made to the protocol.
Support	25	Describe sources of financial or non-financial support for the review, and the role of the funders or sponsors in the review.	Section titled: "Funding" disclosure
Competing interests	26	Declare any competing interests of review authors.	Section titled: "Conflicts of interest"
Availability of data, code and other materials	27	Report which of the following are publicly available and where they can be found: template data collection forms; data extracted from included studies; data used for all analyses; analytic code; any other materials used in the review.	Section titled: "Availability of data, code, or other materials:"

PRISMA 2020 ABSTRACT CHECKLIST:

Section and Topic	Item #	Checklist item	Reported (Yes/No)
**TITLE**			
Title	1	Identify the report as a systematic review.	Yes – "systematic literature review" is in the title.
**BACKGROUND**			
Objectives	2	Provide an explicit statement of the main objective(s) or question(s) the review addresses.	Yes – "Purpose" section.
**METHODS**			
Eligibility criteria	3	Specify the inclusion and exclusion criteria for the review.	Yes – "Methods" section.
Information sources	4	Specify the information sources (e.g. databases, registers) used to identify studies and the date when each was last searched.	Yes – "Methods" section.
Risk of bias	5	Specify the methods used to assess risk of bias in the included studies.	Not applicable.
Synthesis of results	6	Specify the methods used to present and synthesise results.	Yes – briefly described in "Methods" section.
**RESULTS**			
Included studies	7	Give the total number of included studies and participants and summarise relevant characteristics of studies.	Total number of included studies: yes – "Results" section; total number of participants: not applicable
Synthesis of results	8	Present results for main outcomes, preferably indicating the number of included studies and participants for each. If meta-analysis was done, report the summary estimate and confidence/credible interval. If comparing groups,	Results for main outcomes: yes – "Results" section; Meta-analysis:

Section and Topic	Item #	Checklist item	Reported (Yes/No)
		indicate the direction of the effect (i.e. which group is favoured).	not applicable; Comparison of groups: not applicable.
**DISCUSSION**			
Limitations of evidence	9	Provide a brief summary of the limitations of the evidence included in the review (e.g. study risk of bias, inconsistency and imprecision).	Yes – "Discussion" section.
Interpretation	10	Provide a general interpretation of the results and important implications.	Yes – "Discussion" section.
**OTHER**			
Funding	11	Specify the primary source of funding for the review.	Yes – "Funding and Registration" section.
Registration	12	Provide the register name and registration number.	Yes – "Funding and Registration" section.

From: Page MJ, McKenzie JE, Bossuyt PM, Boutron I, Hoffmann TC, Mulrow CD, et al. The PRISMA 2020 statement: an updated guideline for reporting systematic reviews. BMJ 2021;372:n71. doi: 10.1136/bmj.n71. This work is licensed under CC BY 4.0. To view a copy of this license, visit https://creativecommons.org/licenses/by/4.0/

References

1. Schwarze, K., et al., *Are whole-exome and whole-genome sequencing approaches cost-effective? A systematic review of the literature.* Genetics in Medicine, 2018. **20**(10): p. 1122-1130.
2. Shemilt, I., T. James, and M. Marcello, *A web-based tool for adjusting costs to a specific target currency and price year.* Evidence & Policy, 2010. **6**(1): p. 51-59.
3. OFX. *Yearly average rates*. 2025 13 April 2025; Available from: https://www.ofx.com/en-gb/forex-news/historical-exchange-rates/yearly-average-rates/.
4. Consumer Price Inflation Team, *CPI Index 06: Health 2015=100*, Office of National Statistics, Editor. 2025.
5. U.S. Bureau of Labor Statistics, *Consumer Price Index for All Urban Consumers: Medical Care in U.S. City Average [CPIMEDSL]*. 2025, received from FRED, Federal Reserve Bank of St. Louis.
6. Avram, C.M., et al., *Cost-Effectiveness of Exome Sequencing versus Targeted Gene Panels for Prenatal Diagnosis of Fetal Effusions and Non-Immune Hydrops Fetalis.* American Journal of Obstetrics & Gynecology MFM, 2022. **4**(6): p. 100724.
7. Crawford, S.A., et al., *Diagnosing newborns with suspected mitochondrial disorders: an economic evaluation comparing early exome sequencing to current typical care.* Genet Med, 2021. **23**(10): p. 1854-1863.
8. Ding, Z., *Cost-effectiveness analysis of a whole genome sequencing test compared to the standard care among patients with hospital-acquired bacteremia*, in *Graduate School of Public Health*. 2017, University of Pittsburgh: Unpublished.
9. Dymond, A., et al., *Genomic surveillance of methicillin-resistant staphylococcus aureus: A mathematical early modelling study of cost effectiveness.* Antimicrobial Resistance and Infection Control. Conference: 5th International Conference on Prevention and Infection Control, ICPIC, 2019. **8**(Supplement 1).
10. Elliott, T.M., et al., *Cost-effectiveness analysis of whole-genome sequencing during an outbreak of carbapenem-resistant Acinetobacter baumannii.* Antimicrobial Stewardship & Healthcare Epidemiology : ASHE., 2021. **1**(1): p. e62.
11. Lavelle, T.A., et al., *Cost-effectiveness of exome and genome sequencing for children with rare and undiagnosed conditions.* Genet Med, 2022. **24**(6): p. 1349-1361.
12. Medical Services Advisory Committee, *Application No. 1476 - Genetic testing for childhood syndromes*. 2019, Department of Health: Australia.
13. Mfumbilwa, Z.A., et al., *Exploring the Cost Effectiveness of a Whole-Genome Sequencing-Based Biomarker for Treatment Selection in Patients with Advanced Lung Cancer Ineligible for Targeted Therapy.* Pharmacoeconomics, 2024. **42**(4): p. 419-434.
14. Mugwagwa, T., I. Abubakar, and P.J. White, *Using molecular testing and whole-genome sequencing for tuberculosis diagnosis in a low-burden setting: a cost-effectiveness analysis using transmission-dynamic modelling.* Thorax, 2021. **76**(3): p. 281-291.
15. Sanford Kobayashi, E., et al., *Cost Efficacy of Rapid Whole Genome Sequencing in the Pediatric Intensive Care Unit.* Frontiers in Pediatrics, 2021. **9**: p. 809536.
16. Schofield, D., et al., *Long-term economic impacts of whole exome sequencing for suspected monogenic disorders.* Twin Research and Human Genetics, 2019. **21(5)**: p. 404.
17. Simons, M., et al., *Early Cost Effectiveness of Whole-Genome Sequencing as a Clinical Diagnostic Test for Patients with Inoperable Stage IIIB,C/IV Non-squamous Non-small-Cell Lung Cancer.* Pharmacoeconomics, 2021. **39**(12): p. 1429-1442.
18. Simons, M., et al., *Cost-Effectiveness and Budget Impact of Future Developments With Whole-Genome Sequencing for Patients With Lung Cancer.* Value in Health, 2023. **26**(1): p. 71-80.
19. Stark, Z., et al., *Does genomic sequencing early in the diagnostic trajectory make a difference? A follow-up study of clinical outcomes and cost-effectiveness.* Genetics in Medicine, 2019. **21**(1): p. 173-180.

20. Aaltio, J., et al., *Cost-effectiveness of whole-exome sequencing in progressive neurological disorders of children.* European Journal of Paediatric Neurology, 2022. **36**: p. 30-36.
21. Becherucci, F., et al., *A Clinical Workflow for Cost-Saving High-Rate Diagnosis of Genetic Kidney Diseases.* Journal of the American Society of Nephrology, 2023. **34**(4): p. 706-720.
22. Chen, Z., et al., *Cost-effectiveness analysis of using the TBX6-associated congenital scoliosis risk score (TACScore) in genetic diagnosis of congenital scoliosis.* Orphanet Journal Of Rare Diseases, 2020. **15**(1): p. 250.
23. Cheng, X., et al., *Comparison of different genetic testing modalities applied in paediatric patients with steroid-resistant nephrotic syndrome.* Italian Journal of Pediatrics, 2024. **50**(1): p. 85.
24. Davidson, A.L., et al., *The clinical utility and costs of whole-genome sequencing to detect cancer susceptibility variants—a multi-site prospective cohort study.* Genome Medicine, 2023. **15**(1): p. 74.
25. Degeling, K., et al., *Cost-Effectiveness of Alternative Diagnostic Testing Pathways with Whole Exome Sequencing (WES) in a Rare Disease Patient Population: The Canadian Care-for-Rare SOLVE (SOLVE) Multi-Centre Observational Cohort.* Value in Health, 2022. **25(7 Supplement)**: p. S344.
26. Degeling, K., et al., *EE171 Time to Diagnosis and Cost Effectiveness of Whole Exome Sequencing (WES) Position in the Diagnostic Pathways of Patients with Suspected Rare Genetic Disease.* Value in Health, 2023. **26(6 Supplement)**: p. S90.
27. Diaby, K., et al., *EE144 Real-World Economic Evaluation of Prospective Rapid Whole-Genome Sequencing Compared to a Matched Retrospective Cohort of Critically Ill Pediatric Patients in the United States.* Value in Health, 2022. **25(7 Supplement)**: p. S362.
28. Diaby, V., et al., *Real-world economic evaluation of prospective rapid whole-genome sequencing compared to a matched retrospective cohort of critically ill pediatric patients in the United States.* Pharmacogenomics Journal, 2022. **22**(4): p. 223-229.
29. Downie, L., et al., *Exome sequencing for isolated congenital hearing loss: a cost-effectiveness analysis.* The Laryngoscope, 2021. **131**(7): p. E2371-E2377.
30. Dragojlovic, N., et al., *The cost and diagnostic yield of exome sequencing for children with suspected genetic disorders: a benchmarking study.* Genetics in Medicine, 2018. **20**(9): p. 1-9.
31. Ewans, L.J., et al., *Whole exome and genome sequencing in mendelian disorders: a diagnostic and health economic analysis.* Eur J Hum Genet, 2022. **30**(10): p. 1121-1131.
32. Ewans, L.J., et al., *Whole-exome sequencing reanalysis at 12 months boosts diagnosis and is cost-effective when applied early in Mendelian disorders.* Genetics in Medicine, 2018. **20**(12): p. 1564-1574.
33. Howell, K.B., et al., *A population-based cost-effectiveness study of early genetic testing in severe epilepsies of infancy.* Epilepsia, 2018. **59**(6): p. 1177-1187.
34. Stinnett, A.A. and A.D. Paltiel, *Estimating CE Ratios under Second-order Uncertainty:The Mean Ratio versus the Ratio of Means.* Medical Decision Making, 1997. **17**(4): p. 483-489.
35. Incerti, D., et al., *Cost-effectiveness of genome sequencing for diagnosing patients with undiagnosed rare genetic diseases.* Genetics in Medicine, 2022. **24**(1): p. 109-118.
36. Jayasinghe, K., et al., *Cost-Effectiveness of Targeted Exome Analysis as a Diagnostic Test in Glomerular Diseases.* KI Reports, 2021. **6**(11): p. 2850-2861.
37. Tsiplova, K., R.M. Zur, and W.J. Ungar, *A microcosting and cost-consequence analysis of genomic testing strategies in autism spectrum disorder*. 2016, Technology Assessment at SickKids (TASK): Canada.
38. Jegathisawaran, J., K. Tsiplova, and W.J. Ungar, *A microcosting and cost-consequence analysis of genomic testing strategies (including trios) in autism spectrum disorder: an update*. 2019, Technology Assessment at SickKids (TASK): Canada.
39. Jegathisawaran, J., et al., *Determining accurate costs for genomic sequencing technologies-a necessary prerequisite.* Journal of Community Genetics, 2020. **11**(2): p. 235-238.

40. Kodabuckus, S.S., et al., *Exome sequencing for prenatal detection of genetic abnormalities in fetal ultrasound anomalies: an economic evaluation.* Fetal diagnosis and therapy, 2020. **47**(7): p. 554-564.
41. Kumar, P., et al., *Method for Economic Evaluation of Bacterial Whole Genome Sequencing Surveillance Compared to Standard of Care in Detecting Hospital Outbreaks.* Clinical Infectious Diseases, 2021. **73**(1): p. e9-e18.
42. Li, C., et al., *Cost-effectiveness of genome-wide sequencing for unexplained developmental disabilities and multiple congenital anomalies.* Genetics in Medicine, 2021. **23**(3): p. 451-460.
43. Milverto, J., et al., *MSAC application no. 1680.* 2021, Adelaide Health Technology Assessment (AHTA): Australia.
44. Morona, J., M. Demir, and S. Newton, *MSAC application no. 1675 -- Whole Genome Sequencing for the diagnosis of mitochondrial disease*. 2023, Adelaide Health Technology Assessment (AHTA): Australia.
45. Newton, S., et al., *MSAC application no. 1646: Whole genome sequencing of antimicrobial-resistant pathogens*. 2021, Adelaide Health Technology Assessment (AHTA): Australia.
46. Nurchis, M.C., et al., *Cost-Effectiveness of Whole-Genome vs Whole-Exome Sequencing Among Children With Suspected Genetic Disorders.* JAMA Network Open, 2024. **7**(1): p. e2353514.
47. Nurchis, M.C., et al., *Bayesian cost-effectiveness analysis of Whole genome sequencing versus Whole exome sequencing in a pediatric population with suspected genetic disorders.* European Journal of Health Economics, 2023. **17**: p. 17.
48. Ontario Health, *Genome-Wide Sequencing for Unexplained Developmental Disabilities or Multiple Congenital Anomalies: A Health Technology Assessment.* 2020, Ontario Health: Canada.
49. Palmer, E.E., et al., *Integrating exome sequencing into a diagnostic pathway for epileptic encephalopathy: Evidence of clinical utility and cost effectiveness.* Molecular Genetics & Genomic Medicine, 2018. **6**(2): p. 186-199.
50. Regier, D.A., et al., *Real-world diagnostic outcomes and cost-effectiveness of genome-wide sequencing for developmental and seizure disorders: Evidence from Canada.* Genetics in Medicine, 2024. **26**(4): p. 101069.
51. Runheim, H., et al., *The cost-effectiveness of whole genome sequencing in neurodevelopmental disorders.* Scientific Reports, 2023. **13**(1): p. 6904.
52. Ruzhnikov, M.R., et al., *Diagnostic outcomes and relative cost of clinical whole exome sequencing.* Journal of Investigative Medicine, 2016. **64(1)**: p. 247.
53. Sagoo, G., et al., *The budget impact and cost-effectiveness of introducing whole-exome sequencing-based virtual gene panel tests into routine clinical genetics.* PHG Foundation. Cambridge, UK, 2017.
54. Sanchez Fernandez, I., et al., *Diagnostic yield of genetic tests in epilepsy: A meta-analysis and cost-effectiveness study*. Neurology, 2019. **92**(5): p. e418-e428.
55. Schofield, D., et al., *Cost-effectiveness of massively parallel sequencing for diagnosis of paediatric muscle diseases.* Npj Genomic Medicine, 2017. **2**.
56. Smith, H.S., et al., *Exome sequencing compared with standard genetic tests for critically ill infants with suspected genetic conditions.* Genetics in Medicine, 2020. **22**(8): p. 1303-1310.
57. Stark, Z., et al., *Meeting the challenges of implementing rapid genomic testing in acute pediatric care.* Genetics in Medicine, 2018. **20**(12): p. 1554-1563.
58. Stark, Z., et al., *Prospective comparison of the cost-effectiveness of clinical whole-exome sequencing with that of usual care overwhelmingly supports early use and reimbursement.* Genetics in Medicine, 2017. **19**(8): p. 867-874.
59. Strauss, K.A., et al., *Genomic diagnostics within a medically underserved population: efficacy and implications.* Genetics in Medicine, 2018. **20**(1): p. 31-41.
60. Suokas, A., Y. Yi, and A. Hirst, *Cost-Effectiveness of Earlier Whole-Genome Sequencing in Rare Genetic Childhood Epilepsy.* Value in Health, 2022. **25(12 Supplement)**: p. S68.

61. Tal-Ben Ishay, R., et al., *Diagnostic Yield and Economic Implications of Whole-Exome Sequencing for ASD Diagnosis in Israel.* Genes, 2021. **13**(1): p. 23.
62. Tan, T.Y., et al., *Diagnostic Impact and Cost-effectiveness of Whole-Exome Sequencing for Ambulant Children With Suspected Monogenic Conditions.* JAMA Pediatrics, 2017. **171**(9): p. 855-862.
63. Tsiplova, K., et al., *A microcosting and cost-consequence analysis of clinical genomic testing strategies in autism spectrum disorder.* Genetics in Medicine, 2017. **19**(11): p. 1268-1275.
64. Van Nimwegen, K., et al., *The cost-effectiveness of whole-exome sequencing in complex paediatric neurology.* Value in Health, 2016. **19(7)**: p. A695.
65. van Nimwegen, K., et al., *Health Technology Assessment of next-generation sequencing: Chapter 7: Cost-effectiveness of diagnostic whole exome sequencing in complex paediatric neurology*. 2017, Radboud University: The Netherlands.
66. Varesio, C., et al., *Diagnostic Yield and Cost-Effectiveness of "Dynamic" Exome Analysis in Epilepsy with Neurodevelopmental Disorders: A Tertiary-Center Experience in Northern Italy.* Diagnostics, 2021. **11**(6): p. 25.
67. Walsh, M., et al., *Diagnostic and cost utility of whole exome sequencing in peripheral neuropathy.* Annals of Clinical & Translational Neurology, 2017. **4**(5): p. 318-325.
68. Weymann, D., et al., *Early-stage economic analysis of research-based comprehensive genomic sequencing for advanced cancer care.* Journal of Community Genetics, 2022. **13**(5): p. 523-538.
69. Yuen, T., et al., *Cost-effectiveness of Genome and Exome Sequencing in Children Diagnosed with Autism Spectrum Disorder.* Appl Health Econ Health Policy, 2018. **16**(4): p. 481-493.
70. Abbott, M., et al., *Continuing the sequence? Towards an economic evaluation of whole genome sequencing for the diagnosis of rare diseases in Scotland.* Journal of Community Genetics, 2022. **13**(5): p. 487-501.
71. Bayle, A., et al., *Whole Exome Sequencing in Molecular Diagnostics of Cancer Decreases over Time: Evidence from a Cost Analysis in the French Setting.* European Journal of Health Economics, 2021. **22**(6): p. 855-864.
72. Christensen, K.D., et al., *Short-term costs of integrating whole-genome sequencing into primary care and cardiology settings: a pilot randomized trial.* Genet Med, 2018. **20**(12): p. 1544-1553.
73. Cirillo, L., et al., *Cost-Analysis of a Clinical Workflow for Diagnosis of Inherited Kidney Diseases.* Nephrology Dialysis Transplantation, 2022. **37(SUPPL 3)**: p. i774.
74. Dragojlovic, N., et al., *The cost trajectory of the diagnostic care pathway for children with suspected genetic disorders.* Genetics in Medicine, 2020. **22**(2): p. 292-300.
75. Gordon, L.G., et al., *Estimating the costs of genomic sequencing in cancer control.* BMC Health Services Research, 2020. **20**(1): p. 492.
76. Jegathisawaran, J., et al., *Microcosting of whole genome sequencing (WGS) of trios in heterogeneous pediatric cardiac population*. 2020, Technology Assessment at SickKids (TASK): Canada.
77. Jegathisawaran, J., et al., *Trio genome sequencing for developmental delay and pediatric heart conditions: A comparative microcost analysis.* Genetics in Medicine, 2022. **24**(5): p. 1027-1036.
78. Katragadda, S., et al., *Determining Cost-Optimal Next-Generation Sequencing Panels for Rare Disease and Pharmacogenomics Testing.* Clinical Chemistry, 2021. **67**(8): p. 1122-1132.
79. Nazeha, N., et al., *Reduced resource utilization with early use of next-generation sequencing in rare genetic diseases in an Asian cohort.* American Journal of Medical Genetics Part A, 2022. **188**(12): p. 3482-3491.
80. Pasmans, C.T.B., et al., *Micro-costing diagnostics in oncology: from single-gene testing to whole- genome sequencing.* Expert Review of Pharmacoeconomics & Outcomes Research, 2021. **21**(3): p. 413-414.
81. Elliott, A.M., et al., *RAPIDOMICS: rapid genome-wide sequencing in a neonatal intensive care unit—successes and challenges.* European Journal of Pediatrics, 2019. **178**(8): p. 1207-1218.

82. Claxton, K., *The irrelevance of inference: a decision-making approach to the stochastic evaluation of health care technologies.* Journal of Health Economics, 1999. **18**(3): p. 341-364.
83. Rodriguez Llorian, E., et al., *The effect of rapid exome sequencing on downstream health care utilization for infants with suspected genetic disorders in an intensive care unit.* Genetics in Medicine, 2022. **24**(8): p. 1675-1683.
84. Schwarze, K., et al., *The complete costs of genome sequencing: a microcosting study in cancer and rare diseases from a single center in the United Kingdom.* Genetics in Medicine, 2020. **22**(1): p. 85-94.
85. Weymann, D., et al., *The cost and cost trajectory of whole-genome analysis guiding treatment of patients with advanced cancers.* Mol Genet Genomic Med, 2017. **5**(3): p. 251-260.
86. Chen, J., et al., *An additional whole-exome sequencing study in 102 panel-undiagnosed patients: A retrospective study in a Chinese craniosynostosis cohort.* Frontiers in Genetics, 2022. **13**: p. 967688.
87. Chung, C.C.Y., et al., *Rapid whole-exome sequencing facilitates precision medicine in paediatric rare disease patients and reduces healthcare costs.* The Lancet Regional Health - Western Pacific, 2020. **1**: p. 100001.
88. Cirillo, D.M., et al., *Use of WGS in Mycobacterium tuberculosis routine diagnosis.* The International Journal of Mycobacteriology, 2016. **5 Suppl 1**: p. S252-S253.
89. Cordoba, M., et al., *Whole exome sequencing in neurogenetic odysseys: An effective, cost- and time-saving diagnostic approach.* PLoS ONE [Electronic Resource], 2018. **13**(2): p. e0191228.
90. Demos, M., et al., *Diagnostic Yield and Treatment Impact of Targeted Exome Sequencing in Early-Onset Epilepsy.* Frontiers in neurology [electronic resource]. 2019. **10**: p. 434.
91. Dimmock, D., et al., *Project Baby Bear: Rapid precision care incorporating rWGS in 5 California children's hospitals demonstrates improved clinical outcomes and reduced costs of care.* Am J Hum Genet, 2021. **108**(7): p. 1231-1238.
92. Elliott, T.M., et al., *A hybrid simulation model approach to examine bacterial genome sequencing during a hospital outbreak.* BMC Infectious Diseases, 2020. **20**(1): p. 72.
93. Elliott, T.M., et al., *Evaluating the economic effects of genomic sequencing of pathogens to prioritise hospital patients competing for isolation beds.* Australian Health Review, 2021. **45**(1): p. 59-65.
94. Ellsworth, K.A., et al., *Project Baby Bear: The first state-funded quality improvement project of rapid Whole Genome Sequencing in neonatal and pediatric intensive care units in the USA.* European Journal of Human Genetics, 2020. **28(SUPPL 1)**: p. 51-52.
95. Farnaes, L., et al., *Rapid whole-genome sequencing decreases infant morbidity and cost of hospitalization.* NPJ Genom Med, 2018. **3**: p. 10.
96. Ford, L., et al., *Cost of whole genome sequencing for non-typhoidal Salmonella enterica.* PLoS One, 2021. **16**(3): p. e0248561.
97. Forman, E.B., et al., *Cost of exome sequencing in epileptic encephalopathy: is it 'worth it'?* Arch Dis Child, 2018. **103**(3): p. 304.
98. Gordon, L.G., et al., *Budget impact analysis of routinely using whole-genomic sequencing of six multidrug-resistant bacterial pathogens in Queensland, Australia.* BMJ Open, 2021. **11**(2): p. e041968.
99. Graf, T., et al., *The effect of cancer whole exome sequencing and transcriptome analysis (cWES) on the utilization of traditional molecular diagnostic testing and overall survival in pediatric blood cancer care.* American Journal of Clinical Pathology, 2018. **149(Supplement 1)**: p. S173-S174.
100. Hayeems, R.Z., et al., *Care and cost consequences of pediatric whole genome sequencing compared to chromosome microarray.* European Journal of Human Genetics, 2017. **25**(12): p. 1303-1312.

101. Hu, X., et al., *Parallel Tests of Whole Exome Sequencing and Copy Number Variant Sequencing Increase the Diagnosis Yields of Rare Pediatric Disorders.* Frontiers in Genetics, 2020. **11**: p. 473.
102. Joshi, C., et al., *Reducing the Cost of the Diagnostic Odyssey in Early Onset Epileptic Encephalopathies.* BioMed Research International, 2016. **2016**: p. 6421039.
103. Kamboj, M., et al., *Evaluation of a Combined Multilocus Sequence Typing and Whole-Genome Sequencing Two-Step Algorithm for Routine Typing of Clostridioides difficile.* Journal of Clinical Microbiology, 2021. **59**(2): p. 10.1128/jcm.01955-20.
104. Kapol, N., et al., *Using an experiment among clinical experts to determine the cost and clinical impact of rapid whole exome sequencing in acute pediatric settings.* Frontiers in Pediatrics, 2023. **11**: p. 1204853.
105. Kingsmore, S.F., et al., *A genome sequencing system for universal newborn screening, diagnosis, and precision medicine for severe genetic diseases.* American Journal of Human Genetics, 2022. **109**(9): p. 1605-1619.
106. Klau, J., et al., *Exome first approach to reduce diagnostic costs and time - retrospective analysis of 111 individuals with rare neurodevelopmental disorders.* Eur J Hum Genet, 2022. **30**(1): p. 117-125.
107. Lee, X.J., et al., *Clinical and Economic Outcomes of Genome Sequencing Availability on Containing a Hospital Outbreak of Resistant Escherichia coli in Australia.* Value in Health, 2020. **23**(8): p. 994-1002.
108. Marshall, D.A., et al., *Costs And Effectiveness Of Whole Exome Sequencing (WES) In Patients With Unsolved Rare Disease Through The Diagnostic Pathway.* International Journal of Technology Assessment in Health Care, 2023. **39(Supplement 1)**: p. S50.
109. Masri, A. and H. Hamamy, *Cost Effectiveness of Whole Exome Sequencing for Children with Developmental Delay in a Developing Country: A Study from Jordan.* Journal of Pediatric Neurology, 2022. **20(1)**: p. 20-23.
110. Mellmann, A., et al., *Real-Time Genome Sequencing of Resistant Bacteria Provides Precision Infection Control in an Institutional Setting.* Journal of Clinical Microbiology, 2016. **54**(12): p. 2874-2881.
111. Middelburg, P., et al., *Impact of whole exome sequencing (WES) on costs and medical decision-making.* Value in Health, 2016. **19(7)**: p. A705-A706.
112. Monies, D., et al., *The clinical utility of rapid exome sequencing in a consanguineous population.* Genome Medicine, 2023. **15**(1): p. 44.
113. Montaut, S., et al., *Assessment of a Targeted Gene Panel for Identification of Genes Associated With Movement Disorders.* JAMA Neurology, 2018. **75**(10): p. 1234-1245.
114. Oberg, J.A., et al., *Implementation of next generation sequencing into pediatric hematology-oncology practice: moving beyond actionable alterations.* Genome Med, 2016. **8**(1): p. 133.
115. Olde Keizer, R., et al., *Medical costs of children admitted to the neonatal intensive care unit: The role and possible economic impact of WES in early diagnosis.* Eur J Med Genet, 2022. **65**(5): p. 104467.
116. Olde Keizer, R.A.C.M., et al., *Rapid exome sequencing as a first-tier test in neonates with suspected genetic disorder: results of a prospective multicenter clinical utility study in the Netherlands.* European Journal of Pediatrics, 2023. **182**(6): p. 2683-2692.
117. Platt, C.D., et al., *Efficacy and economics of targeted panel versus whole-exome sequencing in 878 patients with suspected primary immunodeficiency.* Journal of Allergy and Clinical Immunology, 2021. **147**(2): p. 723-726.
118. Radio, F.C., et al., *Cost-effectiveness of exome sequencing: an Italian pilot study on undiagnosed patients.* New Genetics and Society, 2019. **38(3)**: p. 249-263.
119. Sainio, M.T., et al., *Effectiveness of clinical exome sequencing in adult patients with difficult-to-diagnose neurological disorders.* Acta Neurologica Scandinavica, 2022. **145**(1): p. 63-72.

120. Sweeney, N.M., et al., *Rapid whole genome sequencing impacts care and resource utilization in infants with congenital heart disease.* NPJ Genom Med, 2021. **6**(1): p. 29.
121. van de Ven, M., et al., *Dynamic Simulation Modeling to Analyze the Impact of Whole Genome Sequencing National Implementation Scenarios in Lung cancer on Time-to-Treatment, Costs and Patient Demand.* medRxiv, 2023: p. 2023.11.10.23298371.
122. Vissers, L., et al., *A clinical utility study of exome sequencing versus conventional genetic testing in pediatric neurology.* Genetics in Medicine, 2017. **19**(9): p. 1055-1063.
123. Vrijenhoek, T., et al., *Whole-exome sequencing in intellectual disability; cost before and after a diagnosis.* European Journal of Human Genetics, 2018. **26**(11): p. 1566-1571.
124. Yang, Z., et al., *Whole genome and transcriptome sequencing in neuromuscular disorders: a diagnostic and health economic analysis.* medRxiv., 2023. **21**.
125. Zhai, Y., et al., *Incorporation of exome-based CNV analysis makes trio-WES a more powerful tool for clinical diagnosis in neurodevelopmental disorders: A retrospective study.* Human Mutation, 2021. **42**(8): p. 990-1004.
126. Kamolvisit, W., et al., *Rapid exome sequencing as the first-tier investigation for diagnosis of acutely and severely ill children and adults in Thailand.* Clinical Genetics, 2021. **100**(1): p. 100-105.
127. Sainio, M.T., et al., *Recessive PYROXD1 mutations cause adult-onset limb-girdle-type muscular dystrophy.* Journal of Neurology, 2019. **266**: p. 353-360.
128. Alam, K. and D. Schofield, *Economic evaluation of genomic sequencing in the paediatric population: a critical review.* European Journal of Human Genetics, 2018. **26**(9): p. 1241-1247.
129. Douglas, M.P., et al., *Economic evidence on identifying clinically actionable findings with whole-genome sequencing: a scoping review.* Genetics in Medicine, 2016. **18**(2): p. 111-6.
130. Douglas, M.P., et al., *Report - Cost and Clinical Utility of WES and WGS in pediatric patients with suspected genetic disease.* medRxiv., 2022. **13**.
131. Dunn, P., et al., *Next Generation Sequencing Methods for Diagnosis of Epilepsy Syndromes.* Frontiers in Genetics, 2018. **9**: p. 20.
132. Fontes Marx, M., et al., *Systematic Review of the Economic Evaluation of Returning Incidental Findings in Genomic Research.* Frontiers in public health, 2021. **9**: p. 697381.
133. Gong, J., et al., *Value-based genomics.* Oncotarget, 2018. **9**(21): p. 15792-15815.
134. Kingsmore, S.F. and F.S. Cole, *The Role of Genome Sequencing in Neonatal Intensive Care Units.* Annual Review of Genomics & Human Genetics, 2022. **23**: p. 427-448.
135. Norris, S., et al., *Evaluating genetic and genomic tests for heritable conditions in Australia: lessons learnt from health technology assessments.* Journal of Community Genetics, 2022. **13**(5): p. 503-522.
136. Nurchis, M.C., M.T. Riccardi, and G. Damiani, *Health technology assessment of whole genome sequencing in the diagnosis of genetic disorders: a scoping review of the literature.* International Journal of Technology Assessment in Health Care, 2022. **38**(1): p. e71.
137. Nurchis, M.C., et al., *Incremental net benefit of whole genome sequencing for newborns and children with suspected genetic disorders: Systematic review and meta-analysis of cost-effectiveness evidence.* Health Policy, 2022. **126**(4): p. 337-345.
138. Price, V., et al., *A systematic review of economic evaluations of whole-genome sequencing for the surveillance of bacterial pathogens.* Microbial Genomics, 2023. **9**(2): p. 02.
139. Santos Gonzalez, F., et al., *Microcosting diagnostic genomic sequencing: A systematic review.* Genet Med, 2023. **25**(6): p. 100829.
140. Smith, H.S., et al., *Clinical Application of Genome and Exome Sequencing as a Diagnostic Tool for Pediatric Patients: a Scoping Review of the Literature.* Genetics in Medicine, 2019. **21**(1): p. 3-16.
141. Tran, M., et al., *Economic evaluations of whole-genome sequencing for pathogen identification in public health surveillance and health-care-associated infections: a systematic review.* The Lancet. Microbe, 2023. **4**(11): p. e953-e962.

142. Weymann, D., et al., *Allocating healthcare resources to genomic testing in Canada: latest evidence and current challenges.* Journal of Community Genetics, 2022. **13**(5): p. 467-476.
143. Young, C. and C. Argáez, *Rapid genome-wide testing: a review of clinical utility, cost-effectiveness, and guidelines.* CADTH rapid response report: summary with critical appraisal, 2019.
144. Medical Services Advisory Committee, *1449 -- Genetic testing for Alport syndrome*. 2018, Australian Government Department of Health and Aged Care.
